# Generation and application of pseudo–long reads for metagenome assembly

**DOI:** 10.1093/gigascience/giac044

**Published:** 2022-05-17

**Authors:** Mikang Sim, Jongin Lee, Suyeon Wy, Nayoung Park, Daehwan Lee, Daehong Kwon, Jaebum Kim

**Affiliations:** Department of Biomedical Science and Engineering, Konkuk University, Seoul 05029, Republic of Korea; Department of Biomedical Science and Engineering, Konkuk University, Seoul 05029, Republic of Korea; Department of Biomedical Science and Engineering, Konkuk University, Seoul 05029, Republic of Korea; Department of Biomedical Science and Engineering, Konkuk University, Seoul 05029, Republic of Korea; Department of Biomedical Science and Engineering, Konkuk University, Seoul 05029, Republic of Korea; Department of Biomedical Science and Engineering, Konkuk University, Seoul 05029, Republic of Korea; Department of Biomedical Science and Engineering, Konkuk University, Seoul 05029, Republic of Korea

**Keywords:** next-generation sequencing, metagenomic assembly, pseudo–long read

## Abstract

**Background:**

Metagenomic assembly using high-throughput sequencing data is a powerful method to construct microbial genomes in environmental samples without cultivation. However, metagenomic assembly, especially when only short reads are available, is a complex and challenging task because mixed genomes of multiple microorganisms constitute the metagenome. Although long read sequencing technologies have been developed and have begun to be used for metagenomic assembly, many metagenomic studies have been performed based on short reads because the generation of long reads requires higher sequencing cost than short reads.

**Results:**

In this study, we present a new method called PLR-GEN. It creates pseudo–long reads from metagenomic short reads based on given reference genome sequences by considering small sequence variations existing in individual genomes of the same or different species. When applied to a mock community data set in the Human Microbiome Project, PLR-GEN dramatically extended short reads in length of 101 bp to pseudo–long reads with N50 of 33 Kbp and 0.4% error rate. The use of these pseudo–long reads generated by PLR-GEN resulted in an obvious improvement of metagenomic assembly in terms of the number of sequences, assembly contiguity, and prediction of species and genes.

**Conclusions:**

PLR-GEN can be used to generate artificial long read sequences without spending extra sequencing cost, thus aiding various studies using metagenomes.

## Background

Metagenomic sequences containing all sequenced genetic materials in environmental samples are one of the most important resources for understanding the roles of microorganisms in an environment. Metagenomic sequences have been widely used for characterizing microbial communities in various environments, such as animal organs, seawater, hydrothermal environment, plants, and soils [1–6]. In studies using metagenomic sequences, the creation of high-quality metagenomic assemblies is critical to accurately discover the composition and function of microbes within the environment. Although several metagenomic assembly algorithms have been developed [7–12], the task of metagenomic assembly remains challenging because of the complexity of metagenomic sequences consisting of sequences of many short DNA fragments of diverse species [13, 14].

The development of third-generation sequencing technologies aiming to increase sequence length has provided new opportunities for metagenomic assembly because longer sequences are more useful for resolving repetitive genome sequences and distinguishing sequences from different species [15–18]. With recently developed hybrid assemblers that use long reads together with short reads, the contiguity of genome assemblies is increased while minimizing assembly errors [19, 20]. However, the generation of long reads requires relatively higher sequencing cost than short reads, thus limiting their applications for metagenomic assembly [16]. Indeed, short read–based metagenomic assemblies have been used in many recent studies [6, 21–24].

In this situation, there was a recent effort to generate and use artificial long reads from real short reads to take advantage of the benefits of both short and long reads without needing extra sequencing cost. For example, a recently developed local *de novo* assembly tool called Konnector can generate elongated pseudo–long reads from paired-end tag sequencing data for a single genome assembly [25]. Paired-end tag pseudo–long reads generated by Konnector have been successfully used to assemble the genome of the American bullfrog [26]. However, this approach cannot be directly applied to metagenomic sequences because of the complexity of metagenomic sequences resulting from the large number of species present in the metagenomic sample and similar genomic regions with small sequence variations shared by different individuals in the same or different species [27]. To address this problem, known reference genomes of many microorganisms can be used as a valuable guide to correctly capture these subtle sequence variations when generating pseudo–long read sequences even though they cannot represent all microorganisms existing in a metagenomic sample.

As an attempt to fully utilize known reference genomes of many microbial species for metagenomic assembly, we present a new method called PLR-GEN for the generation of pseudo–long reads (PLRs) by using short reads of a metagenomic sample and genome sequences of known microbial species as input. PLR-GEN can capture subtle sequence variations originating from individual genomes of the same or different species and generate PLRs with small sequence variations. PLR-GEN was applied to short paired-end reads (2 × 101 bp) in the mock community data set in the Human Microbiome Project [28, 29], and the PLRs with N50 of 33 Kbp were generated with 0.4% error rate and 99.9% alignment rate against reference genomes in the above data set. When applied to metagenomic assembly, PLRs resulted in increased assembly contiguity without introducing assembly errors. They also improved recovery of species and genes in a metagenomic sample. This result clearly shows that PLR-GEN can generate very useful and accurate artificial long reads without spending extra sequencing cost. Thus, PLR-GEN can be successfully used for various studies involving metagenomes.

## Methods

### Generation of pseudo–long reads

**Figure 1:**
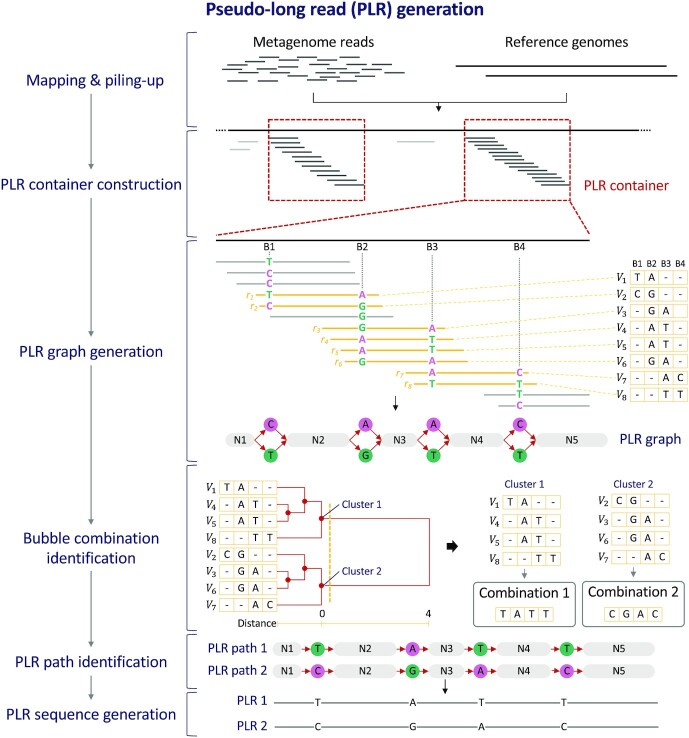
Workflow of pseudo–long read generation (PLR-GEN). Using next-generation sequencing (NGS) short reads of a metagenomic sample and reference genomes as input, NGS reads are mapped to reference genomes. Mapped reads are piled up on aligned reference genome positions (the mapping and piling-up step). Reference genome regions with continuously mapped reads are defined as PLR containers (the PLR container construction step). The PLR graph is constructed using two types of nodes, normal nodes (N1 to N5) and bubble nodes (nodes corresponding to B1 to B4), and directed edges representing the order of nodes in the PLR container (the PLR graph generation step). Vectors of bubble nodes are created and grouped by the hierarchical clustering algorithm to identify various combinations of bubble nodes (the bubble combination identification step). Together with normal nodes flanking bubble nodes, each of the different bubble node combinations is converted to a single PLR path (the PLR path identification step). Finally, a PLR sequence is constructed by concatenating nucleotides in normal and bubble nodes in each PLR path (the PLR sequence generation step).

Our method, PLR-GEN, generates PLRs through the following six steps (Fig. 1) based on next-generation sequencing short reads (single-end or paired-end reads) of a metagenomic sample and reference microbial genome sequences.

In the mapping and piling-up step, metagenomic short reads are mapped to each reference genome separately using Bowtie2 [30] with default options. All aligned reads are filtered and piled up on each reference genome using SAMtools mpileup [31] with “-q 20 –ff UNMAP,QCFAIL,DUP,SECONDARY” options. The “-q 20” option requires the use of alignments with a minimum mapping quality 20, and the “–ff UNMAP,QCFAIL,DUP,SECONDARY” option makes unmapped, low-quality, duplicated, and secondary-aligned reads skipped. The mapping quality cutoff (-q) can be changed by the user.

In the PLR container construction step, reference genomes are broken at regions without having any mapped reads. Each of the resulting sequence fragments is defined as the PLR container, which is a template for generating PLRs. Among initially generated PLR containers, small PLR containers shorter than 100 bp in length are discarded. The length cutoff can be changed by the user. Mapped reads in each PLR container are then used to generate PLR sequences in downstream steps.

In the PLR graph generation step, multiple read sequence alignment in each PLR container is converted to a graph, called a PLR graph, which consists of two types of nodes (normal and bubble) and directed edges representing the order of the nodes in the alignment. Specifically, a normal node is created from contiguous alignment columns where a single nucleotide occupies each alignment column. If an alignment column has two or more different nucleotides, then the bubble node is created for each different nucleotide. If there are only alignment columns with an identical base, then the PLR graph is generated with only normal nodes and a single PLR sequence is created. In this step, some nucleotides with very low frequency can be ignored based on the relative frequency against the most frequent nucleotide in the alignment column. By default, nucleotides with the relative frequency smaller than 0.5, which means that their absolute frequency is smaller than half of the most frequent one, are not used for generating the bubble node. The PLR graph generation panel in Fig. 1 shows an example of the PLR graph construction. Below the sequence of a reference genome (thick black line at the top) in one PLR container, multiple reads (thin lines with different colors) are aligned. Here, there are four alignment columns (B1 to B4 in Fig. 1) consisting of two different nucleotides. They are shown with aligned nucleotides, assuming that each of all other alignment columns is occupied by a single nucleotide. Two bubble nodes indicating two different aligned nucleotides in each alignment column are created. Five alignment regions flanking the above alignment columns B1 to B4 are used to create five normal nodes (named N1 to N5).

One of the excellent features of PLR-GEN is its ability to distinguish genome fragments with only a very small number of nucleotide differences from individuals of the same species or different species. This results in the generation of multiple PLR sequences with small variations in one PLR container if necessary. Generation of PLR sequences is done by finding one or more paths of nodes in the PLR graph. In the identified PLR path, only a single bubble node can be included in the path at a specific alignment position. In addition, bubble nodes at different alignment positions can be added together in the path in a dependent manner. This constraint reflects the fact that only specific combinations of variants at different alignment positions are possible, each of which corresponds to a single sequenced DNA fragment. Therefore, finding combinations of bubble nodes at different alignment positions is the main problem in PLR sequence generation. For doing that, special reads called bubble-linking reads that span two or more bubble nodes in different alignment columns (yellow lines in the PLR graph generation panel in Fig. 1) are identified, and the information of linked bubbles is then collected from the bubble-linking reads. The bubble linking information collected from one of the bubble-linking reads *r_i_* is then represented as the vector of bubble nodes with length of the number of alignment columns with the bubble nodes *B* as follows:

\begin{equation*} {V_i} = \left( {{v_{i1}}, \ldots ,{v_{iB}}} \right)\ \ {\mathrm{where}}\ {v_{ij}} \in \left\{ {{\mathrm{\text{`}A{^\prime},\text{`}C{^\prime},\text{`}G{^\prime},\text{`}T{^\prime},\text{`}-{^\prime}}}} \right\}
\end{equation*}

In this equation, the “–” symbol is used to indicate the absence of linking information of bubble nodes in that position. Vectors obtained from bubble-linking reads in the example in Fig. 1 are shown at the right-hand side of the PLR graph generation panel. For example, the link from T in B1 to A in B2 is identified by the first bubble-linking read *r*_1_ (the top-most yellow line in the PLR graph generation panel in Fig. 1). However, this read cannot provide any information for linking bubble nodes in the alignment column B3 or B4. Similarly, the link from T in B3 to T in B4 is discovered by the last bubble-linking read *r*_8_ (the bottom-most yellow line in the same panel in Fig. 1) without any information for bubble nodes in B1 or B2. Note that Fig. 1 shows an example when single-end reads are used. When paired-end reads are used, the whole DNA fragment defined by the two paired reads is treated as a single unit of the bubble-linking read.

In the bubble combination identification step, vectors of bubble nodes created from each bubble-linking read in the previous step are used to find different combinations of all bubble nodes in the PLR graph (the bubble combination identification panel in Fig. 1). The basic idea is to cluster vectors of bubble nodes based on their consistency (the same nucleotide in the same vector element) and use each resulting cluster to define a specific combination of bubble nodes. For this purpose, a hierarchical agglomerative clustering algorithm, which iteratively clusters a pair of close data (or intermediate clusters) in a hierarchical manner without needing a prespecified number of clusters [32], is used. To perform clustering, the measure of distance $D( {{V_i},\ {V_j}} )$ between two vectors of bubble nodes ${V_i}$ and ${V_j}$ for total *B* bubble nodes is defined as follows:

\begin{eqnarray*}
&& D\left( {{V_i},{V_j}} \right)\ = \ \mathop \sum \limits_{b\ = \ 1}^B {d_b}\ {\mathrm{where}}\ \ {d_b} =\nonumber\\ && \quad \left\{ {\begin{array}{@{}*{1}{c}@{}} {1,\ {\mathrm{if}}\ {v_{ib}} \ne {v_{jb}}\ {\mathrm{and}}\ {v_{ib}} \ne {\mathrm{\text{`}-{^\prime}\ and}}\ {v_{jb}} \ne {\mathrm{\text{`}-{^\prime}}}}\\ {0,\ {\mathrm{otherwise}}} \end{array}} \right.\ \
\end{eqnarray*}

Note that the above distance can be defined only for two vectors that share at least one vector element containing the same or different nucleotide in each vector. For two vectors not satisfying such a condition, an arbitrary distance larger than 1 is used to separate them to different clusters.

Based on calculated distances, hierarchical agglomerative clustering is carried out and final clusters are defined using 0 as a distance cutoff. For an example, at the bubble combination identification panel in Fig. 1, hierarchical clustering is performed for eight vectors of bubble nodes, and two clusters are finally generated. All vectors in the same cluster have an identical nucleotide at each bubble position or the “–” symbol if the bubble node at that position cannot be linked with any other bubble nodes at different positions. The latter case can happen when the distance between alignment columns with bubble nodes is too large to be linked by paired-end (or single-end) reads. From each cluster, vectors of bubble nodes are sorted by the position of the first bubble node in the PLR container. After that, the combination of bubble nodes is constructed by integrating bubble nodes at different alignment positions. At the position of an unlinked bubble node as described above, a “–” symbol is placed in the combination.

In the PLR path identification step, when all combinations of bubble nodes are identified, final PLR paths are generated by assembling flanking normal nodes and the identified consensus bubble paths. The PLR path identification panel in Fig. 1 shows two examples of PLR paths generated from two clusters obtained in the previous step.

Finally, in the PLR sequence generation step, for each PLR path, the final pseudo–long read sequence is constructed by concatenating nucleotides of normal and bubble nodes in the path. In this step, the “N” symbol is used at the position of an unlinked bubble node.

### Generation and evaluation of PLRs using the mock community data set in the Human Microbiome Project

The mock community data set in the Human Microbiome Project [15] (hereafter called the HMP data set) was downloaded and used to generate PLRs. The quality and utility of these PLRs were then evaluated. The HMP data set consists of Illumina paired-end reads (2 × 101 bp; total 3.1 Gbp of 15,396,579 pairs of reads; NCBI accession number: SRR2822457) that were used to generate PLRs and known reference genomes that were used for the true assemblies in evaluation (Supplementary Table S1).

For generating PLRs from the short reads in the HMP data set, the known reference genomes in the HMP data set were not used. Instead, reference genomes were predicted using TAMA [33], a metagenomic sequence classification tool, with default options. This is an effort to mimic a real situation when reference genomes in a metagenomic sample are not known. From a total of 5,167 reference genomes included in the reference genome database of TAMA, a total of 615 different reference genomes were predicted. They also exist in the HMP data set (Supplementary Table S2). Using these 615 microbial genomes, which covered 77% of species in the known reference genomes, PLRs were generated by PLR-GEN from the HMP data set with default options as described in Supplementary Table S3. For comparison, PLRs were also generated by IDBA-UD [12] using the same short reads as input. Even though IDBA-UD is an assembler, not the generator of PLRs, long sequences are generated by local assembly in the middle of an assembly process in IDBA-UD, which were treated as PLRs.

The quality of the generated PLRs was then assessed using MetaQUAST [34] in terms of lengths of sequences, the number of sequences, total sequence length, and the number and total length of extremely long sequences (longer than 50 Kbp). In addition, MetaQUAST was used to evaluate the quality of PLRs in comparison with the known reference genomes in the HMP data set (NCBI accession numbers in Supplementary Table S1) in terms of error rate and alignment lengths of PLRs against the known reference genomes. The error rate of PLRs was calculated based on the fraction of PLRs reported as “misassembled contigs” by MetaQUAST.

The effect and usefulness of PLRs for small sequence variation were then assessed. Among 684,388 PLR containers generated from the short reads in the HMP data set using the predicted reference genomes by TAMA, only 11,579 were used to generate more than one PLR with different combinations of bubble nodes as described in the previous subsection. From the above 11,579 PLR containers, a total of 29,291 PLRs were generated (hereafter V-PLRs). Further evaluation was performed for them. For comparison, additional PLRs, called N-PLRs, were created from V-PLRs by placing “N” at all positions corresponding to bubble nodes. V-PLRs and N-PLRs were compared by mapping them to the known reference genomes in the HMP data set using minimap2 [35] with five different mismatch penalties (4, 6, 8, 10, and 12). Output alignments were filtered by mapping quality (≥20). Reference genome coverage was calculated and compared for both V-PLRs and N-PLRs using BEDTools [36]. In addition, the read depth distribution was calculated for both V-PLRs and N-PLRs and compared by aligning them to the known reference genomes in the HMP data set using minimap2 with default options including mismatch penalty 4, which filters out the alignments with more than four mismatches.

### Evaluation of PLRs based on metagenomic assembly

For checking the usefulness of PLRs for metagenomic assembly, four different assemblers, LINKS [37], metaSPAdes [20], OPERA-MS [19], and SSPACE-Longread [38], were used to generate metagenomic assemblies for the HMP data set. In this evaluation, two versions of assemblies, a short read only assembly (SR assembly) using only short reads in the HMP data set and a PLR assembly using both short reads and PLRs, were generated and compared. In the case of metaSPAdes, the SR assembly was generated with default options, and the PLR assembly for both PLRs generated by PLR-GEN and IDBA-UD was constructed with default options except for “–nanopore.” OPERA-MS was first performed with default options. A file of intermediately generated contigs from Megahit, an embedded module in OPERA-MS, was used for the SR assembly. Final contigs generated by OPERA-MS were used for the PLR assembly with PLRs from PLR-GEN and IDBA-UD. In addition, using the SR assembly of metaSPAdes and OPERA-MS, additional long read scaffolding was carried out using LINKS [37] and SSPACE-Longread [38] with default options.

The quality of metagenomic assemblies was assessed using various statistics, including the number of sequences, assembly contiguity, and the number of misassemblies, that were calculated by MetaQUAST [34] with default options after supplying the above-known reference genomes present in the HMP data set. For each known reference genome in the HMP data set, alignment blocks of the SR and PLR assembly were generated using minimap2 embedded in MetaQUAST with the options set by MetaQUAST. Alignment blocks with a label “True” assigned by MetaQUAST were plotted using the Circlize R package [39].

### Evaluation of PLRs based on metagenomic assembly binning

Each assembly generated in the previous subsection was binned using MetaBAT2 (v 2.12.1) [40] with default options except for “–minContig 1500.” Using alignments between assemblies and the known reference genomes in the HMP data set prepared with MetaQUAST as described in the previous subsection, a species label corresponding to the known reference genome was assigned to each bin. In this step, if the sequence of a bin was aligned to more than one reference genome, the reference genome with the largest alignment coverage was chosen.

Additionally, the completeness (best: 100; worst: 0) and contamination (best: 0; worst: no upper bound) of bins were measured based on the single-copy marker gene content calculated with the lineage workflow in CheckM (v.1.1.2) [41] using default options. Because each bin is labeled independently by CheckM, the same species can be assigned to multiple bins. To compare the quality of bins at the species level, a single representative bin for each species was chosen based on the completeness score as described in a recent study [19]. All bins were categorized into four classes, “Complete,” “High Quality,” “Moderate,” and “Incomplete,” based on their quality of completeness and contamination. Specifically, a bin with completeness ≥90 and 0 contamination was defined as “Complete.” A bin with completeness ≥80 and contamination <10 was defined as “High Quality.” A bind with completeness ≥50 and contamination <20 was defined as “Moderate.” All other bins were defined as “Incomplete.” Genes in each bin were also predicted with Prodigal (v2.6.3) [42] using default options to examine gene completeness (best: 100; worst: 0) of the bin. Gene completeness was calculated based on the fraction of completely predicted genes.

## Results

### PLRs provide valuable information for metagenomic assembly in various aspects

Based on the predicted reference genomes and short paired-end reads (2 × 101 bp) in the HMP data set (Methods), a total of 704,840 PLRs with a total length of 1,248 Mbp and N50 of 33 Kbp were obtained using PLR-GEN (Table 1). Among them, 3,332 PLRs were longer than 50 Kbp (more than 500-fold longer than the input reads). Their total length was 501 Mbp. The maximum length of PLRs was 1.2 Mbp (more than 12,000-fold longer than the input reads). From the alignment of PLRs to the known reference genomes in the HMP data set, 99.9% of bases in PLRs were successfully aligned to 52% of reference genome bases. The error rate was only 0.422% (Methods). Similar evaluation was also performed for the PLRs generated by IDBA-UD (Methods, Table 1), which indicates that the quality of PLRs produced by PLR-GEN is higher than the ones by IDBA-UD in terms of all measures examined.

**Table 1:** Statistics: of pseudo–long reads generated from short paired-end reads (2 × 101 bp) using the mock community data set in the Human Microbiome Project.

Tool for PLR generation	PLR-GEN	IDBA-UD
No. of sequence (sequence in length >50 Kbp)	704,840 (3,332)	15,231 (91)
Total length (sequence in length >50 Kbp)	1,248,362,272 (501,383,852)	34,829,201 (9,667,428)
Min	100	201
Max	1,241,545	392,374
N50	32,996	15,775
Total aligned length (% of aligned bases)^1^	1,247,305,552 (99.915%)	34,757,930 (99.795%)
Genome fraction^2^	52.017%	41.793%
Error rate^3^	0.422%	3.664%

1 Total length of aligned PLR bases to the known reference genomes in the mock community data set.

2 Coverage of the known reference genomes in the mock community data set by aligned PLRs.

3 The proportion of PLRs assigned as “misassembled contigs” by MetaQUAST.

**Figure 2:**
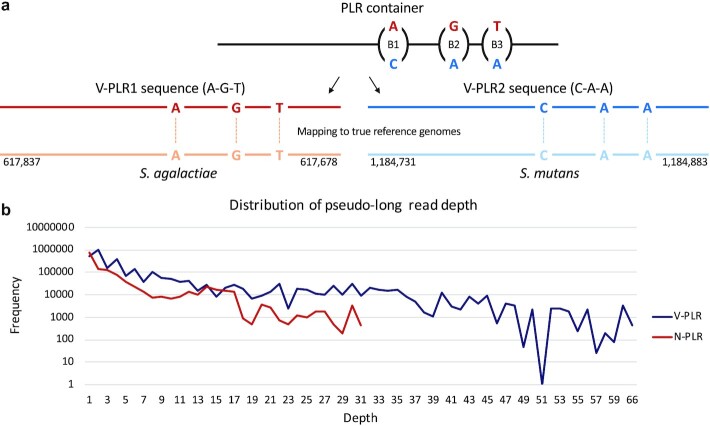
Comparison of two types of PLRs generated from the mock community data set in the Human Microbiome Project. (a) Two different V-PLRs, V-PLR1 and V-PLR2, generated from the same PLR container but with small sequence variations are mapped to two different reference genomes. (b) After mapping all V-PLRs and N-PLRs to reference genomes, their read depth distributions are plotted.

In addition to its ability to elongate short reads with very low error rate as shown above, PLR-GEN can also distinguish genome fragments with only very small sequence variations, which can originate from individual genomes of the same or different species. PLR-GEN can generate multiple PLR sequences (hereafter called V-PLRs) with small sequence variations in one PLR container that represents such a genome fragment (Methods). In the evaluation with the HMP data set, a total of 11,579 PLR containers generated 29,291 V-PLRs, which were then compared with 11,579 N-PLRs created by placing “N” at all positions of variation in V-PLRs (Methods). V-PLRs and N-PLRs were mapped to the known reference genomes in the HMP data set with various mismatch penalties, and V-PLRs could cover reference genomes more than 32 Kbp on average in comparison with N-PLRs (Supplementary Table S4). For example, in Fig. 2a, two V-PLRs created from the same PLR container having only three positions with different nucleotides were mapped to two different genomes of species, *Streptococcusagalactiae* and *Streptococcus mutans*. If a single N-PLR is generated by placing “N” at the three positions with variation, it cannot be mapped to the above two genomes unless at least three mismatches are not allowed. Depths of mapped V-PLRs and N-PLRs on all known reference genomes using the alignments with less than four mismatches were then compared (Methods; Fig. 2b). Whereas V-PLRs could be mapped with high depth up to 66×, N-PLRs failed to map to the known reference genomes with a depth larger than 31×. These experiments clearly show that PLR-GEN can capture and use subtle sequence variations when generating PLRs to cover more regions of reference genomes.

### PLRs improve the quality of short read metagenomic assembly

PLRs created from metagenomic short reads can be treated as general long reads and used in any assembly approach relying on long reads. This approach is particularly useful when only short reads are available. Researchers can take advantage of long reads for metagenomic assembly, which can be achieved by first generating PLRs from short reads by using PLR-GEN and then using PLRs for the metagenomic assembly.

**Figure 3:**
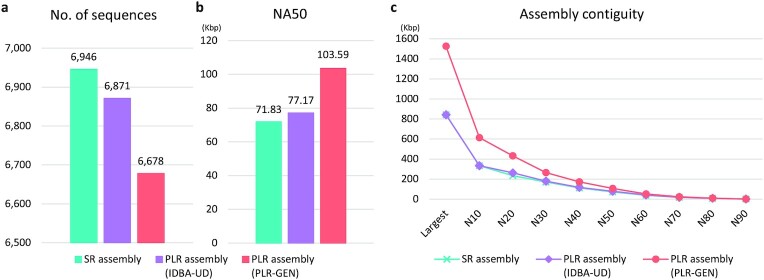
Comparison of assembly statistics generated by different assemblers for SR and PLR assemblies using the mock community data set in the Human Microbiome Project. metaSPAdes was used to create (i) the SR assembly only using short reads and (ii) the PLR assembly by further assembling it using PLRs generated by PLR-GEN and IDBA-UD. These assemblies were compared in terms of (a) the number of sequences, (b) NA50 and (c) assembly contiguity.

To examine whether PLRs can improve the quality of metagenomic assembly, short read–only metagenomic assemblies (SR assemblies) were constructed with metaSPAdes and OPERA-MS using short reads in the HMP data set. They were further assembled to make PLR assemblies using PLRs generated by PLR-GEN (Methods). When the SR assembly was further assembled by metaSPAdes, the PLRs generated by PLR-GEN could (i) reduce the number of sequences (Fig. 3a), (ii) produce longer contigs consistent with the known reference genomes in the HMP data set (Fig. 3b), and (iii) increase assembly contiguity (Fig. 3c) compared with the SR assembly. In addition, similar improvement was also observed when the further assembly was done by OPERA-MS (Supplementary Table S5). For example, the number of sequences was reduced by 10% when PLRs were used for the SR assembly with OPERA-MS (Supplementary Table S5). About a two-fold increase of length was observed for long contigs. In addition, in terms of NA50, the corrected N50 calculated after breaking the SR assembly at misassembled regions against the known reference genomes was increased 44% and 41% using PLRs for SR assemblies with metaSPAdes and OPERA-MS, respectively (Fig. 3b and Supplementary Table S5). This pattern was more prominent when additional long read–based scaffolding tools, LINKS and SSPACE-Longread, were used (Supplementary Fig. S1 and Table S5). These results clearly demonstrate that PLRs can play an important role in increasing the quality of metagenomic assembly without relying on real long reads. The PLRs generated by IDBA-UD were also used to further assemble the SR assembly by the same assembly programs used above. Even though there was also a clear improvement from the SR assembly, the quality of the resulting assemblies was worse than the ones generated by the PLRs of PLR-GEN (Fig. 3 and Supplementary Table S5).

### PLRs improve reconstructing microbial genomes and binning metagenomic assembly

**Figure 4:**
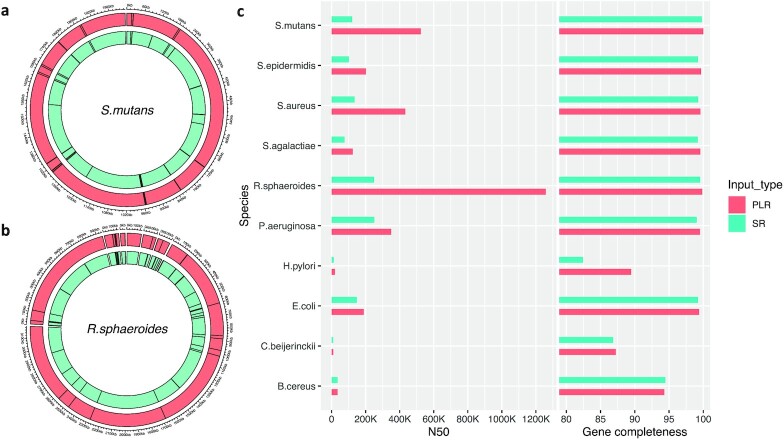
Comparison of assemblies generated by metaSPAdes using the mock community data set in the Human Microbiome Project in terms of (a, b) species genome reconstruction and (c) contig binning. SR and PLR assemblies were aligned and visualized using the Circlize R package [39] for (a) *S. mutans* and (b) *R. sphaeroides* genomes. (c) After binning contigs and finding representative bins for 10 species, their quality was measured in terms of N50 (bars in the left panel) and gene completeness (bars in the right panel).

Metagenomic assemblies can be used to reconstruct original microbial chromosome sequences, which can be further used for various downstream analyses, including metagenomic assembly binning and gene prediction. To evaluate the effect of improved metagenomic assemblies with PLRs for recovering original microbial chromosome sequences, the SR assembly and the PLR assembly generated with metaSPAdes were aligned against each of the known reference genomes in the HMP data set (Methods). In most of those reference genomes, the PLR assembly could cover more contiguous regions (outer rings in Supplementary Fig. S2) than the SR assembly (inner rings in Supplementary Fig. S2). In the case of *S. mutans* (Fig. 4a), 99.56% of its genome was covered by 21 alignment blocks created by the PLR assembly. However, 40 alignment blocks were used to cover similar genomic regions with the SR assembly. Specifically, the longest alignment block created by the PLR assembly was 524 Kbp, which was more than two-fold longer than the longest one (237 Kbp) created by the SR assembly. Similar pattern was observed in another reference species of *Rhodobacter sphaeroides* (Fig. 4b). Specifically, chromosome 2 of *R. sphaeroides* was covered by only three alignment blocks of the PLR assembly, whereas 14 alignment blocks of the SR assembly were needed to cover chromosome 2 of *R. sphaeroides*.

To examine the usefulness of PLR-assisted metagenomic assemblies in other downstream analyses, both SR and PLR assemblies were binned, a species label was assigned to each bin, and the quality of each resulting bin was then evaluated for the assigned 10 species (Methods). As shown in Fig. 4c and Supplementary Table S6, the PLR assembly increased the contiguity of binned sequences (bars in the left panel in Fig. 4c) without sacrificing bin completeness or contamination (Supplementary Table S6). In the case of gene completeness (bars in the right panel in Fig. 4c), the PLR assembly improved the quality of metagenomic bins in comparison with the SR assembly (from 95.91% to 96.86%; Fig. 4c and Supplementary Table S6). Specifically, for *the R. sphaeroides* genome, N50 was increased more than fivefold when the PLR assembly was used. In terms of bin completeness and contamination, the PLR assembly was effective in improving the bin quality of *Staphylococcus epidermidis* from “High Quality” to “Complete.” Additionally, the gene completeness was increased with PLRs for nine species-labeled bins. These findings indicate that the use of metagenomic assemblies generated with PLRs from PLR-GEN is useful for downstream analyses.

## Discussion

In this study, we present a new method, called PLR-GEN, for generating pseudo–long reads (PLRs). PLRs are artificial long reads generated from next-generation sequencing short reads by utilizing microbial reference genomes. Our method was successfully applied to short reads of 101 bp in length in the HMP data set by creating PLRs with N50 of 33 Kbp that could be almost completely aligned to the known reference genomes in the HMP data set with a very low error rate (Fig. 2 and Table 1). For metagenomic assembly, the SR assemblies created by metaSPAdes and OPERA-MS were further assembled using PLRs, leading to dramatic improvement of resulting assemblies in terms of the number of sequences and assembly contiguity (Fig. 3). Assemblies improved by PLRs were also very useful for assembly binning and reconstruction of species genome (Fig. 4). These improved species genomes resulted in increased completeness of gene prediction (Fig. 4 and Supplementary Tables S6).

Sequenced long reads can provide long-range information. They are very helpful for metagenomic assembly. However, the sequencing of long reads is more expensive and requires more carefully handled and larger amount of DNA than generating short reads. This prevents their widespread use for metagenomic assembly. Therefore, short reads are still being used for metagenomic assembly and related studies [43–45]. In such situations, it is very helpful to generate long reads by just using short reads and reference genomes without needing extra sequencing cost. PLRs generated by our method can be used in many studies involving metagenomes, including studies based on assemblies, as shown here by treating them as normal long reads such as PacBio [46] and Nanopore [47] reads.

One of the excellent features of our method is its ability to capture subtle sequence variations resulting from individual genomes in the same or different species in a metagenomic sample. This was achieved by (i) carefully identifying mapping positions of short reads occupied by multiple different nucleotides, (ii) representing them as vectors of sequence variations, and (iii) grouping them using a hierarchical clustering algorithm based on a newly designed distance measure. The effect of this feature was confirmed in comparison with PLRs generated by turning this feature off (Fig. 2). Therefore, PLRs generated by our method can also be used to discover information of haplotypes inherent in a metagenome [48].

One of the limitations of PLR-GEN is that because PLR-GEN creates PLRs by relying on given microbial reference genomes, the quality of PLRs depends on the number and quality of microbial reference genomes used. Therefore, to obtain high-quality PLRs, the environment needs to be well studied and characterized with many high-quality reference genomes. However, the improvement of assemblies by our PLRs generated using the small number of reference genomes was also observed from empirical experiments using randomly sampled reference genomes from the full reference genomes used in our study (Supplementary Tables S7 and S8). Another difficulty in metagenomic assembly is that there are multiple unknown microorganisms in a metagenomic sample sharing similar genomic regions with low sequence variations. In this situation, one important preprocessing step is to prepare the most appropriate reference genomes for a target metagenomic sample. This can be achieved by using recently developed metagenomic classifiers [33, 49–52] and collecting genomes of predicted species using those metagenomic classifiers. To this end, TAMA [33], one of the metagenomic sequence classifiers, was used to prepare a set of reference genomes in the HMP data set for making PLRs in this study. Another option is to use all microbial genomes in a public database such as NCBI, but it will take a lot of time and computer resources. However, continued accumulation of high-quality genome sequences of many microorganisms will make our method more valuable for studies involving metagenomes.

Finally, many existing metagenomic assemblies generated by only using short reads are good targets of PLR-GEN. As a future direction, those assemblies will be further improved by PLR-GEN and publicly released for being used by many researchers. In addition, PLR-GEN will be more optimized to more efficiently process a large number of reference genomes.

## Availability of supporting source code and requirements

Project name: PLR-GEN

Project home page: https://github.com/jkimlab/PLR-GEN

Operating system: Linux

Programming language: Perl

Other requirements: TAMA

RRID:SCR_022264

License: MIT

## Data Availability

The PLR-GEN package is available at https://github.com/jkimlab/PLR-GEN. Snapshots of our code and other data further supporting this work are openly available in the *GigaScience* repository, GigaDB [53].

## Additional Files

**Supplementary Table S1**. NCBI accession numbers of the known reference genomes in the HMP data set.

**Supplementary Table S2**. List of predicted reference genomes.

**Supplementary Table S3**. List of parameters of PLR-GEN used for evaluation.

**Supplementary Table S4**. Comparison of reference coverage between V-PLRs and N-PLRs.

**Supplementary Table S5**. Statistics of short read assemblies and assemblies improved by PLRs.

**Supplementary Table S6**. Quality of each bin of the SR and PLR assemblies.

**Supplementary Table S7**. Statistics of PLRs generated from short reads in the mock community data set with different sets of reference genomes.

**Supplementary Table S8**. Statistics of short read assemblies and assemblies improved by PLRs with different sets of reference genomes.

**Supplementary Fig. S1**. metaSPAdes and OPERA-MS were separately used to create (i) the SR assembly only using short reads and (ii) the PLR assembly by further assembly with long read scaffolding tools using PLRs. These two types of assemblies were compared in terms of (a) the number of sequences and (b) NA50.

**Supplementary Fig. S2**. Circos plots illustrating alignments of SR and PLR assemblies for genomes of all species in the HMP data set. Inner (green color) and outer (orange color) circles represent SR and PLR assemblies, respectively.

## List of abbreviations

HMP: Human Microbiome Project; PLRs: pseudo–long reads; SR: short read.

## Ethics approval and consent to participate

Not applicable

## Consent for publication

Not applicable

## Competing interests

The author(s) declare no competing interests.

## Funding

This paper was supported by Konkuk University Researcher Fund in 2020, a grant [2014M3C9A3063544] funded by the Ministry of Science and ICT of Korea, a grant [2019R1F1A1042018 and 2021M3H9A2097134] funded by the Ministry of Education of Korea, and a grant [PJ01334302] funded by the Rural Development Administration of Korea.

## Authors' contributions

JBK conceived and designed the study. JBK, MKS, JIL, and DHL designed the PLR-GEN algorithm. MKS implemented the pseudo–long read generation algorithm. MKS, SYW, and NYP performed experiments. MKS, SYW, NYP, DHK, and JBK interpreted the analysis results. MKS drafted the manuscript. JBK finalized the manuscript. All authors approved the final manuscript.

## Supplementary Material

giac044_GIGA-D-21-00349_Original_Submission

giac044_GIGA-D-21-00349_Revision_1

giac044_Response_to_Reviewer_Comments_Original_Submission

giac044_Reviewer_1_Report_Original_Submission Jin-Wu Nam -- 12/2/2021 Reviewed

giac044_Reviewer_1_Report_Revision_1 Jin-Wu Nam -- 3/20/2022 Reviewed

giac044_Reviewer_2_Report_Original_Submission Matthew Zachariah DeMaere, Ph.D -- 12/13/2021 Reviewed

giac044_Reviewer_2_Report_Revision_1 Matthew Zachariah DeMaere, Ph.D -- 3/27/2022 Reviewed

giac044_Supplemental_Files
