# Supplementary material for: Generation and application of pseudo–long reads for metagenome assembly
Source: Gigascience. 2022 May 17;11:giac044. doi: 10.1093/gigascience/giac044 (PMC9112764; doi:10.1093/gigascience/giac044)
Supplement: giac044_GIGA-D-21-00349_Original_Submission [file giac044_giga-d-21-00349_original_submission.pdf]

GigaScience

Generation and application of pseudo-long reads for metagenome assembly

--Manuscript Draft--

**Manuscript Number:**	GIGA-D-21-00349
**Full Title:**	Generation and application of pseudo-long reads for metagenome assembly
**Article Type:**	Technical Note
**Funding Information:**	Konkuk University Researcher Fund in 2021 — Dr Jaebum Kim<br>the Ministry of Science and ICT of Korea (NRF-2014M3C9A3063544) — Dr Jaebum Kim<br>the Ministry of Education of Korea (NRF-2019R1F1A1042018) — Dr Jaebum Kim<br>the Rural Development Administration of Korea (PJ01334302) — Dr Jaebum Kim

Funding source	Recipient
Konkuk University Researcher Fund in 2021	Dr Jaebum Kim
the Ministry of Science and ICT of Korea (NRF-2014M3C9A3063544)	Dr Jaebum Kim
the Ministry of Education of Korea (NRF-2019R1F1A1042018)	Dr Jaebum Kim
the Rural Development Administration of Korea (PJ01334302)	Dr Jaebum Kim

Abstract:

Background:
Metagenome assembly using high-throughput sequencing data is a powerful method to construct microbial genomes in environmental samples without cultivation. However, metagenome assembly, especially when only short reads are available, is a complex and challenging task because mixed genomes of multiple microorganisms constitute the metagenome. Although long read sequencing technologies have been developed and begun to be used for metagenome assembly, many metagenome studies have been performed based on short reads because long reads have higher sequencing cost and error rate than short reads.
Results:
In this study, we present a new method called PLR-GEN. It can create pseudo-long reads from metagenome short reads based on given reference genome sequences by considering small sequence variations existing in individual genomes of the same or different species. When applied to a mock community dataset in the Human Microbiome Project, PLR-GEN dramatically extended short reads in length of 101 bp to pseudo-long reads with N50 of 33 Kbp and 0.4 % error rate. The use of these pseudo-long reads generated by PLR-GEN resulted in an obvious improvement of metagenome assembly in terms of the number of sequences, assembly contiguity, and prediction of species and genes.
Conclusions:
PLR-GEN can be used to generate artificial long-read sequences without spending extra sequencing cost, thus aiding various studies using metagenomes.

Keywords: next-generation sequencing, metagenome assembly, pseudo-long read

**Corresponding Author:**	Jaebum Kim<br>Konkuk University<br>Seoul, KOREA, REPUBLIC OF
**Corresponding Author Secondary Information:**	
**Corresponding Author's Institution:**	Konkuk University
**Corresponding Author's Secondary Institution:**	
**First Author:**	Mikang Sim
**First Author Secondary Information:**	
**Order of Authors:**	Mikang Sim
	Jongin Lee
	Suyeon Wy
	Nayoung Park
	Daehwan Lee

Powered by Editorial Manager® and ProduXion Manager® from Aries Systems Corporation

	Daehong Kwon
	Jaebum Kim
**Order of Authors Secondary Information:**	
**Additional Information:**	
**Question**	**Response**
Are you submitting this manuscript to a special series or article collection?	No
**Experimental design and statistics**<br><br>Full details of the experimental design and statistical methods used should be given in the Methods section, as detailed in our Minimum Standards Reporting Checklist. Information essential to interpreting the data presented should be made available in the figure legends.<br><br>Have you included all the information requested in your manuscript?	Yes
**Resources**<br><br>A description of all resources used, including antibodies, cell lines, animals and software tools, with enough information to allow them to be uniquely identified, should be included in the Methods section. Authors are strongly encouraged to cite Research Resource Identifiers (RRIDs) for antibodies, model organisms and tools, where possible.<br><br>Have you included the information requested as detailed in our Minimum Standards Reporting Checklist?	Yes
**Availability of data and materials**<br><br>All datasets and code on which the conclusions of the paper rely must be either included in your submission or deposited in publicly available repositories (where available and ethically appropriate), referencing such data using	Yes

a unique identifier in the references and in the “Availability of Data and Materials” section of your manuscript.<br><br>Have you have met the above requirement as detailed in our Minimum Standards Reporting Checklist?	

Powered by Editorial Manager® and ProduXion Manager® from Aries Systems Corporation

Manuscript
Click here to access/download;Manuscript;manuscript_PLR-GEN_GS_vfinal.docx

Generation and application of pseudo-long reads for metagenome assembly

Author: Mikang Sim, Jongin Lee, Suyeon Wy, Nayoung Park, Daehwan lee, Daehong Kwon, and Jaebum Kim*

Department of Biomedical Science and Engineering, Konkuk University, Seoul 05029, Republic of Korea.

*To whom correspondence should be addressed.

Email addresses:

Mikang Sim: mg9022@konkuk.ac.kr

Jongin Lee: jongin33@konkuk.ac.kr

Suyeon Wy: lish96@konkuk.ac.kr

Nayoung Park: p3159@konkuk.ac.kr

Daehwan Lee: cjfcl@konkuk.ac.kr

Daehong Kwon: kwanyi01@konkuk.ac.kr

Jaebum Kim: jbkim@konkuk.ac.kr

Abstract

Background:

Metagenome assembly using high-throughput sequencing data is a powerful method to construct microbial genomes in environmental samples without cultivation. However, metagenome assembly, especially when only short reads are available, is a complex and challenging task because mixed genomes of multiple microorganisms constitute the metagenome. Although long read sequencing technologies have been developed and begun to be used for metagenome assembly, many metagenome studies have been performed based on short reads because long reads have higher sequencing cost and error rate than short reads.

Results:

In this study, we present a new method called PLR-GEN. It can create pseudo-long reads from metagenome short reads based on given reference genome sequences by considering small sequence variations existing in individual genomes of the same or different species. When applied to a mock community dataset in the Human Microbiome Project, PLR-GEN dramatically extended short reads in length of 101 bp to pseudo-long reads with N50 of 33 Kbp and 0.4 % error rate. The use of these pseudo-long reads generated by PLR-GEN resulted in an obvious improvement of metagenome assembly in terms of the number of sequences, assembly contiguity, and prediction of species and genes.

Conclusions:

PLR-GEN can be used to generate artificial long-read sequences without spending extra sequencing cost, thus aiding various studies using metagenomes.

Keywords: next-generation sequencing, metagenome assembly, pseudo-long read

Background

Metagenome sequences containing all sequenced genetic materials in environmental samples are one of the most important resources for understanding the roles of microorganisms in an environment. Metagenome sequences have been widely used for characterizing microbial communities in various environments, such as animal organs, sea water, hydrothermal environment, plants, and soils [1-6]. In studies using metagenome sequences, the creation of high-quality metagenome assemblies is critical to accurately discover microbial compositions and their functions in the environment. Although several metagenome assembly algorithms have been developed [7-12], the task of metagenome assembly remains challenging because of the complexity of metagenome sequences consisting of sequences of many short DNA fragments of diverse species [13, 14].

The development of third-generation sequencing technologies aiming to increase sequence length has provided new opportunities for metagenome assembly because longer sequences are more useful for resolving repetitive genome sequences and distinguishing sequences from different species [15-18]. With recently developed hybrid assemblers that use long-read sequences together with short-read sequences, the contiguity of genome assemblies is increased while minimizing assembly errors [19, 20]. However, long-read sequences have relatively higher error rate and sequencing cost than short-read sequences, thus limiting their applications for metagenome assembly [16]. Indeed, short read-based metagenome assemblies are still being generated and used in many recent studies [6, 21-24].

In this situation, the generation and use of artificial long reads from real short reads can be a good alternative to take advantage of benefits of both short and long reads without needing extra sequencing cost. For example, a recently developed local *de novo* assembly tool called Konnector can generate elongated pseudo-long reads from paired-end tag (PET) sequencing data for a single genome assembly [25]. PET pseudo-long reads generated by Konnector have

been successfully used to assemble the genome of the American bullfrog [26]. However, this approach cannot be directly applied to metagenome sequences because of the complexity of metagenome sequences resulting from the large number of species present in the metagenome sample and similar genomic regions with small sequence variations shared by different individuals in the same or different species [27]. To address this problem, known reference genomes of many microorganisms can be used as a valuable guide to correctly capture these subtle sequence variations when generating pseudo-long read sequences even though they cannot represent all microorganisms existing in a metagenome sample.

As an attempt to fully utilize known reference genome sequences of many microbial species for metagenome assembly, we present a new method called PLR-GEN for the generation of pseudo-long reads (PLRs) by using short reads of a metagenome sample and genome sequences of known microbial species as input. PLR-GEN can capture subtle sequence variations originating from individual genomes of the same or different species and generate PLRs with small sequence variations. PLR-GEN was applied to short paired-end reads (2 x 101 bp) in the mock community dataset in the human microbiome project [28, 29], and the PLRs with N50 of 33 Kbp were generated with 0.4 % error rate and 99.9 % alignment rate against reference genomes in the above dataset. When applied to metagenome assembly, PLRs resulted in increased assembly contiguity without introducing assembly errors. They also improved recovery of species and genes in a metagenome sample. This result clearly shows that PLR-GEN can generate very useful and accurate artificial long reads without spending extra sequencing cost. Thus, PLR-GEN can be successfully used for various studies involving metagenomes.

Methods

Generation of pseudo-long reads

Our method, PLR-GEN, can generates pseudo-long reads (PLRs) through the following six steps (Fig. 1) based on next-generation sequencing short reads (single-end or paired-end reads) of a metagenome sample and reference microbial genome sequences.

In the mapping & piling-up step, metagenome short reads are mapped to each reference genome sequences separately using Bowtie2 [30] with default options. All aligned read sequences are filtered and piled-up on each reference genome using SAMtools mpileup [31] with '-q 20 --ff UNMAP,QCFAIL,DUP,SECONDARY' options. The mapping quality cutoff (-q) can be changed by the user.

In the PLR container construction step, reference genome sequences are broken at regions without having any mapped reads. Each of the resulting sequence fragments is defined as the PLR container which is a template for generating PLRs. Among initially generated PLR containers, small PLR containers shorter than 100 bp in length are discarded. The length cutoff can be changed by the user. Mapped reads in each PLR container are then used to generate PLR sequences in downstream steps.

In the PLR graph generation step, multiple read sequence alignment in each PLR container is converted to a graph, called a PLR graph, which consists of two types of nodes (normal and bubble) and directed edges representing the order of the nodes in the alignment. Specifically, a normal node is created from contiguous alignment columns where a single nucleotide occupies each alignment column. If an alignment column has two or more different nucleotides, then the bubble node is created for each different nucleotide. If there are only alignment columns with an identical base, then PLR graph is generated with only normal nodes and a single PLR sequence is created. In this step, some nucleotides with very low frequency can be ignored based on the relative frequency against the most frequent nucleotide in the alignment column. By default, nucleotides with the relative frequency smaller than 0.5, which means that their absolute frequency is smaller than half of the most frequent one, are not used for generating

the bubble node. The PLR graph generation panel in Fig. 1 shows an example of the PLR graph construction. Below the sequence of a reference genome (thick black line at the top) in one PLR container, multiple read sequences (thin lines with different colors) are aligned. Here, there are four alignment columns (B1 to B4 in Fig. 1) consisting of two different nucleotides. They are shown with actually aligned nucleotides, assuming that each of all other alignment columns is occupied by a single nucleotide. Two bubble nodes indicating two different aligned nucleotides in each alignment column are created. Five alignment regions flanking the above alignment columns B1 to B4 are used to create five normal nodes (named N1 to N5).

One of the excellent features of PLR-GEN is its ability to distinguish genome fragments only with a very small number of nucleotide differences from individuals of the same species or different species. This results in the generation of multiple PLR sequences with small variations in one PLR container if necessary. Generation of PLR sequences is done by finding one or more paths of nodes in the PLR graph. In the identified PLR path, only a single bubble node can be included in the path at a specific alignment position. In addition, bubble nodes at different alignment positions can be added together in the path in a dependent manner. This constraint reflects the fact that only specific combinations of variants at different alignment positions are possible, each of which corresponds to a single DNA fragment sequenced. Therefore, finding combinations of bubble nodes at different alignment positions is the main problem in PLR sequence generation. For doing that, special reads called bubble-linking reads that span two or more bubble nodes in different alignment columns (yellow lines in the PLR graph generation panel in Fig. 1) are identified, and the information of linked bubbles is then collected from the bubble-linking reads. The bubble linking information collected from one of bubble-linking reads r_i is then represented as the vector of bubble nodes with length of the number of alignment columns with the bubble nodes B as follows:

$$V_i = (v_{i1}, \dots, v_{iB}) \text{ where } v_{ij} \in \{\text{'A','C','G','T','–'}\}$$

In this equation, the '-' symbol is used to indicate the absence of linking information of bubble nodes in that position. Vectors obtained from bubble-linking reads in the example in Fig. 1 are shown at the right-hand side of the PLR graph generation panel. For example, the link from T in B1 to A in B2 is identified by the first bubble-linking read r_1 (the top-most yellow line in the PLR graph generation panel in Fig. 1). However, this read cannot provide any information for linking bubble nodes in the alignment column B3 or B4. Similarly, the link from T in B3 to T in B4 is discovered by the last bubble-linking read r_8 (the bottom-most yellow line in the same panel in Fig. 1) without any information for bubble nodes in B1 or B2. Note that Fig. 1 shows an example when single reads are used. When paired-end reads are used, the whole DNA fragment defined by the two paired reads is treated as a single unit of the bubble-linking read.

In the bubble combination identification step, vectors of bubble nodes created from each of bubble-linking reads in the previous step are used to find different combinations of all bubble nodes in the PLR graph (the bubble combination identification panel in Fig. 1). The basic idea is to cluster vectors of bubble nodes based on their consistency (the same nucleotide in the same vector element) and use each resulting cluster to define a specific combination of bubble nodes. For this purpose, a hierarchical agglomerative clustering algorithm, which iteratively clusters a pair of close data (or intermediate clusters) in hierarchical manner without needing a pre-specified number of clusters [32], is used. To perform clustering, the measure of distance $D(V_i, V_j)$ between two vectors of bubble nodes V_i and V_j for total B bubble nodes is defined as follows:

$$D(V_i, V_j) = \sum_{b=1}^{B} d_b \quad \text{where } d_b = \begin{cases} 1, & \text{if } v_{ib} \neq v_{jb} \text{ and } v_{ib} \neq \text{'–' and } v_{jb} \neq \text{'–'} \\ 0, & \text{otherwise} \end{cases}$$

Note that the above distance can be defined only for two vectors that share at least one vector element containing the same or different nucleotide in each vector. For two vectors not satisfying such a condition, an arbitrary distance larger than 1 is used to separate them to

different clusters.

Based on calculated distances, hierarchical agglomerative clustering is carried out and final clusters are defined using 0 as a distance cutoff. For an example, at the bubble combination identification panel in Fig. 1, hierarchical clustering is performed for eight vectors of bubble nodes and two clusters are finally generated. All vectors in the same cluster have identical nucleotide at each bubble position or the '-' symbol if the bubble node at that position cannot be linked with any other bubble nodes at different positions. The latter case can happen when the distance between alignment columns with bubble nodes is too large to be linked by paired (or single) reads. From each cluster, vectors of bubble nodes are sorted by the position of the first bubble node in the PLR container. After that, the combination of bubble nodes is constructed by integrating bubble nodes at different alignment positions. At the position of an unlinked bubble node as described above, a '-' symbol is placed in the combination.

In the PLR path identification step, when all combinations of bubble nodes are identified, final PLR paths are generated by assembling flanking normal nodes and the identified consensus bubble paths. The PLR path identification panel in Fig. 1 shows two examples of PLR paths generated from two clusters obtained in the previous step.

Finally, in the PLR sequence generation step, for each PLR path, the final pseudo-long read sequence is constructed by concatenating nucleotides of normal and bubble nodes in the path. In this step, the 'N' symbol is used at the position of an unlinked bubble node.

Generation and evaluation of PLRs using the mock community dataset in the Human Microbiome Project

The mock community dataset in the Human Microbiome Project [15] (hereafter called the HMP dataset) was downloaded and used to generate PLRs. The quality and utility of these PLRs were then evaluated. The HMP dataset consists of Illumina paired-end reads (2 × 101 bp; total 3.1 Gbp of 15,396,579 pairs of reads; NCBI accession number: SRR2822457) and known

reference genomes (Supplementary Table S1).

For generating PLRs from the HMP dataset, the known reference genomes in the HMP dataset were not used. Instead, reference genomes were predicted using TAMA [33], a metagenome sequence classification tool, with default options. This is an effort to mimic a real situation when reference genomes in a metagenome sample are not known. From a total of 5,167 reference genomes included in the reference genome database of TAMA, a total of 615 different reference genomes were predicted. They are also existing in the HMP dataset (Supplementary Table S2). Using these 615 microbial genome sequences that covered 77 % of species in the known reference genomes, PLRs were generated by PLR-GEN from the HMP dataset with default options as described in Supplementary Table S3.

The quality of the generated PLRs was then assessed in terms of lengths of sequences, the number of sequences, total sequence length, the number and total length of extremely long sequences (longer than 50 Kbp), and N10 to N90 values (all calculated using in-house Perl script). In addition, MetaQUAST [34] was used to evaluate the quality of PLRs in comparison with the known reference genomes in the HMP dataset (NCBI accession numbers in Supplementary Table S1) in terms of error rate and alignment lengths of PLRs against the known reference genomes. The error rate of PLRs was calculated based on the fraction of PLRs reported as “misassembled contigs” by MetaQUAST.

The effect and usefulness of PLRs for small sequence variation were then assessed. Among 684,388 PLR containers generated from the HMP dataset, only 11,579 were used to generate more than one PLR with different combinations of bubble nodes as described in the previous subsection. From the above 11,579 PLR containers, a total of 29,291 PLRs were generated (hereafter V-PLRs). Further evaluation was performed for them. For comparison, additional PLRs, called N-PLRs, were created from V-PLRs by placing ‘N’ at all positions corresponding to bubble nodes. To compare V-PLRs and N-PLRs, they were mapped to the known reference

genomes in the HMP dataset using minimap2 [35] with five different mismatch penalties (4, 6, 8, 10, and 12). Output alignments were filtered by mapping quality (≥ 20). Reference genome coverage was calculated and compared for both V-PLRs and N-PLRs using BEDTools [36]. In addition, the read depth distribution was calculated for both V-PLRs and N-PLRs and compared using output alignments generated with the smallest mismatch penalty which was 4.

Evaluation of PLRs based on metagenome assembly

For checking the usefulness of PLRs for metagenome assembly, four different assemblers, LINKS [37], metaSPAdes [20], OPERA-MS [19], and SSPACE-Longread [38], were used to generate metagenome assemblies for the HMP dataset. In this evaluation, two versions of assemblies, an initial assembly using only short reads in the HMP dataset and a PLR assembly using both short reads and PLRs, were generated and compared. In the case of metaSPAdes, the initial assembly was generated with default options and the PLR assembly was constructed with default options except for '--nanopore'. OPERA-MS was first performed with default options. A file of intermediately generated contigs from Megahit, an embedded module in OPERA-MS, was used for the initial assembly. Final contigs generated by OPERA-MS were used for the PLR assembly. In addition, using the initial assembly of metaSPAdes and OPERA-MS, additional long read scaffolding was carried out using LINKS [37] and SSPACE-Longread [38] with default options.

The quality of metagenome assemblies was assessed using various statistics, including the number of sequences, assembly contiguity, and the number of misassemblies, that were calculated by MetaQUAST [34] with default options after supplying the above known reference genomes present in the HMP dataset. For each known reference genome in the HMP dataset, alignments of the initial assembly and the PLR assembly, that were labeled "True" by MetaQUAST, were plotted using the Circlize R package [39].

Evaluation of PLRs based on metagenome assembly binning

Each assembly generated in the previous subsection was binned using MetaBAT2 (v 2.12.1) [40] with default options except for '--minContig 1500'. Using alignments between assemblies and the known reference genomes in the HMP dataset prepared with MetaQUAST as described in the previous subsection, a species label corresponding to the known reference genome was assigned to each bin. In this step, if the sequence of a bin is aligned to more than one reference genome, the reference genome with the largest alignment coverage was chosen.

Additionally, the completeness (the best: 100 and the worst: 0) and contamination (the best: 0 and the worse: no upper bound) of bins were measured based on the single-copy marker gene content calculated with the lineage workflow in CheckM (v.1.1.2) [41] using default options. Because each bin is labeled independently by CheckM, the same species can be assigned to multiple bins. To compare the quality of bins at the species level, a single representative bin for each species was chosen based on the completeness score as described in a recent study [19]. All bins were categorized into four classes, "Complete", "High-quality", "Moderate", and "Incomplete", based on their quality of completeness and contamination. Specifically, a bin with completeness $\geq$ 90 and 0 contamination was defined as "Complete". A bin with completeness $\geq$ 80 and contamination < 10 was defined as "High-quality". A bind with completeness $\geq$ 50 and contamination < 20 was defined as "Moderate". All other bins were defined as "Incomplete". Genes in each bin were also predicted with Prodigal (v2.6.3) [42] using default options to examine gene completeness (the best: 100; the worst: 0) of the bin. Gene completeness was calculated based on the fraction of completely predicted genes.

Results

PLRs provide valuable information for metagenome assembly in various aspects

Based on the predicted reference genomes and short paired-end reads (2 $\times$ 101 bp) in the HMP dataset (Methods), a total of 704,840 PLRs with a total length of 1,248 Mbp, N10 of 828 Kbp,

and N50 of 33 Kbp were obtained using PLR-GEN (Table 1 and Supplementary Table S4). Among them, 3,332 PLRs were longer than 50 Kbp (more than 500-fold longer than the input reads). Their total length was 501 Mbp. The maximum length of PLRs was 1.2 Mbp (more than 12,000-fold longer than the input reads). From the alignment of PLRs to the known reference genomes in the HMP dataset, 99.9 % of bases in PLRs were successfully aligned to 52 % of reference genome bases. The error rate was only 0.422 % (Methods).

In addition to its ability to elongate short reads with very low error rate as shown above, PLR-GEN can also distinguish genome fragments with only very small sequence variations, which can originate from individual genomes of the same or different species. PLR-GEN can generate multiple PLR sequences (hereafter called V-PLRs) with small sequence variations in one PLR container that represents such a genome fragment (Methods). In the evaluation with the HMP dataset, a total of 11,579 PLR containers generated 29,291 V-PLRs. They were then compared with 11,579 N-PLRs created by placing 'N' at all positions of variation in V-PLRs (Methods). V-PLRs and N-PLRs were mapped to the known reference genomes in the HMP dataset with various mismatch penalties, and V-PLRs could cover reference genomes more than 32 Kbp in average in comparison with N-PLRs (Supplementary Table S5). For example, in Fig. 2a, two V-PLRs created from the same PLR container having only three positions with different nucleotides were mapped to two different genomes of species, *S. agalactiae* and *S. mutans*, with a mismatch penalty 4. If a single N-PLR is generated by placing 'N' at the three positions with variation, it is possible that it cannot be mapped to the above two genomes without lowering the mismatch penalty. Depths of mapped V-PLRs and N-PLRs on all known reference genomes with the smallest mismatch penalty 4 were then compared (Methods; Fig. 2b). Whereas V-PLRs could be mapped with very high depth, N-PLRs failed to map to the known reference genome regions with a depth larger than 51x. These experiments clearly show that PLR-GEN can capture and use subtle sequence variations when generating PLRs to cover more

regions of reference genomes.

PLRs improve the quality of metagenome assembly when only short reads are available

PLRs created from metagenome short reads can be treated as general long reads and used in any assembly approaches relying on long reads. This approach is particularly useful when only short reads are available. Researchers can take advantages of long reads for metagenome assembly, which can be achieved by first generating PLRs from short reads by using PLR-GEN and then using PLRs for the metagenome assembly.

To examine whether PLRs can improve the quality of metagenome assembly, initial metagenome assemblies were constructed with metaSPAdes and OPERA-MS using short reads in the HMP dataset. They were further assembled to make PLR assemblies using PLRs generated by PLR-GEN (Methods). As shown in Fig. 3 and Supplementary Fig. S1, PLRs could (i) reduce the number of sequences (Fig. 3a and Supplementary Fig. S1a), (ii) increase assembly contiguity (Fig. 3b and 3c), and (iii) produce longer assembly sequences consistent with the known reference genomes in the HMP dataset (Fig. 3d) compared with both initial assemblies generated with metaSPAdes and OPERA-MS. For example, the number of sequences was reduced by 10 % when PLRs were used for the initial assembly with OPERA-MS (Fig. 3a). About two-fold increase of length was observed for long assembly sequences (Figs. 3b and 3c). In addition, in terms of NA50, the corrected N50 calculated after breaking the initial assembly at misaligned regions against the known reference genomes, was increased 44 % and 41 % using PLRs for initial assemblies with metaSPAdes and OPERA-MS, respectively (Fig. 3d). This trend was more prominent when additional long read-based scaffolding tools, LINKS and SSPACE-Longread, were used (Supplementary Fig. S1b and Table S6). These results clearly demonstrate that PLRs can play an important role in increasing the quality of metagenome assembly without relying on real long reads.

PLRs improve reconstructing microbial genomes and binning metagenome assembly

Metagenome assemblies can be used to reconstruct original microbial chromosome sequences, which can be further used for various downstream analyses, including metagenome assembly binning and gene prediction. To evaluate the effect of improved metagenome assemblies with PLRs for recovering original microbial chromosome sequences, the initial assembly and the PLR assembly generated with metaSPAdes were aligned against each of the known reference genomes in the HMP dataset (Methods). In most of those reference genomes, the PLR assembly could cover more contiguous regions (outer rings in Supplementary Fig. S2) than the initial assembly (inner rings in Supplementary Fig. S2). In the case of *S. mutans* (Fig. 4a), 99.56 % of its genome was covered by 21 alignment blocks created by the PLR assembly. However, 40 alignment blocks were used to cover similar genomic regions with the initial assembly. Specifically, the longest alignment block created by the PLR assembly was 524 Kbp, which was more than two-fold longer than the longest one (237 Kbp) created by the initial assembly. Similar pattern was observed in another reference species of *R. sphaeroides* (Fig. 4b). Specifically, chromosome 2 of *R. sphaeroides* was covered by only three alignment blocks of the PLR assembly whereas 14 alignment blocks of the initial assembly were needed to cover chromosome 2 of *R. sphaeroides*.

To examine the usefulness of PLR-assisted metagenome assemblies in other downstream analyses, both initial and PLR assemblies were binned, a species label was assigned to each bin, and the quality of each resulting bin was then evaluated for the assigned ten species (Methods). As shown in Fig. 4c and Supplementary Table S7, the PLR assembly increased the contiguity of binned sequences (bars in Fig. 4c) without sacrificing bin completeness or contamination (class symbols in Fig. 4c). In the case of gene completeness (numbers near each bar in Fig. 4c), the PLR assembly improved the quality of metagenome bins in comparison with initial assembly (from 95.91 % to 96.86 %; Fig. 4c and Supplementary Table S7). Specifically, for *R. sphaeroides* genome, N50 was increased more than five folds when the PLR

assembly was used. In terms of bin completeness and contamination, the PLR assembly was effective in improving the bin quality of *S. epidermidis* from “High-quality” to “Complete”. Additionally, the gene completeness was increased with PLRs for nine species-labeled bins. These findings indicate that the use of metagenome assemblies generated with PLRs from PLR-GEN is useful for downstream analyses.

Discussion

In this study, we presented a new method, called PLR-GEN, for generating pseudo-long reads (PLRs). PLRs are artificial long reads generated from next-generation sequencing short reads by utilizing microbial reference genomes. Our method was successfully applied to short reads of 101 bp in length in the HMP dataset by creating PLRs with N50 of 33 Kbp that could be almost completely aligned to the known reference genomes in the HMP dataset with very low error rate (Fig. 2 and Table 1). For metagenome assembly, initial assemblies created by metaSPAdes and OPERA-MS were further assembled using PLRs, leading to dramatic improvement of resulting assemblies in terms of the number of sequences and assembly contiguity (Fig. 3). Assemblies improved by PLRs were also very useful for assembly binning and reconstruction of species genome (Fig. 4). These improved species genomes resulted in increased completeness of gene prediction (Fig. 4 and Supplementary Tables S7).

Sequenced long reads can provide long-range information. They are very helpful for metagenome assembly. However, long sequence reads have high sequencing error rate and cost that prevent their widespread use for metagenome assembly. Therefore, short reads are still being for metagenome assembly and related studies [43-45]. In such situation, it is very helpful to generate long reads by just using short reads and reference genome sequences without needing extra sequencing cost. PLRs generated by our method can be used in many studies involving metagenomes, including assembly-based studies as shown here by treating them as

normal long reads such as PacBio [46] and Nanopore [47] reads.

One of excellent features of our method is its ability to capture subtle sequence variations resulting from individual genomes in the same or different species in a metagenome sample. This was achieved by (i) carefully identifying mapping positions of short reads occupied by multiple different nucleotides, (ii) representing them as vectors of sequence variations, and (iii) grouping them using a hierarchical clustering algorithm based on a newly designed distance measure. The effect of this feature was confirmed in comparison with PLRs generated by turning this feature off (Fig. 2). Therefore, PLRs generated by our method can also be used to discover information of haplotypes inherent in a metagenome [48].

Because PLR-GEN creates PLRs by relying on given microbial reference genomes, the quality of PLRs also depends on the number and quality of microbial reference genomes used. Another difficulty in metagenome assembly is that there are multiple unknown microorganisms in a metagenome sample sharing similar genomic regions with low sequence variations. In this situation, one important pre-processing step is to prepare the most appropriate reference genomes for a target metagenome sample. This can be achieved by using recently developed metagenome classifiers [33, 49-52] and collecting genomes of predicted species using those metagenome classifiers. To this end, TAMA [33], one of metagenome sequence classifiers, was used to prepare a set of reference genomes in the HMP dataset for making PLRs in this study. Another option is to use all microbial genomes in a public database such as NCBI, but it will take a lot of time and computer resources. However, continued accumulation of high-quality genome sequences of many microorganisms will make our method more valuable for studies involving metagenomes.

Availability of supporting source code and requirements

Project name: PLR-GEN

Project home page: https://github.com/jkimlab/PLR-GEN

Operating system: Linux

Programming language: Perl

Other requirements: Docker

License: MIT

Data Availability

The PLR-GEN package is available at: https://github.com/jkimlab/PLR-GEN.

Additional Files

Supplementary Table S1. NCBI accession numbers of the known reference genomes in the HMP dataset.

Supplementary Table S2. List of predicted reference genomes.

Supplementary Table S3. List of parameters of PLR-GEN used for evaluation.

Supplementary Table S4. N10 to N90 values of generated PLRs.

Supplementary Table S5. Comparison of reference coverage between V-PLRs and N-PLRs.

Supplementary Table S6. Statistics of short-read assemblies and assemblies improved by PLRs.

Supplementary Table S7. Quality of each bin of the metagenome assembly generated by metaSPAdes using only short reads.

Supplementary Fig. S1. metaSPAdes and OPERA-MS were separately used to create (i) the initial assembly only using short reads and (ii) the PLR assembly by further assembly with long read scaffolding tools using PLRs. These two types of assemblies were compared in terms of (a) the number of sequences and (b) NA50.

Supplementary Fig. S2. Circos plots illustrating alignments of initial and PLR assemblies for genomes of all species in the HMP dataset. Inner (green color) and outer (orange color) circles

represent initial and PLR assemblies, respectively.

List of abbreviations

PET: paired-end tag; PLRs: pseudo-long reads; HMP: Human Microbiome Project.

Ethics approval and consent to participate

Not applicable

Consent for publication

Not applicable

Competing interests

The author(s) declare no competing interests.

Funding

This paper was supported by Konkuk University Researcher Fund in 2021, a grant [NRF-2014M3C9A3063544] funded by the Ministry of Science and ICT of Korea, a grant [NRF-2019R1F1A1042018] funded by the Ministry of Education of Korea, and a grant [PJ01334302] funded by the Rural Development Administration of Korea.

Authors' contributions

JBK conceived and designed the study. JBK, MKS, JIL, and DHL designed the PLR-GEN algorithm. MKS implemented the pseudo-long read generation algorithm. MKS, SYW, and NYP performed experiments. MKS, SYW, NYP, DHK, and JBK interpreted the analysis results. MKS drafted the manuscript. JBK finalized the manuscript. All authors approved the final

manuscript.

Acknowledgements

Not applicable

References

1. Wang C, Li P, Yan Q, Chen L, Li T, Zhang W, et al. Characterization of the Pig Gut Microbiome and Antibiotic Resistome in Industrialized Feedlots in China. mSystems. 2019;4 6 doi:10.1128/mSystems.00206-19.
2. Almeida A, Mitchell AL, Boland M, Forster SC, Gloor GB, Tarkowska A, et al. A new genomic blueprint of the human gut microbiota. Nature. 2019;568 7753:499-504. doi:10.1038/s41586-019-0965-1.
3. Tully BJ, Graham ED and Heidelberg JF. The reconstruction of 2,631 draft metagenome-assembled genomes from the global oceans. Sci Data. 2018;5:170203. doi:10.1038/sdata.2017.203.
4. Wilkins LGE, Ettinger CL, Jospin G and Eisen JA. Metagenome-assembled genomes provide new insight into the microbial diversity of two thermal pools in Kamchatka, Russia. Sci Rep. 2019;9 1:3059. doi:10.1038/s41598-019-39576-6.
5. St John E, Flores GE, Meneghin J and Reysenbach AL. Deep-sea hydrothermal vent metagenome-assembled genomes provide insight into the phylum Nanoarchaeota. Environ Microbiol Rep. 2019;11 2:262-70. doi:10.1111/1758-2229.12740.
6. Bandla A, Pavagadhi S, Sridhar Sudarshan A, Poh MCH and Swarup S. 910 metagenome-assembled genomes from the phytobiomes of three urban-farmed leafy Asian greens. Sci Data. 2020;7 1:278. doi:10.1038/s41597-020-00617-9.
7. Nurk S, Meleshko D, Korobeynikov A and Pevzner PA. metaSPAdes: a new versatile metagenomic assembler. Genome research. 2017;27 5:824-34.
8. Li D, Liu C-M, Luo R, Sadakane K and Lam T-W. MEGAHIT: an ultra-fast single-node solution for large and complex metagenomics assembly via succinct de Bruijn graph. Bioinformatics. 2015;31 10:1674-6.
9. Namiki T, Hachiya T, Tanaka H and Sakakibara Y. MetaVelvet: an extension of Velvet assembler to de novo metagenome assembly from short sequence reads. Nucleic acids research. 2012;40 20:e155-e.
10. Boisvert S, Raymond F, Godzaridis E, Laviolette F and Corbeil J. Ray Meta: scalable de novo

metagenome assembly and profiling. Genome Biol. 2012;13 12:R122. doi:10.1186/gb-2012-13-12-r122.

11. Haider B, Ahn TH, Bushnell B, Chai J, Copeland A and Pan C. Omega: an overlap-graph de novo assembler for metagenomics. Bioinformatics. 2014;30 19:2717-22. doi:10.1093/bioinformatics/btu395.

12. Peng Y, Leung HC, Yiu SM and Chin FY. IDBA-UD: a de novo assembler for single-cell and metagenomic sequencing data with highly uneven depth. Bioinformatics. 2012;28 11:1420-8. doi:10.1093/bioinformatics/bts174.

13. Ayling M, Clark MD and Leggett RM. New approaches for metagenome assembly with short reads. Brief Bioinform. 2020;21 2:584-94. doi:10.1093/bib/bbz020.

14. Olson ND, Treangen TJ, Hill CM, Cepeda-Espinoza V, Ghurye J, Koren S, et al. Metagenomic assembly through the lens of validation: recent advances in assessing and improving the quality of genomes assembled from metagenomes. Brief Bioinform. 2019;20 4:1140-50. doi:10.1093/bib/bbx098.

15. Kuleshov V, Jiang C, Zhou W, Jahanbani F, Batzoglou S and Snyder M. Synthetic long-read sequencing reveals intraspecies diversity in the human microbiome. Nat Biotechnol. 2016;34 1:64-9. doi:10.1038/nbt.3416.

16. Xie H, Yang C, Sun Y, Igarashi Y, Jin T and Luo F. PacBio Long Reads Improve Metagenomic Assemblies, Gene Catalogs, and Genome Binning. Front Genet. 2020;11:516269. doi:10.3389/fgene.2020.516269.

17. Rhoads A and Au KF. PacBio Sequencing and Its Applications. Genomics Proteomics Bioinformatics. 2015;13 5:278-89. doi:10.1016/j.gpb.2015.08.002.

18. Moss EL, Maghini DG and Bhatt AS. Complete, closed bacterial genomes from microbiomes using nanopore sequencing. Nat Biotechnol. 2020;38 6:701-7. doi:10.1038/s41587-020-0422-6.

19. Bertrand D, Shaw J, Kalathiyappan M, Ng AHQ, Kumar MS, Li C, et al. Hybrid metagenomic assembly enables high-resolution analysis of resistance determinants and mobile elements in human microbiomes. Nat Biotechnol. 2019;37 8:937-44. doi:10.1038/s41587-019-0191-2.

20. Antipov D, Korobeynikov A, McLean JS and Pevzner PA. hybridSPAdes: an algorithm for hybrid assembly of short and long reads. Bioinformatics. 2016;32 7:1009-15. doi:10.1093/bioinformatics/btv688.

21. Damashek J, Edwardson CF, Tolar BB, Gifford SM, Moran MA and Hollibaugh JT. Coastal Ocean Metagenomes and Curated Metagenome-Assembled Genomes from Marsh Landing, Sapelo Island (Georgia, USA). Microbiol Resour Announc. 2019;8 40 doi:10.1128/MRA.00934-19.

22. Wilkinson T, Korir D, Ogugo M, Stewart RD, Watson M, Paxton E, et al. 1200 high-quality metagenome-assembled genomes from the rumen of African cattle and their relevance in the context of sub-optimal feeding. Genome Biol. 2020;21 1:229. doi:10.1186/s13059-020-02144-7.

23. Glendinning L, Stewart RD, Pallen MJ, Watson KA and Watson M. Assembly of hundreds of novel bacterial genomes from the chicken caecum. Genome Biol. 2020;21 1:34. doi:10.1186/s13059-020-1947-1.

24. Marques M, Borges N, Silva SG, da Rocha UN, Lago-Lestón A, Keller-Costa T, et al. Metagenome-Assembled Genome Sequences of Three Uncultured Planktomarina sp. Strains from the Northeast Atlantic Ocean. Microbiol Resour Announc. 2020;9 12 doi:10.1128/MRA.00127-20.

25. Vandervalk BP, Yang C, Xue Z, Raghavan K, Chu J, Mohamadi H, et al. Konnector v2.0: pseudo-long reads from paired-end sequencing data. BMC Med Genomics. 2015;8 Suppl 3:S1. doi:10.1186/1755-8794-8-S3-S1.

26. Hammond SA, Warren RL, Vandervalk BP, Kucuk E, Khan H, Gibb EA, et al. The North American bullfrog draft genome provides insight into hormonal regulation of long noncoding RNA. Nat Commun. 2017;8 1:1433. doi:10.1038/s41467-017-01316-7.

27. Nicholls SM, Aubrey W, De Grave K, Schietgat L, Creevey CJ and Clare A. On the complexity of haplotyping a microbial community. Bioinformatics. 2020;37 10:1360–6. doi:10.1093/bioinformatics/btaa977.

28. Consortium HMP. A framework for human microbiome research. Nature. 2012;486 7402:215-21. doi:10.1038/nature11209.

29. Consortium HMP. Structure, function and diversity of the healthy human microbiome. Nature. 2012;486 7402:207-14. doi:10.1038/nature11234.

30. Langmead B and Salzberg SL. Fast gapped-read alignment with Bowtie 2. Nature methods. 2012;9 4:357.

31. Danecek P, Bonfield JK, Liddle J, Marshall J, Ohan V, Pollard MO, et al. Twelve years of SAMtools and BCFtools. Gigascience. 2021;10 2 doi:10.1093/gigascience/giab008.

32. Zepeda-Mendoza ML and Resendis-Antonio O. Hierarchical Agglomerative Clustering. In: Dubitzky W, Wolkenhauer O, Cho K-H and Yokota H, editors. Encyclopedia of Systems Biology. New York, NY: Springer New York; 2013. p. 886-7.

33. Sim M, Lee J, Lee D, Kwon D and Kim J. TAMA: improved metagenomic sequence classification through meta-analysis. BMC Bioinformatics. 2020;21 1:185. doi:10.1186/s12859-020-3533-7.

34. Mikheenko A, Saveliev V and Gurevich A. MetaQUAST: evaluation of metagenome assemblies. Bioinformatics. 2016;32 7:1088-90. doi:10.1093/bioinformatics/btv697.

35. Li H. Minimap2: pairwise alignment for nucleotide sequences. Bioinformatics. 2018;34 18:3094-100. doi:10.1093/bioinformatics/bty191.

36. Quinlan AR and Hall IM. BEDTools: a flexible suite of utilities for comparing genomic features. Bioinformatics. 2010;26 6:841-2.

37. Warren RL, Yang C, Vandervalk BP, Behsaz B, Lagman A, Jones SJ, et al. LINKS: Scalable, alignment-free scaffolding of draft genomes with long reads. Gigascience. 2015;4:35. doi:10.1186/s13742-015-0076-3.

38. Boetzer M and Pirovano W. SSPACE-LongRead: scaffolding bacterial draft genomes using long read sequence information. BMC Bioinformatics. 2014;15:211. doi:10.1186/1471-2105-15-211.

39. Gu Z, Gu L, Eils R, Schlesner M and Brors B. circlize Implements and enhances circular visualization in R. Bioinformatics. 2014;30 19:2811-2. doi:10.1093/bioinformatics/btu393.

40. Kang DD, Li F, Kirton E, Thomas A, Egan R, An H, et al. MetaBAT 2: an adaptive binning algorithm for robust and efficient genome reconstruction from metagenome assemblies. PeerJ. 2019;7:e7359. doi:10.7717/peerj.7359.

41. Parks DH, Imelfort M, Skennerton CT, Hugenholtz P and Tyson GW. CheckM: assessing the quality of microbial genomes recovered from isolates, single cells, and metagenomes. Genome Res. 2015;25 7:1043-55. doi:10.1101/gr.186072.114.

42. Hyatt D, Chen GL, Locascio PF, Land ML, Larimer FW and Hauser LJ. Prodigal: prokaryotic gene recognition and translation initiation site identification. BMC Bioinformatics. 2010;11:119. doi:10.1186/1471-2105-11-119.

43. Zhou S, Luo R, Gong G, Wang Y, Gesang Z, Wang K, et al. Characterization of Metagenome-Assembled Genomes and Carbohydrate-Degrading Genes in the Gut Microbiota of Tibetan Pig. Front Microbiol. 2020;11:595066. doi:10.3389/fmicb.2020.595066.

44. Chen H, Liu C, Teng Y, Zhang Z, Chen Y and Yang Y. Environmental risk characterization and ecological process determination of bacterial antibiotic resistome in lake sediments. Environ Int. 2020;147:106345. doi:10.1016/j.envint.2020.106345.

45. Youngblut ND, de la Cuesta-Zuluaga J, Reischer GH, Dauser S, Schuster N, Walzer C, et al. Large-Scale Metagenome Assembly Reveals Novel Animal-Associated Microbial Genomes, Biosynthetic Gene Clusters, and Other Genetic Diversity. mSystems. 2020;5 6 doi:10.1128/mSystems.01045-20.

46. Eid J, Fehr A, Gray J, Luong K, Lyle J, Otto G, et al. Real-time DNA sequencing from single polymerase molecules. Science. 2009;323 5910:133-8. doi:10.1126/science.1162986.

47. Jain M, Koren S, Miga KH, Quick J, Rand AC, Sasani TA, et al. Nanopore sequencing and assembly of a human genome with ultra-long reads. Nat Biotechnol. 2018;36 4:338-45. doi:10.1038/nbt.4060.

48. Nicholls SM, Aubrey W, De Grave K, Schietgat L, Creevey CJ and Clare A. On the complexity of haplotyping a microbial community. Bioinformatics. 2020; doi:10.1093/bioinformatics/btaa977.

49. Wood DE, Lu J and Langmead B. Improved metagenomic analysis with Kraken 2. Genome Biol. 2019;20 1:257. doi:10.1186/s13059-019-1891-0.

50. Lu J, Breitwieser FP, Thielen P and Salzberg SL. Bracken: estimating species abundance in metagenomics data. PeerJ Computer Science. 2017;3:e104.

51. Milanese A, Mende DR, Paoli L, Salazar G, Ruscheweyh HJ, Cuenca M, et al. Microbial abundance, activity and population genomic profiling with mOTUs2. Nat Commun. 2019;10 1:1014. doi:10.1038/s41467-019-08844-4.

52. Corvelo A, Clarke WE, Robine N and Zody MC. taxMaps: comprehensive and highly accurate taxonomic classification of short-read data in reasonable time. Genome Res. 2018;28 5:751-8. doi:10.1101/gr.225276.117.

Figure Legends

Figure 1. Workflow of pseudo-long read generation (PLR-GEN). Using next-generation sequencing (NGS) short reads of a metagenome sample and reference genome sequences as input, NGS reads are mapped to reference genome sequences. Mapped reads are pilled-up on aligned reference genome positions (the mapping & piling-up step). Reference genome regions with continuously mapped reads are defined as PLR containers (the PLR container construction step). The PLR graph is constructed using two types of nodes, normal nodes (N1 to N5) and bubble nodes (nodes corresponding to B1 to B4), and directed edges representing the order of nodes in the PLR container (the PLR graph generation step). Vectors of bubble nodes are created and grouped by the hierarchical clustering algorithm to identify various combinations of bubble nodes (the bubble combination identification step). Together with normal nodes flanking bubble nodes, each of different bubble node combinations is converted to a single PLR path (the PLR path identification step). Finally, an actual PLR sequence is constructed by concatenating nucleotides in normal and bubble nodes in each PLR path (the PLR sequence generation step).

Figure 2. Comparison of two types of PLRs generated from the mock community dataset in the Human Microbiome Project. (a) Two different V-PLRs, V-PLR1 and V-PLR2, generated from the same PLR container but with small sequence variations are mapped to two different reference genomes. (b) After mapping all V-PLRs and N-PLRs to reference genomes, their read depth distributions are plotted.

Figure 3. Comparison of assembly statistics generated by different assemblers for initial and PLR assemblies using the mock community dataset in the Human Microbiome Project. metaSPAdes and OPERA-MS were separately used to create (i) the initial assembly

only using short reads and (ii) the PLR assembly by further assembling it using PLRs. These two types of assemblies were compared in terms of (a) the number of sequences, (b, c) assembly contiguity, and (d) NA50.

Figure 4. Comparison of assemblies generated by metaSPAdes using the mock community dataset in the Human Microbiome Project in terms of (a, b) species genome reconstruction and (c) assembly sequence binning. Initial and PLR assemblies were aligned and visualized using the Circlize R package[39] for *S. mutans* (a) and *R. sphaeroides* (b) genomes. (c) After binning assembly sequences and finding representative bins for ten species, their quality was measured in terms of N50 (bars), levels of completeness and contamination (different shapes), and gene completeness (numbers near bars).

Tables

Table 1. Statistics of pseudo-long reads generated from short paired-end reads (2 × 101 bp) using the mock community dataset in the Human Microbiome Project

No. of sequence (sequence in length > 50 Kbp)	704,840 (3,332)
Total length (sequence in length > 50 Kbp)	1,248,362,272 (501,383,852)
Min	100
Max	1,241,545
N50	32,996
Total aligned length (% of aligned bases)[1]	1,247,305,552 (99.915 %)
Genome fraction[2]	52.017 %
Error rate[3]	0.422 %

[1]Total length of aligned PLR bases to the known reference genomes in the mock community dataset.

[2]Coverage of the known reference genomes in the mock community dataset by aligned PLRs.

[3]The proportion of PLRs assigned as ‘misassembled contigs’ by MetaQUAST.

Figure1

Click here to access/download;Figure;Figure 1.pdf

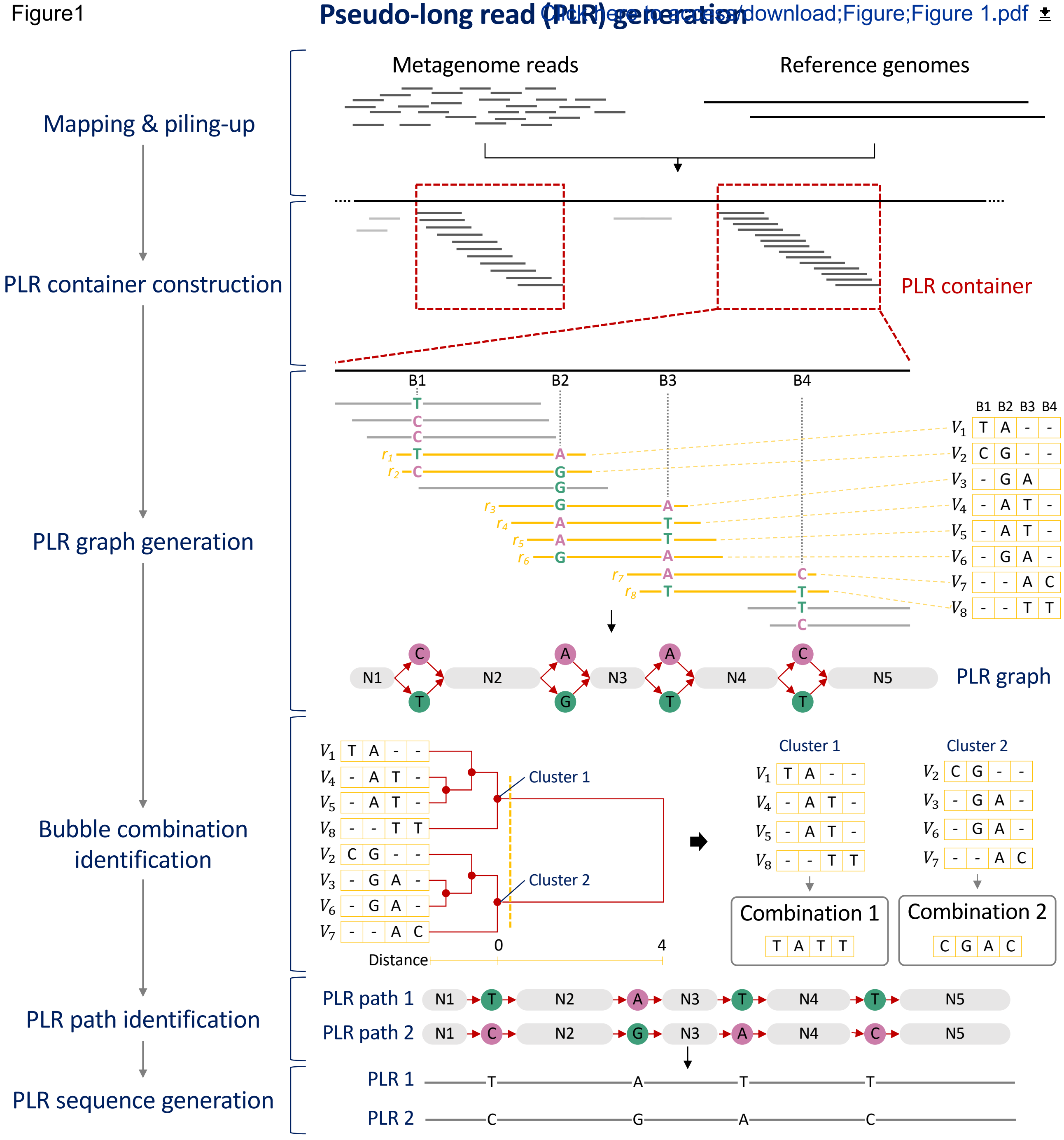

Figure2

Click here to access/download;Figure;Figure 2.pdf

a

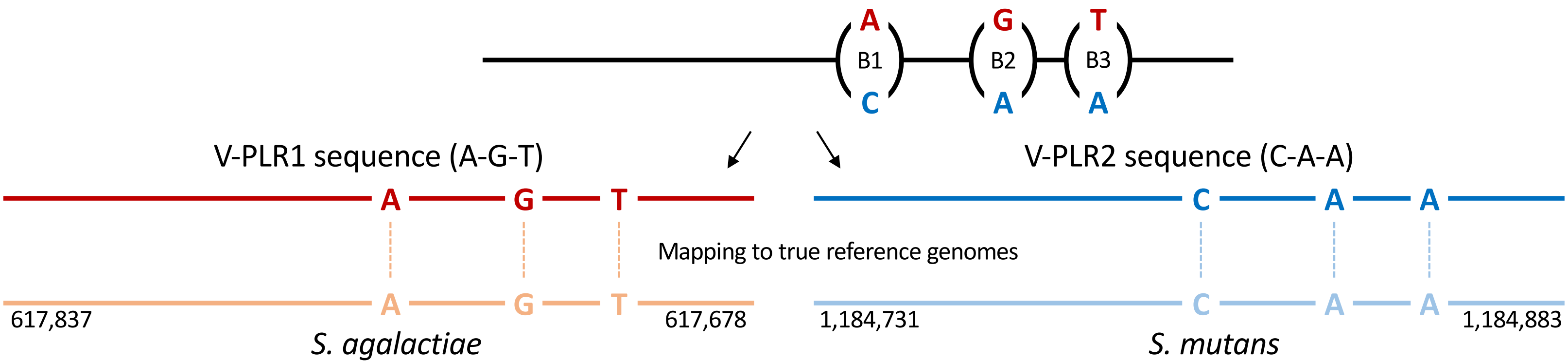

b

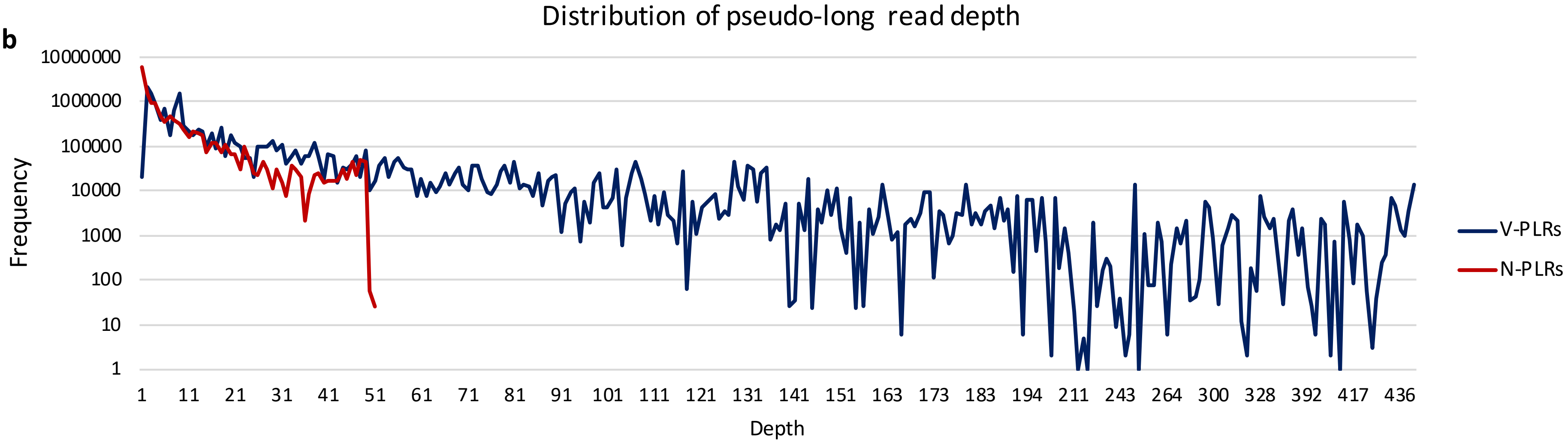

Figure3

Click here to access/download;Figure;Figure 3.pdf

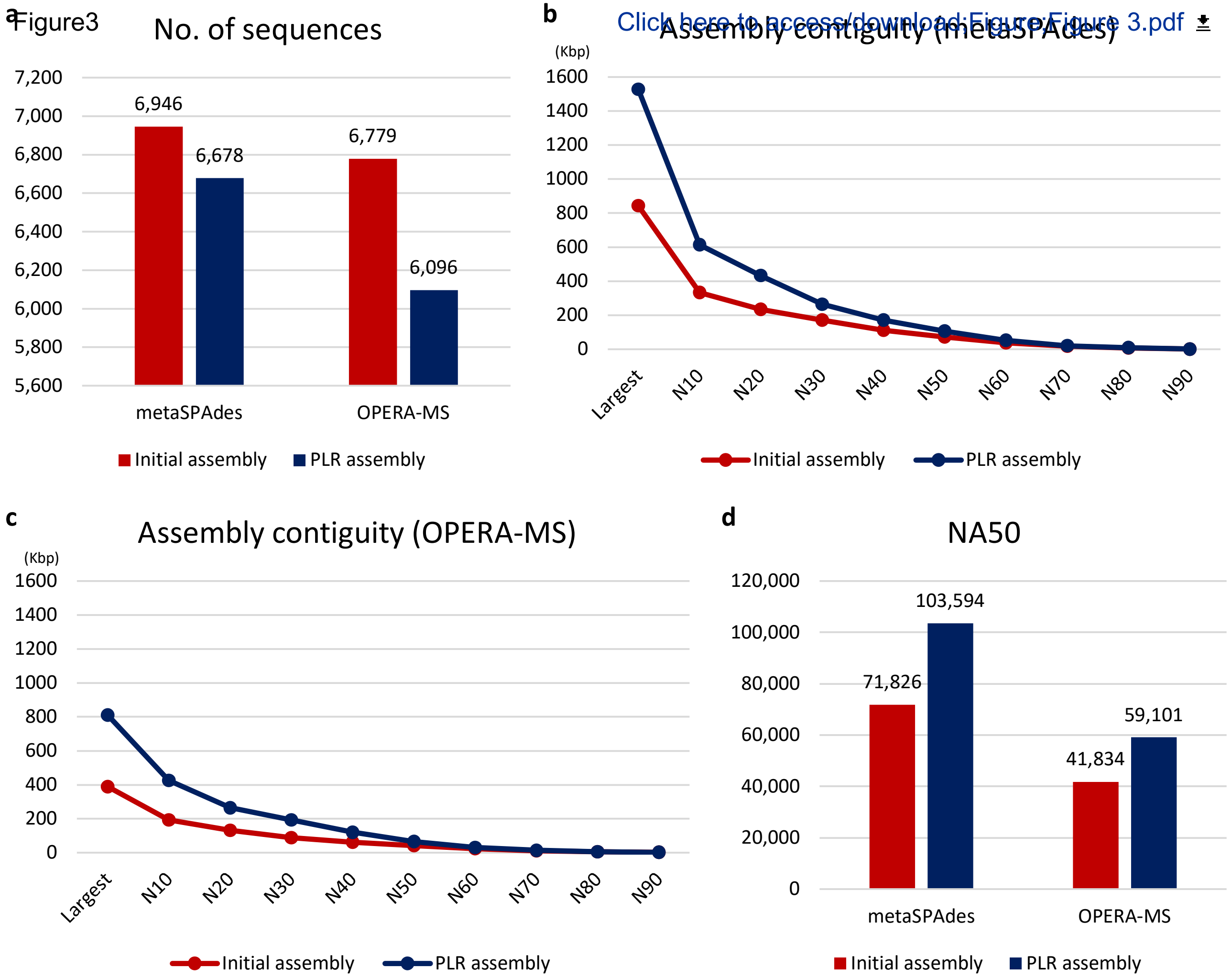

Click here to access/download;Figure;Figure 4.pdf

Figure4

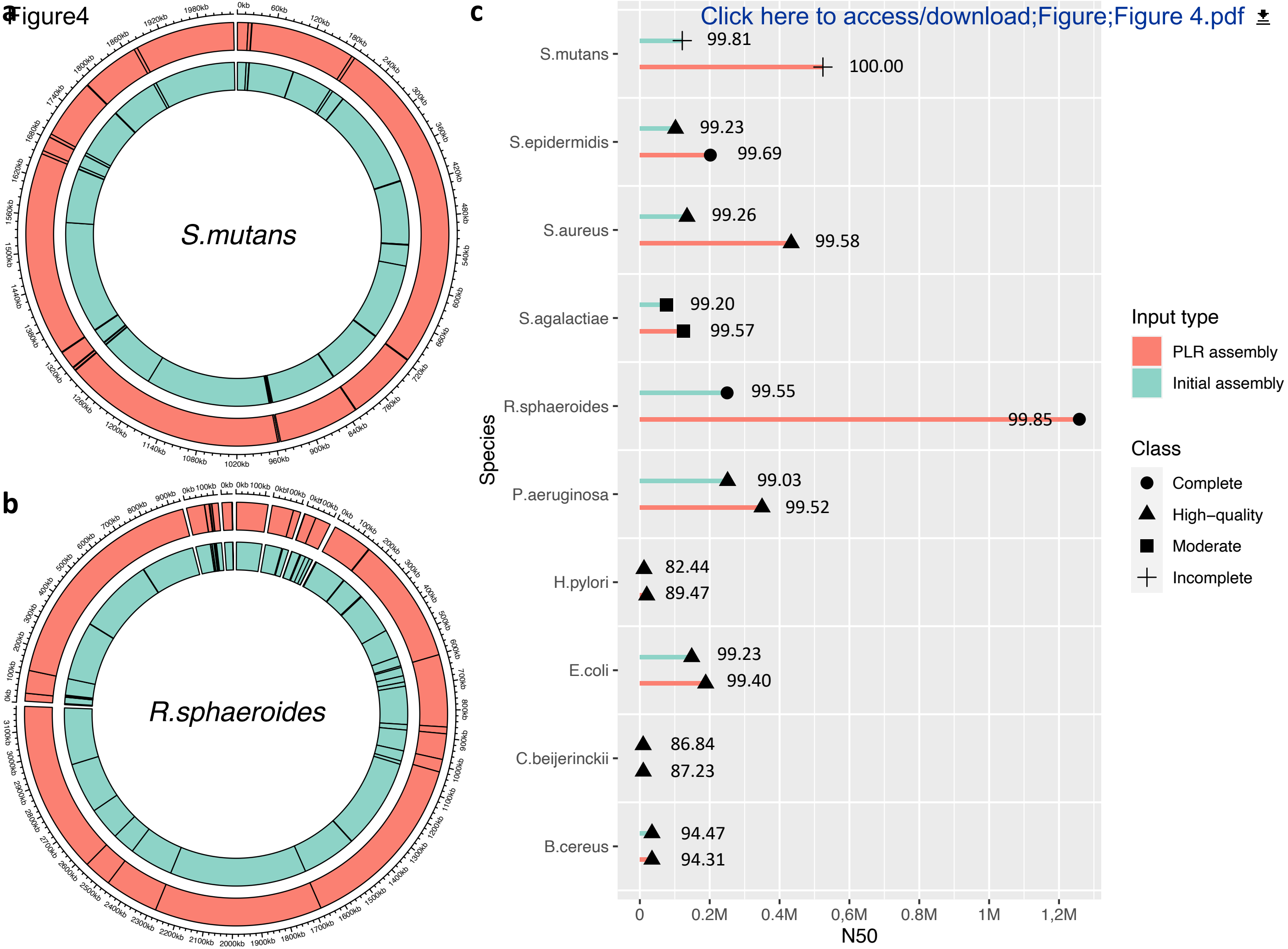

Supplementary Figures

Click here to access/download
Supplementary Material
Supplementary_figures.docx

Supplementary data

Click here to access/download
Supplementary Material
Supplementary_data.xlsx
