# Supplementary material for: Generation and application of pseudo–long reads for metagenome assembly
Source: Gigascience. 2022 May 17;11:giac044. doi: 10.1093/gigascience/giac044 (PMC9112764; doi:10.1093/gigascience/giac044)
Supplement: giac044_GIGA-D-21-00349_Revision_1 [file giac044_giga-d-21-00349_revision_1.pdf]

GigaScience

Generation and application of pseudo-long reads for metagenome assembly

--Manuscript Draft--

**Manuscript Number:**	GIGA-D-21-00349R1
**Full Title:**	Generation and application of pseudo-long reads for metagenome assembly
**Article Type:**	Technical Note
**Funding Information:**	the Ministry of Education of Korea (NRF-2021M3H9A2097134) — Dr Jaebum Kim<br>Konkuk University Researcher Fund in 2021 — Dr Jaebum Kim<br>the Ministry of Science and ICT of Korea (NRF-2014M3C9A3063544) — Dr Jaebum Kim<br>the Ministry of Education of Korea (NRF-2019R1F1A1042018) — Dr Jaebum Kim<br>the Rural Development Administration of Korea (PJ01334302) — Dr Jaebum Kim
**Abstract:**	Background<br><br>Metagenomic assembly using high-throughput sequencing data is a powerful method to construct microbial genomes in environmental samples without cultivation. However, metagenomic assembly, especially when only short reads are available, is a complex and challenging task because mixed genomes of multiple microorganisms constitute the metagenome. Although long read sequencing technologies have been developed and have begun to be used for metagenomic assembly, many metagenomic studies have been performed based on short reads because the generation of long reads requires higher sequencing cost than short reads.<br><br>Results<br><br>In this study, we present a new method called PLR-GEN. It creates pseudo-long reads from metagenomic short reads based on given reference genome sequences by considering small sequence variations existing in individual genomes of the same or different species. When applied to a mock community dataset in the Human Microbiome Project, PLR-GEN dramatically extended short reads in length of 101 bp to pseudo-long reads with N50 of 33 Kbp and 0.4% error rate. The use of these pseudo-long reads generated by PLR-GEN resulted in an obvious improvement of metagenomic assembly in terms of the number of sequences, assembly contiguity, and prediction of species and genes.<br><br>Conclusions<br><br>PLR-GEN can be used to generate artificial long read sequences without spending extra sequencing cost, thus aiding various studies using metagenomes.
**Corresponding Author:**	Jaebum Kim<br>Konkuk University<br>Seoul, KOREA, REPUBLIC OF
**Corresponding Author Secondary Information:**	
**Corresponding Author's Institution:**	Konkuk University
**Corresponding Author's Secondary Institution:**	
**First Author:**	Mikang Sim
**First Author Secondary Information:**	
**Order of Authors:**	Mikang Sim

Powered by Editorial Manager® and ProduXion Manager® from Aries Systems Corporation

	Jongin Lee
	Suyeon Wy
	Nayoung Park
	Daehwan Lee
	Daehong Kwon
	Jaebum Kim
**Order of Authors Secondary Information:**	
**Response to Reviewers:**	Response to Reviewers<br><br>Reviewer #1<br><br>Authors present a new reference-guided local assembly method to be applied for metagenome analysis and microbial species identification. Although pseudo long reads built from short-reads have been used to the assembly problem of a single genome, no successful application for the meta-genomes.<br>Overall, the PLR_GEN method outperformed a simple assembly result in terms of the number of assembled sequences, the quality of the resulting assembly, and identification of species and genes.<br><br>However, i am not quite convinced why they have to build pseudo long reads to identify species and genes using the reference genomes. If there are reference genomes, they could simply map the short reads to the genomes rather making pseudo long reads. They have to check whether the approach to use pseudo long reads are better than alternative way to map the short reads in terms of the quality of assembly and idenification of species and genes.<br><br>Response:<br>We highly appreciate the reviewer’s comment and apologize for the confusion. The main purpose of generating PLRs is not just for the identification of species and genes. It was one of the experiments to show the usefulness of our PLRs. As you know, the reference genomes collected from NCBI only contain the genomes of known species. On the other hand, most of the species in a metagenomic sample are unknown, and therefore their genomes do not exist in the reference genomes. However, even though the complete genome of an unknown species does not exist in the reference genomes, it is highly probable that some portions of its genome exist in the reference genomes because different species can share highly similar genomic regions and it can happen between the known species in the reference genomes and the unknown species in a metagenomic sample. Therefore, the generated PLRs using the reference genomes can be parts of the genomes of unknown species in a metagenomic sample, and the PLRs can be used very effectively to construct accurate metagenomic assembly for the sample. This is the main motivation of our study.<br><br>However, because most of the species in a metagenomic sample are unknown, it is not easy to evaluate the generated PLRs and the assembly sequences constructed by using the PLRs. To address this problem, we chose the mock community dataset in the Human Microbiome Project, which is one of the most widely used dataset with complete species information in it for metagenomic studies, and showed the clear usefulness of our approach by performing various experiments including the identification of species and genes. Therefore, we are sure that our approach can contribute greatly to improve the quality of metagenomic assemblies.<br><br><br>Reviewer #2<br><br>General Comments:<br><br>The authors have presented a tool for generating pseudo-long-reads (PLR) from short-read data. The method relies on the availability of a sufficient number of relevant reference genomes. The authors demonstrate on a single, well characterised community that their approach produces generalised improvement in assembly quality

Powered by Editorial Manager® and ProduXion Manager® from Aries Systems Corporation

metrics, which subsequently led to improvements in genome binning quality metrics.

This reviewer found their approach interesting and the algorithm description easy to comprehend. Although not discussed by the authors, this work recalls other analytical methods within metagenomics that attempt to encompass microbial variation.

The writing quality is of an acceptable level for communicating the authors findings, but any efforts to improve it further would benefit the reader.

Specific issues:
- The database they include in the repo with the 52 reference genomes is tailor-made to their test dataset. Granted its not impossible to do a naïve assembly, identify the taxa present, expand the reference genome database, and run this method, but something that integral to the method should be done by the tool, not manually.

Response:
We highly appreciate this comment and agree with the reviewer. In this revision, we added a new option (-tama) in PLR-GEN for automatically preparing reference genomes tailored to user's data by using TAMA, and updated the description in the Github page.

- Error rates between assemblies with and without PLR's, are not provided, only the error rate of the PLR sequences.

Response:
We highly appreciate this comment. We added the error rates for all assemblies with and without PLRs in Supplementary Table S5. In this revision, we added a new comparison with the PLRs generated by IDBA-UD. Therefore, the results of new comparison are also added in Table 1 and Supplementary Table S5.

Here we note that the error rates in Table 1 is more appropriate to compare PLR-GEN and IDBA-UD than the ones in Supplementary Table S5. It is because the error rates in Table 1 were obtained directly comparing the PLRs with true answers. However, the error rates in Supplementary Table S5 were calculated by comparing the assemblies generated by other scaffolding programs. Therefore, those numbers in Supplementary Table S5 can contain errors caused by the scaffolding programs (not completely by the used PLRs).

- See Figure 1 for an example of a paper that compares their approach to existing tools in the field: https://www.nature.com/articles/s41587-020-00777-4 . This paper lacks any external comparisons apart from running assemblers with and without their PLRs. This is useful, but needs to be assessed in context of current approaches, and in the context of error rates. See also this table 1 from another work: https://academic.oup.com/view-large/206266243 . Tools like IDBA-UD already do local assembly which could be considered PLR's. I'd want to see how they compare.

Response:
We highly appreciate this comment and agree with the reviewer. In this revision, we added the PLRs generated by IDBA-UD and compared them with ours in terms of the quality of raw sequences (Table 1) and the quality of assemblies generated by using them (Figure 3 and Supplementary Table S5).

We found that our PLRs were better than the ones created by IDBA-UD in terms of contiguity, coverage, and error rate (Table 1). In the comparison of assemblies generated by the PLRs, the assemblies generated by using our PLRs were better than the ones generated by using PLRs of IDBA-UD in terms of the number of sequences, contiguity, and coverage (Figure 3 and Supplementary Table S5). In addition, in all cases, NA50 of our results, which is the corrected N50 calculated after breaking sequences at misassembled positions, is better than NA50 of the results of IDBA-UD. NA50 is a kind of measure calculated by considering both contiguity and the amount of

misassemblies.

We added description for the results of this additional comparison in our manuscript (Lines 212-215, 244, 248, 289-291, and 334-338).

- The authors discuss the high sequencing error rate of long reads, but recently the ONT folks have started to claim their assemblies are not improved by the addition of short reads. So long reads might be more expensive, but the error issue is becoming controversial at worst or null at best.

Response:
We highly appreciate this comment and agree with the reviewer. We know that there is a recent dramatic improvement of the quality of long reads, such as the development of HiFi reads. We mentioned the error rate of long reads because such high-quality long reads are not used widely in metagenomic assembly yet. But we agree that it is a controversial issue. Therefore, we removed the parts related with the error rates (Lines 34, 67-68, and 384).

- The authors claim that their method can capture "subtle sequence variations resulting from individual genomes in the same or difference species". While I don't doubt this to be true, it needs to be demonstrated for this dataset. Moreover, a more fair comparison would be to assemble against a database not derived from the test data.

Response:
We highly appreciate this comment and apologize for not accurately describing the results. We think it is already demonstrated in Figure 2. In Figure 2a, two PLRs with only three sequence variations were generated from the same PLR container, and they were successfully mapped to two different genomes of different species separately. In Figure 2b, we examined all V-PLRs (considering subtle sequence variations) and found that they can be mapped more widely than N-PLRs (not considering subtle sequence variations).

About the fair comparison, we think our original description is not good enough and confused the reviewer. Even though we used the short reads contained in the HMP dataset, the generation of PLRs was done without using any information of the known reference genomes in the HMP dataset. The reference genomes used for the PLR generation were extracted from the genomes in the NCBI database by a program called TAMA. Therefore, we think we can say that it is a fair comparison. We added description to clarify the way of data generation and comparison (Lines 201-203, and 224-225).

- The algorithmic approaches presented here are fascinating, and I would need more time to ingest. It seems very promising, and I love the idea of using hierarchical clustering to parse out taxonomic differences in the present of SNPs, but again I would need to see it in context.

Response:
We highly appreciate this positive comment. We will make our approach contribute more for better understanding metagenomes.

Specific Code/GitHub comments:
- I thank the authors for making their code available
- vendoring many different tools (see https://gist.github.com/datagrok/8577287 why vendoring is bad)

Response:

We highly appreciate this comment and the link of a good post about why vendoring is bad. Although our package includes several specific versions of third-party programs, they are stored as a whole as compressed files and can be downloaded at the time of installation. We also provide a separate code for their installation to help users for using our tool. We think this may be the best approach at this point for making our tool be used easily. But we will definitely figure out a better way when we update our tool.

And there were binary executable files in the “bin” directory in the original Github repository. They were accidentally added even though they are not necessary to be downloaded. In this revision, they were deleted completely.

- I was not able to get their docker container to run; I’m sure its possible, but their executable isn’t used by the CMD or RUN directive, nor is it in the PATH of the container

Response:
We highly appreciate this comment and apologize for this problem. In this revision, we added more explanation for running PLR-GEN with docker in the Github page, and added PATH for all executable codes in our docker image. In addition, our docker image was updated with the new option for preparing reference genomes using TAMA, and the docker repository was changed from mksim/plrgen to jkimlab/plrgen.

- tool isn't installable via conda - A conda recipe would be welcome, if all the authors were to do is document preparing an environment or better publishing an environment definition (yaml) within which all the dependencies are satisfied short of PLR-GEN itself.

Response:
We highly appreciate this comment and agree with the reviewer. In this revision, we created the conda environment definition file (plrgen-env.yml) with all the dependencies for running PLR-GEN, and added the description in the Github page.

- I'm not a Perl expert, but I can usually read enough to tell what code is doing. This code is uncommented, undocumented (apart from the README), and inscrutable to me. Recommend the authors read
https://journals.plos.org/ploscompbiol/article?id=10.1371/journal.pcbi.1006561

Response:
We highly appreciate this comment. In this revision, we added more comments within the code.

- I couldn't get the tool to run, but looking at the code and reference database I am concerned with speed. The included bespoke set of reference genomes would likely need to be expanded for real-world use, and that could add significant runtime if not approached carefully.

Response:
We highly appreciate this comment and definitely agree with the reviewer. The number of reference genomes is very important because it affects the quality of the generated PLRs and runtime. And it is not easy to prepare the most appropriate reference genomes tailored to sequencing data that users want to analyze. Therefore, in this revision, we added a new option (-tama) for automatically preparing reference genomes tailored to users’ data by using TAMA to address the above problems. In addition, if too many genomes are included in the created reference genome dataset, it is possible that the runtime of PLR-GEN becomes quite long. For handling this situation, we created a new option of PLR-GEN (-sampling), that can be used to

randomly sample a given proportion of genomes from the prepared reference genome dataset. By using this option, users can reduce the runtime. Of course, if the amount of reference genomes is reduced, it affects the final quality of PLRs. However, based on our preliminary experiments, the small number of reference genomes does have an effect on improving the quality of assemblies (Supplementary Table S8). These results are also discussed in the Discussion section (Lines 404-407).

Major remarks:

* Line 71: The statement made here – that artificial long-reads are advantageous – has not yet been touched on by the authors, nor supported with external citations. As such, I feel this should be restated in less absolute terms or discussion which successfully makes the case should precede it.

Response:
We highly appreciate this comment and agree with the reviewer. In this revision, we restated the statement (Lines 71-73) as follows.

From:
In this situation, the generation and use of artificial long reads from real short reads can be a good alternative to take advantage of benefits of both short and long reads without needing extra sequencing cost.

To:
In this situation, there was a recent effort to generate and use artificial long reads from real short reads to take advantage of the benefits of both short and long reads without needing extra sequencing cost.

* During container construction, it would seem that breaking reference genomes only at the point there is zero coverage is quite lenient. Particularly because I do not see a constraint on the mapping of reads beyond quality q=>20, although I must admit to a lack of familiarity with default bowtie options. Are the authors not concerned about partial alignments that will be more prevalent within regions of genomic plasticity.

Response:
We highly appreciate this comment and apologize for not accurately describing our method. When constructing the PLR container, we also used additional filtering option of SAMtools other than q>=20. It was presented as "--ff UNMAP,QCFAIL,DUP,SECONDARY" without any description. The meaning of the option is to discard unmapped and duplicated reads as well as secondary alignments. We believe that the situation mentioned by the reviewer is alleviated because of this additional filtering option. In this revision, we added the description of the option in our manuscript (Lines 107-109).

* How many references are required for PLR-GEN to be considered reliable? This would make it clear to the read if PLR-GEN could be applied to their project.

Response:
We highly appreciate this comment. Choosing the most appropriate number of reference genomes is not an easy task. But to check its effect empirically, we performed additional experiments by generating six more sets of PLRs using different number of reference genomes (10, 50, 100, 150, 300, and 500), which were created by random sampling from the TAMA predicted set of reference genomes, and the quality of generated assemblies by using them were compared (Supplementary Table S8). Here we found that even with only 10 reference genomes, there was an improvement of the generated assembly compared with the original SR assembly. We discussed this result in the Discussion section (Lines 404-407).

Powered by Editorial Manager® and ProduXion Manager® from Aries Systems Corporation

	However, we know that this experimental result is not enough for making the most appropriate number of reference genomes. As mentioned in other comment above, we added a new option that can prepare the most appropriate reference genomes by using TAMA and genomes in the NCBI database, which can be also reduced by using the new random sampling option in PLR-GEN. We believe that these additional features will help users to address this issue to some degree.<br><br>* It would have been interesting to compare the PLR-GEN performance for the limited TAMA predicted reference set and the complete HMP set. I would imagine users will opt for what delivers the greatest performance, so long as it problem remains computationally feasible.<br><br>Response:<br>We highly appreciate this comment. As mentioned in the previous comment, additional experiments with various small numbers of reference genomes were performed, and their results were included in this revision. We hope this can be used as useful information for users. We note that the TAMA predicted reference set was created by using the genomes in the NCBI database, not the HMP set. The HMP set was used as the true answers only in evaluation. Therefore, we thought it is fair not using the HMP set in this experiment.<br><br>* Regarding N-PLR vs V-PLR, please add more discussion that helps the reader to understand the contrast between "51x" and "very high depth". At present, it would seem to me that reads aligning to a depth of 51x is not insignificant.<br><br>Response:<br>We highly appreciate this comment. While revising our manuscript, we found that there was an error while summarizing the numbers for Figure 2b and therefore Figure 2b was created incorrectly. In this revision, we replaced the original Figure 2b by a fixed one. Now the maximum depths of V-PLRs and N-PLRs are changed to 66x and 31x respectively (Lines 305-309).<br><br>In the original Figure 2b, we thought that the very high depth (more than a few hundreds) is possible because very large number of individuals of the same species can exist in a metagenomic sample. In reality, a recent study reported such species with a very high abundance from the same data used in our study (Supplementary Table 6 in Kuleshov, Volodymyr, et al. "Synthetic long-read sequencing reveals intraspecies diversity in the human microbiome." Nature biotechnology 34.1 (2016): 64-69). But because of this comment, we could find the error. We highly appreciate the reviewer again, and apologize for this error.<br><br>* For the section beginning at line 324: it would be clearer to refer to the “short-read only assembly” rather than “initial assembly”. As a simplification, the authors could consider introducing the acronym mapping “short-read -> SR” early on in their manuscript. This could then sit alongside “PLR” in the discussion, such as “SR assembly” vs “PLR assembly”.<br><br>Response:<br>We highly appreciate this comment and agree with the reviewer. We modified the word “initial assembly” to “SR assembly” where possible in our manuscript (Line numbers are not listed because so many lines are affected).<br><br>* Starting at line 333: I was quite sure what software was used to infer alignment blocks. Could this be made clearer, either here or in methods.<br><br>Response:

	We highly appreciate this comment and apologize for the missing information. We added what software was used to make alignment blocks in our manuscript (Lines 255-256).<br><br>* Starting at line 341 and figure 4. The comparison genome bin quality between SR-only and PLR assemblies worthwhile, but the means of visualizing this in figure 4c is strange. I would suggest the authors use four separate facets for the various metrics. This would be immediately interpretable and could just become a separate figure.<br><br>Response:<br>We highly appreciate this comment and agree with the reviewer. Figure 4c was complicated to understand because it had too much information. The specific values of statistics for what we wanted to show were already provided in Supplementary Table S6. Therefore, we thought that some of them were better to be shown in Supplementary Table S6. In this revision, we modified Figure 4c to display only two information: N50 and gene completeness. We also changed the description in our manuscript (Lines 359-363).<br><br>* Although long-read sequencing adds to the total cost, it is not prohibitively expensive and has become increasingly common within metagenomic studies. The authors could mention that multiple technologies means more time (cost) and a larger quantity of DNA, and that careful handling of DNA samples is required to produce good results (longer reads).<br><br>Response:<br>We highly appreciate this comment. We added more description in our manuscript (Lines 384-385).<br><br>* In the discussion, although an explicit subheading “Limitations” is not necessary, using this phrase in-text might be worthwhile. In particular, that the environment needs to be well studied and therefore haracterized with many high-quality reference genomes.<br><br>Response:<br>We highly appreciate this comment. We modified and added the description in the Discussion section (Lines 400-403).<br><br>* The authors could mention future directions. Have they considered retrospectively applying PLR-GEN to the many existing metagenomic projects?<br><br>Response:<br>We highly appreciate this comment. We added the description about future direction in the Discussion section (Lines 418-421).<br><br>* It would have been interesting if the authors had also chosen to test PLR-GEN in a setting with the number of reference genomes was small.<br><br>Response:<br>We highly appreciate this comment. As described in other comments above, we performed this experiment and included the result in this revision. Please find our response in other comments above.

Powered by Editorial Manager® and ProduXion Manager® from Aries Systems Corporation

Minor comments:

*   Across manuscript: There are many cases of the noun “metagenome” being used as an adjective, rather than the adjective “metagenomic”. The first case (metagenome assembly) is found in the abstract. If used sparingly, this could slip through as a compound noun, however there are too many examples of “metagenome” modifying other nouns (i.e. assembly, short-reads, sequences, etc) and it becomes distracting. I would encourage the authors to replace “metagenome” with “metagenomic” where possible.

Response:
We highly appreciate this comment. We modified the word “metagenome” to “metagenomic” where possible in our manuscript (Line numbers are not listed because so many lines are affected).

*   Across manuscript: Possibly a contentious point, but hyphenated compound nouns (such as "long-read" and "short-read") are easier to parse. I appreciate the authors would then have to decide between "pseudo long-read" and "pseudo-long-read". In fact, the use of hyphens for short- and long-reads is presently not consistent within the manuscript.

Response:
We highly appreciate this comment. For consistency, we decided to use the hyphen only in between “pseudo” and “long” like “pseudo-long read”.

*   Line 32: for the sentence containing “… and begun to be used …” modify to “… and [have] begun to be used …”

Response:
Corrected (Lines 32-33).

*   Line 55: "sea water" is one word "seawater".

Response:
Corrected (Line 55).

*   Line 57: this might read better as "... accurately discover the composition and function of microbes within the environment."

Response:
Corrected (Lines 57-58).

*   Line 69: “short read-based” should be changed to “short-read based”, as “based” is not part of a compound noun.

Response:
Corrected (Line 69).

*   Line 69: this sentence should be in past tense. "Indeed, short-read based metagenomic assemblies have been used in (or underpinned) many recent studies."

Response:

Powered by Editorial Manager® and ProduXion Manager® from Aries Systems Corporation

	Corrected (Line 69).<br><br><br>* Line 105: delete the first word “sequences” as it is clear enough already by the phrase “mapped to each reference genome” that we are dealing with sequences. This would also apply to the term “read sequences” which could be referred to as “reads”. There would likely be more examples of throughout the text.<br><br>Response:<br>We highly appreciate this comment. In this revision, we deleted all unnecessary “sequences” in our manuscript (Line numbers are not listed because so many lines are affected).<br><br><br>* Line 134-135: flip with/only order in the sentence to become: "… distinguish genome fragments [with only] a very small number nucleotide differences …"<br><br>Response:<br>Corrected (Lines 136-137).<br><br><br>* Line 142: change to “… to a single sequenced DNA fragment.”<br><br>Response:<br>Corrected (Line 144).<br><br><br>* Line 207: add a comma as follows: " microbial genome sequences[,] that covered …"<br><br>Response:<br>Corrected (Line 210).<br><br><br>* Line 293: The sentence beginning with "If a single N-PLR …" reads as conjecture. If the authors intended to say that the N-PLR will not (or is unlikely to) align to either genome when mismatch=4, then I would recommend rewording slightly.<br><br>Response:<br>We highly appreciate this comment. In this revision, we restated the statement (Lines 303-305) as follows.<br><br>From:<br>If a single N-PLR is generated by placing ‘N’ at the three positions with variation, it is possible that it cannot be mapped to the above two genomes without lowering the mismatch penalty.<br><br>To:<br>If a single N-PLR is generated by placing ‘N’ at the three positions with variation, it cannot be mapped to the above two genomes unless at least three mismatches are not allowed.<br><br><br>* Line 301: This heading could be simplified to "PLRs improve the quality of short-read metagenomic assemblies".<br><br>Response:<br>Corrected (Line 311).

	* Line 303: change "approaches" to "approach".<br><br>Response:<br>Corrected (Line 313).<br><br>* Line 304: change "advantages" to "advantage".<br><br>Response:<br>Corrected (Line 314).<br><br>* Line 312: "assembly sequences" could just be called "contigs" throughout the manuscript. Even though there is some end-product variation between assemblers, this is acceptable general parlance.<br><br>Response:<br>We highly appreciate this comment. In this revision, we changed all “assembly sequences” to “contigs” in our manuscript (Lines 322, 327, 649, and 651).<br><br>* Line 370: For the sentence beginning “Therefore, short-reads are still being …” insert “used” to become “Therefore, short-reads are still being [used] …”<br><br>Response:<br>Corrected (Line 386).<br><br>* Line 376: Insert "the" in the sentence beginning "One of [the] excellent features …" or change it to "An excellent feature of …"<br><br>Response:<br>Corrected (Line 392).
**Additional Information:**	
**Question**	**Response**
Are you submitting this manuscript to a special series or article collection?	No
**Experimental design and statistics**<br><br>Full details of the experimental design and statistical methods used should be given in the Methods section, as detailed in our Minimum Standards Reporting Checklist. Information essential to interpreting the data presented should be made available in the figure legends.<br><br>Have you included all the information requested in your manuscript?	Yes

Powered by Editorial Manager® and ProduXion Manager® from Aries Systems Corporation

**Resources**<br><br>A description of all resources used, including antibodies, cell lines, animals and software tools, with enough information to allow them to be uniquely identified, should be included in the Methods section. Authors are strongly encouraged to cite Research Resource Identifiers (RRIDs) for antibodies, model organisms and tools, where possible.<br><br>Have you included the information requested as detailed in our Minimum Standards Reporting Checklist?	Yes
**Availability of data and materials**<br><br>All datasets and code on which the conclusions of the paper rely must be either included in your submission or deposited in publicly available repositories (where available and ethically appropriate), referencing such data using a unique identifier in the references and in the “Availability of Data and Materials” section of your manuscript.<br><br>Have you have met the above requirement as detailed in our Minimum Standards Reporting Checklist?	Yes

Manuscript with DOI
Click here to access/download;Manuscript;manuscript_PLR-GEN_GS_rvfinal with DOI.docx

Generation and application of pseudo-long reads for metagenome assembly

Author: Mikang Sim, Jongin Lee, Suyeon Wy, Nayoung Park, Daehwan Lee, Daehong Kwon, and Jaebum Kim*

Department of Biomedical Science and Engineering, Konkuk University, Seoul 05029, Republic of Korea.

*To whom correspondence should be addressed.

Email addresses:

Mikang Sim: mg9022@konkuk.ac.kr

Jongin Lee: jongin33@konkuk.ac.kr

Suyeon Wy: lish96@konkuk.ac.kr

Nayoung Park: p3159@konkuk.ac.kr

Daehwan Lee: cjfcl@konkuk.ac.kr

Daehong Kwon: kwanyi01@konkuk.ac.kr

Jaebum Kim: jbkim@konkuk.ac.kr

Abstract

Background:

Metagenomic assembly using high-throughput sequencing data is a powerful method to construct microbial genomes in environmental samples without cultivation. However, metagenomic assembly, especially when only short reads are available, is a complex and challenging task because mixed genomes of multiple microorganisms constitute the metagenome. Although long read sequencing technologies have been developed and have begun to be used for metagenomic assembly, many metagenomic studies have been performed based on short reads because the generation of long reads requires higher sequencing cost than short reads.

Results:

In this study, we present a new method called PLR-GEN. It creates pseudo-long reads from metagenomic short reads based on given reference genome sequences by considering small sequence variations existing in individual genomes of the same or different species. When applied to a mock community dataset in the Human Microbiome Project, PLR-GEN dramatically extended short reads in length of 101 bp to pseudo-long reads with N50 of 33 Kbp and 0.4 % error rate. The use of these pseudo-long reads generated by PLR-GEN resulted in an obvious improvement of metagenomic assembly in terms of the number of sequences, assembly contiguity, and prediction of species and genes.

Conclusions:

PLR-GEN can be used to generate artificial long read sequences without spending extra sequencing cost, thus aiding various studies using metagenomes.

Keywords: next-generation sequencing, metagenomic assembly, pseudo-long read

Background

Metagenomic sequences containing all sequenced genetic materials in environmental samples are one of the most important resources for understanding the roles of microorganisms in an environment. Metagenomic sequences have been widely used for characterizing microbial communities in various environments, such as animal organs, seawater, hydrothermal environment, plants, and soils [1-6]. In studies using metagenomic sequences, the creation of high-quality metagenomic assemblies is critical to accurately discover the composition and function of microbes within the environment. Although several metagenomic assembly algorithms have been developed [7-12], the task of metagenomic assembly remains challenging because of the complexity of metagenomic sequences consisting of sequences of many short DNA fragments of diverse species [13, 14].

The development of third-generation sequencing technologies aiming to increase sequence length has provided new opportunities for metagenomic assembly because longer sequences are more useful for resolving repetitive genome sequences and distinguishing sequences from different species [15-18]. With recently developed hybrid assemblers that use long reads together with short reads, the contiguity of genome assemblies is increased while minimizing assembly errors [19, 20]. However, the generation of long reads requires relatively higher sequencing cost than short reads, thus limiting their applications for metagenomic assembly [16]. Indeed, short read based metagenomic assemblies have been used in many recent studies [6, 21-24].

In this situation, there was a recent effort to generate and use artificial long reads from real short reads to take advantage of the benefits of both short and long reads without needing extra sequencing cost. For example, a recently developed local *de novo* assembly tool called Konnector can generate elongated pseudo-long reads from paired-end tag (PET) sequencing data for a single genome assembly [25]. PET pseudo-long reads generated by Konnector have

been successfully used to assemble the genome of the American bullfrog [26]. However, this approach cannot be directly applied to metagenomic sequences because of the complexity of metagenomic sequences resulting from the large number of species present in the metagenomic sample and similar genomic regions with small sequence variations shared by different individuals in the same or different species [27]. To address this problem, known reference genomes of many microorganisms can be used as a valuable guide to correctly capture these subtle sequence variations when generating pseudo-long read sequences even though they cannot represent all microorganisms existing in a metagenomic sample.

As an attempt to fully utilize known reference genomes of many microbial species for metagenomic assembly, we present a new method called PLR-GEN for the generation of pseudo-long reads (PLRs) by using short reads of a metagenomic sample and genome sequences of known microbial species as input. PLR-GEN can capture subtle sequence variations originating from individual genomes of the same or different species and generate PLRs with small sequence variations. PLR-GEN was applied to short paired-end reads (2 x 101 bp) in the mock community dataset in the Human Microbiome Project [28, 29], and the PLRs with N50 of 33 Kbp were generated with 0.4 % error rate and 99.9 % alignment rate against reference genomes in the above dataset. When applied to metagenomic assembly, PLRs resulted in increased assembly contiguity without introducing assembly errors. They also improved recovery of species and genes in a metagenomic sample. This result clearly shows that PLR-GEN can generate very useful and accurate artificial long reads without spending extra sequencing cost. Thus, PLR-GEN can be successfully used for various studies involving metagenomes.

Methods

Generation of pseudo-long reads

Our method, PLR-GEN, generates pseudo-long reads (PLRs) through the following six steps (Fig. 1) based on next-generation sequencing short reads (single-end or paired-end reads) of a metagenomic sample and reference microbial genome sequences.

In the mapping & piling-up step, metagenomic short reads are mapped to each reference genomes separately using Bowtie2 [30] with default options. All aligned reads are filtered and piled-up on each reference genome using SAMtools mpileup [31] with '-q 20 --ff UNMAP,QCFAIL,DUP,SECONDARY' options. The '-q 20' option requires to use alignments with a minimum mapping quality 20, and the '--ff UNMAP,QCFAIL,DUP,SECONDARY' option makes unmapped, low-quality, duplicated, and secondary-aligned reads skipped. The mapping quality cutoff (-q) can be changed by the user.

In the PLR container construction step, reference genomes are broken at regions without having any mapped reads. Each of the resulting sequence fragments is defined as the PLR container which is a template for generating PLRs. Among initially generated PLR containers, small PLR containers shorter than 100 bp in length are discarded. The length cutoff can be changed by the user. Mapped reads in each PLR container are then used to generate PLR sequences in downstream steps.

In the PLR graph generation step, multiple read sequence alignment in each PLR container is converted to a graph, called a PLR graph, which consists of two types of nodes (normal and bubble) and directed edges representing the order of the nodes in the alignment. Specifically, a normal node is created from contiguous alignment columns where a single nucleotide occupies each alignment column. If an alignment column has two or more different nucleotides, then the bubble node is created for each different nucleotide. If there are only alignment columns with an identical base, then PLR graph is generated with only normal nodes and a single PLR sequence is created. In this step, some nucleotides with very low frequency can be ignored based on the relative frequency against the most frequent nucleotide in the alignment column.

By default, nucleotides with the relative frequency smaller than 0.5, which means that their absolute frequency is smaller than half of the most frequent one, are not used for generating the bubble node. The PLR graph generation panel in Fig. 1 shows an example of the PLR graph construction. Below the sequence of a reference genome (thick black line at the top) in one PLR container, multiple reads (thin lines with different colors) are aligned. Here, there are four alignment columns (B1 to B4 in Fig. 1) consisting of two different nucleotides. They are shown with aligned nucleotides, assuming that each of all other alignment columns is occupied by a single nucleotide. Two bubble nodes indicating two different aligned nucleotides in each alignment column are created. Five alignment regions flanking the above alignment columns B1 to B4 are used to create five normal nodes (named N1 to N5).

One of the excellent features of PLR-GEN is its ability to distinguish genome fragments with only a very small number of nucleotide differences from individuals of the same species or different species. This results in the generation of multiple PLR sequences with small variations in one PLR container if necessary. Generation of PLR sequences is done by finding one or more paths of nodes in the PLR graph. In the identified PLR path, only a single bubble node can be included in the path at a specific alignment position. In addition, bubble nodes at different alignment positions can be added together in the path in a dependent manner. This constraint reflects the fact that only specific combinations of variants at different alignment positions are possible, each of which corresponds to a single sequenced DNA fragment. Therefore, finding combinations of bubble nodes at different alignment positions is the main problem in PLR sequence generation. For doing that, special reads called bubble-linking reads that span two or more bubble nodes in different alignment columns (yellow lines in the PLR graph generation panel in Fig. 1) are identified, and the information of linked bubbles is then collected from the bubble-linking reads. The bubble linking information collected from one of bubble-linking reads r_i is then represented as the vector of bubble nodes with length of the

number of alignment columns with the bubble nodes B as follows:

$$V_i = (v_{i1}, \ldots, v_{iB}) \text{ where } v_{ij} \in \{\text{'A','C','G','T','}-\text{'}\}$$

In this equation, the '-' symbol is used to indicate the absence of linking information of bubble nodes in that position. Vectors obtained from bubble-linking reads in the example in Fig. 1 are shown at the right-hand side of the PLR graph generation panel. For example, the link from T in B1 to A in B2 is identified by the first bubble-linking read r_1 (the top-most yellow line in the PLR graph generation panel in Fig. 1). However, this read cannot provide any information for linking bubble nodes in the alignment column B3 or B4. Similarly, the link from T in B3 to T in B4 is discovered by the last bubble-linking read r_8 (the bottom-most yellow line in the same panel in Fig. 1) without any information for bubble nodes in B1 or B2. Note that Fig. 1 shows an example when single-end reads are used. When paired-end reads are used, the whole DNA fragment defined by the two paired reads is treated as a single unit of the bubble-linking read.

In the bubble combination identification step, vectors of bubble nodes created from each of bubble-linking reads in the previous step are used to find different combinations of all bubble nodes in the PLR graph (the bubble combination identification panel in Fig. 1). The basic idea is to cluster vectors of bubble nodes based on their consistency (the same nucleotide in the same vector element) and use each resulting cluster to define a specific combination of bubble nodes. For this purpose, a hierarchical agglomerative clustering algorithm, which iteratively clusters a pair of close data (or intermediate clusters) in hierarchical manner without needing a pre-specified number of clusters [32], is used. To perform clustering, the measure of distance $D(V_i, V_j)$ between two vectors of bubble nodes V_i and V_j for total B bubble nodes is defined as follows:

$$D(V_i, V_j) = \sum_{b=1}^{B} d_b \quad \text{where } d_b = \begin{cases} 1, & \text{if } v_{ib} \neq v_{jb} \text{ and } v_{ib} \neq \text{'}-\text{'} \text{ and } v_{jb} \neq \text{'}-\text{'} \\ 0, & \text{otherwise} \end{cases}$$

Note that the above distance can be defined only for two vectors that share at least one vector

element containing the same or different nucleotide in each vector. For two vectors not satisfying such a condition, an arbitrary distance larger than 1 is used to separate them to different clusters.

Based on calculated distances, hierarchical agglomerative clustering is carried out and final clusters are defined using 0 as a distance cutoff. For an example, at the bubble combination identification panel in Fig. 1, hierarchical clustering is performed for eight vectors of bubble nodes and two clusters are finally generated. All vectors in the same cluster have identical nucleotide at each bubble position or the '-' symbol if the bubble node at that position cannot be linked with any other bubble nodes at different positions. The latter case can happen when the distance between alignment columns with bubble nodes is too large to be linked by paired-end (or single-end) reads. From each cluster, vectors of bubble nodes are sorted by the position of the first bubble node in the PLR container. After that, the combination of bubble nodes is constructed by integrating bubble nodes at different alignment positions. At the position of an unlinked bubble node as described above, a '-' symbol is placed in the combination.

In the PLR path identification step, when all combinations of bubble nodes are identified, final PLR paths are generated by assembling flanking normal nodes and the identified consensus bubble paths. The PLR path identification panel in Fig. 1 shows two examples of PLR paths generated from two clusters obtained in the previous step.

Finally, in the PLR sequence generation step, for each PLR path, the final pseudo-long read sequence is constructed by concatenating nucleotides of normal and bubble nodes in the path. In this step, the 'N' symbol is used at the position of an unlinked bubble node.

Generation and evaluation of PLRs using the mock community dataset in the Human Microbiome Project

The mock community dataset in the Human Microbiome Project [15] (hereafter called the HMP dataset) was downloaded and used to generate PLRs. The quality and utility of these PLRs

were then evaluated. The HMP dataset consists of Illumina paired-end reads (2 × 101 bp; total 3.1 Gbp of 15,396,579 pairs of reads; NCBI accession number: SRR2822457) that were used to generate PLRs and known reference genomes that were used for the true assemblies in evaluation (Supplementary Table S1).

For generating PLRs from the short reads in the HMP dataset, the known reference genomes in the HMP dataset were not used. Instead, reference genomes were predicted using TAMA [33], a metagenomic sequence classification tool, with default options. This is an effort to mimic a real situation when reference genomes in a metagenomic sample are not known. From a total of 5,167 reference genomes included in the reference genome database of TAMA, a total of 615 different reference genomes were predicted. They are also existing in the HMP dataset (Supplementary Table S2). Using these 615 microbial genomes, that covered 77 % of species in the known reference genomes, PLRs were generated by PLR-GEN from the HMP dataset with default options as described in Supplementary Table S3. For comparison, PLRs were also generated by IDBA-UD [12] using the same short reads as input. Even though IDBA-UD is an assembler, not the generator of PLRs, long sequences are generated by local assembly in the middle of an assembly process in IDBA-UD, which were treated as PLRs.

The quality of the generated PLRs was then assessed using MetaQUAST [34] in terms of lengths of sequences, the number of sequences, total sequence length, and the number and total length of extremely long sequences (longer than 50 Kbp). In addition, MetaQUAST was used to evaluate the quality of PLRs in comparison with the known reference genomes in the HMP dataset (NCBI accession numbers in Supplementary Table S1) in terms of error rate and alignment lengths of PLRs against the known reference genomes. The error rate of PLRs was calculated based on the fraction of PLRs reported as "misassembled contigs" by MetaQUAST.

The effect and usefulness of PLRs for small sequence variation were then assessed. Among 684,388 PLR containers generated from the short reads in the HMP dataset using the predicted

reference genomes by TAMA, only 11,579 were used to generate more than one PLR with different combinations of bubble nodes as described in the previous subsection. From the above 11,579 PLR containers, a total of 29,291 PLRs were generated (hereafter V-PLRs). Further evaluation was performed for them. For comparison, additional PLRs, called N-PLRs, were created from V-PLRs by placing ‘N’ at all positions corresponding to bubble nodes. To compare V-PLRs and N-PLRs, they were mapped to the known reference genomes in the HMP dataset using minimap2 [35] with five different mismatch penalties (4, 6, 8, 10, and 12). Output alignments were filtered by mapping quality (≥ 20). Reference genome coverage was calculated and compared for both V-PLRs and N-PLRs using BEDTools [36]. In addition, the read depth distribution was calculated for both V-PLRs and N-PLRs and compared by aligning them to the known reference genomes in the HMP dataset using minimap2 with default options including mismatch penalty 4, which filters out the alignments with more than four mismatches.

Evaluation of PLRs based on metagenomic assembly

For checking the usefulness of PLRs for metagenomic assembly, four different assemblers, LINKS [37], metaSPAdes [20], OPERA-MS [19], and SSPACE-Longread [38], were used to generate metagenomic assemblies for the HMP dataset. In this evaluation, two versions of assemblies, a short read only assembly (SR assembly) using only short reads in the HMP dataset and a PLR assembly using both short reads and PLRs, were generated and compared. In the case of metaSPAdes, the SR assembly was generated with default options and the PLR assembly for both PLRs generated by PLR-GEN and IDBA-UD were constructed with default options except for ‘--nanopore’. OPERA-MS was first performed with default options. A file of intermediately generated contigs from Megahit, an embedded module in OPERA-MS, was used for the SR assembly. Final contigs generated by OPERA-MS were used for the PLR assembly with PLRs from PLR-GEN and IDBA-UD. In addition, using the SR assembly of metaSPAdes and OPERA-MS, additional long read scaffolding was carried out using LINKS

[37] and SSPACE-Longread [38] with default options.

The quality of metagenomic assemblies was assessed using various statistics, including the number of sequences, assembly contiguity, and the number of misassemblies, that were calculated by MetaQUAST [34] with default options after supplying the above known reference genomes present in the HMP dataset. For each known reference genome in the HMP dataset, alignment blocks of the SR and PLR assembly were generated using minimap2 embedded in MetaQUAST with the options set by MetaQUAST. Alignment blocks with a label "True" assigned by MetaQUAST were plotted using the Circlize R package [39].

Evaluation of PLRs based on metagenomic assembly binning

Each assembly generated in the previous subsection was binned using MetaBAT2 (v 2.12.1) [40] with default options except for '--minContig 1500'. Using alignments between assemblies and the known reference genomes in the HMP dataset prepared with MetaQUAST as described in the previous subsection, a species label corresponding to the known reference genome was assigned to each bin. In this step, if the sequence of a bin is aligned to more than one reference genome, the reference genome with the largest alignment coverage was chosen.

Additionally, the completeness (the best: 100 and the worst: 0) and contamination (the best: 0 and the worse: no upper bound) of bins were measured based on the single-copy marker gene content calculated with the lineage workflow in CheckM (v.1.1.2) [41] using default options. Because each bin is labeled independently by CheckM, the same species can be assigned to multiple bins. To compare the quality of bins at the species level, a single representative bin for each species was chosen based on the completeness score as described in a recent study [19]. All bins were categorized into four classes, "Complete", "High-quality", "Moderate", and "Incomplete", based on their quality of completeness and contamination. Specifically, a bin with completeness $\geq$ 90 and 0 contamination was defined as "Complete". A bin with completeness $\geq$ 80 and contamination $<$ 10 was defined as "High-quality". A bind with

completeness ≥ 50 and contamination < 20 was defined as "Moderate". All other bins were defined as "Incomplete". Genes in each bin were also predicted with Prodigal (v2.6.3) [42] using default options to examine gene completeness (the best: 100; the worst: 0) of the bin. Gene completeness was calculated based on the fraction of completely predicted genes.

Results

PLRs provide valuable information for metagenomic assembly in various aspects

Based on the predicted reference genomes and short paired-end reads (2 × 101 bp) in the HMP dataset (Methods), a total of 704,840 PLRs with a total length of 1,248 Mbp and N50 of 33 Kbp were obtained using PLR-GEN (Table 1). Among them, 3,332 PLRs were longer than 50 Kbp (more than 500-fold longer than the input reads). Their total length was 501 Mbp. The maximum length of PLRs was 1.2 Mbp (more than 12,000-fold longer than the input reads). From the alignment of PLRs to the known reference genomes in the HMP dataset, 99.9 % of bases in PLRs were successfully aligned to 52 % of reference genome bases. The error rate was only 0.422 % (Methods). Similar evaluation was also performed for the PLRs generated by IDBA-UD (Methods, Table 1), which indicates that the quality of PLRs produced by PLR-GEN is higher than the ones by IDBA-UD in terms of all measures examined.

In addition to its ability to elongate short reads with very low error rate as shown above, PLR-GEN can also distinguish genome fragments with only very small sequence variations, which can originate from individual genomes of the same or different species. PLR-GEN can generate multiple PLR sequences (hereafter called V-PLRs) with small sequence variations in one PLR container that represents such a genome fragment (Methods). In the evaluation with the HMP dataset, a total of 11,579 PLR containers generated 29,291 V-PLRs which were then compared with 11,579 N-PLRs created by placing 'N' at all positions of variation in V-PLRs (Methods). V-PLRs and N-PLRs were mapped to the known reference genomes in the HMP dataset with

various mismatch penalties, and V-PLRs could cover reference genomes more than 32 Kbp in average in comparison with N-PLRs (Supplementary Table S4). For example, in Fig. 2a, two V-PLRs created from the same PLR container having only three positions with different nucleotides were mapped to two different genomes of species, *S. agalactiae* and *S. mutans*. If a single N-PLR is generated by placing 'N' at the three positions with variation, it cannot be mapped to the above two genomes unless at least three mismatches are not allowed. Depths of mapped V-PLRs and N-PLRs on all known reference genomes using the alignments with less than four mismatches were then compared (Methods; Fig. 2b). Whereas V-PLRs could be mapped with high depth up to 66x, N-PLRs failed to map to the known reference genomes with a depth larger than 31x. These experiments clearly show that PLR-GEN can capture and use subtle sequence variations when generating PLRs to cover more regions of reference genomes.

PLRs improve the quality of short read metagenomic assembly

PLRs created from metagenomic short reads can be treated as general long reads and used in any assembly approach relying on long reads. This approach is particularly useful when only short reads are available. Researchers can take advantage of long reads for metagenomic assembly, which can be achieved by first generating PLRs from short reads by using PLR-GEN and then using PLRs for the metagenomic assembly.

To examine whether PLRs can improve the quality of metagenomic assembly, short read only metagenomic assemblies (SR assemblies) were constructed with metaSPAdes and OPERA-MS using short reads in the HMP dataset. They were further assembled to make PLR assemblies using PLRs generated by PLR-GEN (Methods). When the SR assembly was further assembled by metaSPAdes, the PLRs generated by PLR-GEN could (i) reduce the number of sequences (Fig. 3a), (ii) produce longer contigs consistent with the known reference genomes in the HMP dataset (Fig. 3b), and (iii) increase assembly contiguity (Fig. 3c) compared with the SR assembly. In addition, similar improvement was also observed when the further assembly was

done by OPERA-MS (Supplementary Table S5). For example, the number of sequences was reduced by 10 % when PLRs were used for the SR assembly with OPERA-MS (Supplementary Table S5). About two-fold increase of length was observed for long contigs. In addition, in terms of NA50, the corrected N50 calculated after breaking the SR assembly at misassembled regions against the known reference genomes, was increased 44 % and 41 % using PLRs for SR assemblies with metaSPAdes and OPERA-MS, respectively (Fig. 3b and Supplementary Table S5). This pattern was more prominent when additional long read based scaffolding tools, LINKS and SSPACE-Longread, were used (Supplementary Fig. S1 and Table S5). These results clearly demonstrate that PLRs can play an important role in increasing the quality of metagenomic assembly without relying on real long reads. The PLRs generated by IDBA-UD were also used to further assemble the SR assembly by the same assembly programs used above. Even though there was also a clear improvement from the SR assembly, the quality of the resulting assemblies was worse than the ones generated by the PLRs of PLR-GEN (Fig. 3 and Supplementary Table S5).

PLRs improve reconstructing microbial genomes and binning metagenomic assembly

Metagenomic assemblies can be used to reconstruct original microbial chromosome sequences, which can be further used for various downstream analyses, including metagenomic assembly binning and gene prediction. To evaluate the effect of improved metagenomic assemblies with PLRs for recovering original microbial chromosome sequences, the SR assembly and the PLR assembly generated with metaSPAdes were aligned against each of the known reference genomes in the HMP dataset (Methods). In most of those reference genomes, the PLR assembly could cover more contiguous regions (outer rings in Supplementary Fig. S2) than the SR assembly (inner rings in Supplementary Fig. S2). In the case of *S. mutans* (Fig. 4a), 99.56 % of its genome was covered by 21 alignment blocks created by the PLR assembly. However, 40 alignment blocks were used to cover similar genomic regions with the SR assembly.

Specifically, the longest alignment block created by the PLR assembly was 524 Kbp, which was more than two-fold longer than the longest one (237 Kbp) created by the SR assembly. Similar pattern was observed in another reference species of *R. sphaeroides* (Fig. 4b). Specifically, chromosome 2 of *R. sphaeroides* was covered by only three alignment blocks of the PLR assembly whereas 14 alignment blocks of the SR assembly were needed to cover chromosome 2 of *R. sphaeroides*.

To examine the usefulness of PLR-assisted metagenomic assemblies in other downstream analyses, both SR and PLR assemblies were binned, a species label was assigned to each bin, and the quality of each resulting bin was then evaluated for the assigned ten species (Methods). As shown in Fig. 4c and Supplementary Table S6, the PLR assembly increased the contiguity of binned sequences (bars in the left panel in Fig. 4c) without sacrificing bin completeness or contamination (Supplementary Table S6). In the case of gene completeness (bars in the right panel in Fig. 4c), the PLR assembly improved the quality of metagenomic bins in comparison with the SR assembly (from 95.91 % to 96.86 %; Fig. 4c and Supplementary Table S6). Specifically, for *R. sphaeroides* genome, N50 was increased more than five folds when the PLR assembly was used. In terms of bin completeness and contamination, the PLR assembly was effective in improving the bin quality of *S. epidermidis* from "High-quality" to "Complete". Additionally, the gene completeness was increased with PLRs for nine species-labeled bins. These findings indicate that the use of metagenomic assemblies generated with PLRs from PLR-GEN is useful for downstream analyses.

Discussion

In this study, we present a new method, called PLR-GEN, for generating pseudo-long reads (PLRs). PLRs are artificial long reads generated from next-generation sequencing short reads by utilizing microbial reference genomes. Our method was successfully applied to short reads

of 101 bp in length in the HMP dataset by creating PLRs with N50 of 33 Kbp that could be almost completely aligned to the known reference genomes in the HMP dataset with very low error rate (Fig. 2 and Table 1). For metagenomic assembly, the SR assemblies created by metaSPAdes and OPERA-MS were further assembled using PLRs, leading to dramatic improvement of resulting assemblies in terms of the number of sequences and assembly contiguity (Fig. 3). Assemblies improved by PLRs were also very useful for assembly binning and reconstruction of species genome (Fig. 4). These improved species genomes resulted in increased completeness of gene prediction (Fig. 4 and Supplementary Tables S6).

Sequenced long reads can provide long-range information. They are very helpful for metagenomic assembly. However, the sequencing of long reads is more expensive and requires more carefully handled and larger amount of DNA than generating short reads. This prevents their widespread use for metagenomic assembly. Therefore, short reads are still being used for metagenomic assembly and related studies [43-45]. In such situation, it is very helpful to generate long reads by just using short reads and reference genomes without needing extra sequencing cost. PLRs generated by our method can be used in many studies involving metagenomes, including studies based on assemblies as shown here by treating them as normal long reads such as PacBio [46] and Nanopore [47] reads.

One of the excellent features of our method is its ability to capture subtle sequence variations resulting from individual genomes in the same or different species in a metagenomic sample. This was achieved by (i) carefully identifying mapping positions of short reads occupied by multiple different nucleotides, (ii) representing them as vectors of sequence variations, and (iii) grouping them using a hierarchical clustering algorithm based on a newly designed distance measure. The effect of this feature was confirmed in comparison with PLRs generated by turning this feature off (Fig. 2). Therefore, PLRs generated by our method can also be used to discover information of haplotypes inherent in a metagenome [48].

One of the limitations of PLR-GEN is that because PLR-GEN creates PLRs by relying on given microbial reference genomes, the quality of PLRs depends on the number and quality of microbial reference genomes used. Therefore, to obtain high-quality PLRs, the environment needs to be well studied and characterized with many high-quality reference genomes. However, the improvement of assemblies by our PLRs generated using the small number of reference genomes was also observed from empirical experiments using randomly sampled reference genomes from the full reference genomes used in our study (Supplementary Tables S7 and S8). Another difficulty in metagenomic assembly is that there are multiple unknown microorganisms in a metagenomic sample sharing similar genomic regions with low sequence variations. In this situation, one important pre-processing step is to prepare the most appropriate reference genomes for a target metagenomic sample. This can be achieved by using recently developed metagenomic classifiers [33, 49-52] and collecting genomes of predicted species using those metagenomic classifiers. To this end, TAMA [33], one of metagenomic sequence classifiers, was used to prepare a set of reference genomes in the HMP dataset for making PLRs in this study. Another option is to use all microbial genomes in a public database such as NCBI, but it will take a lot of time and computer resources. However, continued accumulation of high-quality genome sequences of many microorganisms will make our method more valuable for studies involving metagenomes.

Finally, many existing metagenomic assemblies generated by only using short reads are good targets of PLR-GEN. As a future direction, those assemblies will be further improved by PLR-GEN and publicly released for being used by many researchers. In addition, PLR-GEN will be more optimized to more efficiently process a large number of reference genomes.

Availability of supporting source code and requirements

Project name: PLR-GEN

Project home page: https://github.com/jkimlab/PLR-GEN [53]

Operating system: Linux

Programming language: Perl

Other requirements:

License: MIT

Data Availability

The PLR-GEN package is available at: https://github.com/jkimlab/PLR-GEN [53]. Snapshots of our code and other data further supporting this work are openly available in the *GigaScience* repository, GigaDB [54].

Additional Files

Supplementary Table S1. NCBI accession numbers of the known reference genomes in the HMP dataset.

Supplementary Table S2. List of predicted reference genomes.

Supplementary Table S3. List of parameters of PLR-GEN used for evaluation.

Supplementary Table S4. Comparison of reference coverage between V-PLRs and N-PLRs.

Supplementary Table S5. Statistics of short read assemblies and assemblies improved by PLRs.

Supplementary Table S6. Quality of each bin of the SR and PLR assemblies.

Supplementary Table S7. Statistics of PLRs generated from short reads in the mock community dataset with different sets of reference genomes.

Supplementary Table S8. Statistics of short read assemblies and assemblies improved by PLRs with different sets of refence genomes.

Supplementary Fig. S1. metaSPAdes and OPERA-MS were separately used to create (i) the SR assembly only using short reads and (ii) the PLR assembly by further assembly with long read

scaffolding tools using PLRs. These two types of assemblies were compared in terms of (a) the number of sequences and (b) NA50.

Supplementary Fig. S2. Circos plots illustrating alignments of SR and PLR assemblies for genomes of all species in the HMP dataset. Inner (green color) and outer (orange color) circles represent SR and PLR assemblies, respectively.

List of abbreviations

PET: paired-end tag; PLRs: pseudo-long reads; HMP: Human Microbiome Project; SR: Short Read.

Ethics approval and consent to participate

Not applicable

Consent for publication

Not applicable

Competing interests

The author(s) declare no competing interests.

Funding

This paper was supported by Konkuk University Researcher Fund in 2021, a grant [2014M3C9A3063544] funded by the Ministry of Science and ICT of Korea, a grant [2019R1F1A1042018 and 2021M3H9A2097134] funded by the Ministry of Education of Korea, and a grant [PJ01334302] funded by the Rural Development Administration of Korea.

Authors' contributions

JBK conceived and designed the study. JBK, MKS, JIL, and DHL designed the PLR-GEN algorithm. MKS implemented the pseudo-long read generation algorithm. MKS, SYW, and NYP performed experiments. MKS, SYW, NYP, DHK, and JBK interpreted the analysis results. MKS drafted the manuscript. JBK finalized the manuscript. All authors approved the final manuscript.

Acknowledgements

Not applicable

References

1. Wang C, Li P, Yan Q, Chen L, Li T, Zhang W, et al. Characterization of the Pig Gut Microbiome and Antibiotic Resistome in Industrialized Feedlots in China. mSystems. 2019;4 6 doi:10.1128/mSystems.00206-19.

2. Almeida A, Mitchell AL, Boland M, Forster SC, Gloor GB, Tarkowska A, et al. A new genomic blueprint of the human gut microbiota. Nature. 2019;568 7753:499-504. doi:10.1038/s41586-019-0965-1.

3. Tully BJ, Graham ED and Heidelberg JF. The reconstruction of 2,631 draft metagenome-assembled genomes from the global oceans. Sci Data. 2018;5:170203. doi:10.1038/sdata.2017.203.

4. Wilkins LGE, Ettinger CL, Jospin G and Eisen JA. Metagenome-assembled genomes provide new insight into the microbial diversity of two thermal pools in Kamchatka, Russia. Sci Rep. 2019;9 1:3059. doi:10.1038/s41598-019-39576-6.

5. St John E, Flores GE, Meneghin J and Reysenbach AL. Deep-sea hydrothermal vent metagenome-assembled genomes provide insight into the phylum Nanoarchaeota. Environ Microbiol Rep. 2019;11 2:262-70. doi:10.1111/1758-2229.12740.

6. Bandla A, Pavagadhi S, Sridhar Sudarshan A, Poh MCH and Swarup S. 910 metagenome-assembled genomes from the phytobiomes of three urban-farmed leafy Asian greens. Sci Data. 2020;7 1:278. doi:10.1038/s41597-020-00617-9.

7. Nurk S, Meleshko D, Korobeynikov A and Pevzner PA. metaSPAdes: a new versatile metagenomic

assembler. Genome research. 2017;27 5:824-34.

8. Li D, Liu C-M, Luo R, Sadakane K and Lam T-W. MEGAHIT: an ultra-fast single-node solution for large and complex metagenomics assembly via succinct de Bruijn graph. Bioinformatics. 2015;31 10:1674-6.

9. Namiki T, Hachiya T, Tanaka H and Sakakibara Y. MetaVelvet: an extension of Velvet assembler to de novo metagenome assembly from short sequence reads. Nucleic acids research. 2012;40 20:e155-e.

10. Boisvert S, Raymond F, Godzaridis E, Laviolette F and Corbeil J. Ray Meta: scalable de novo metagenome assembly and profiling. Genome Biol. 2012;13 12:R122. doi:10.1186/gb-2012-13-12-r122.

11. Haider B, Ahn TH, Bushnell B, Chai J, Copeland A and Pan C. Omega: an overlap-graph de novo assembler for metagenomics. Bioinformatics. 2014;30 19:2717-22. doi:10.1093/bioinformatics/btu395.

12. Peng Y, Leung HC, Yiu SM and Chin FY. IDBA-UD: a de novo assembler for single-cell and metagenomic sequencing data with highly uneven depth. Bioinformatics. 2012;28 11:1420-8. doi:10.1093/bioinformatics/bts174.

13. Ayling M, Clark MD and Leggett RM. New approaches for metagenome assembly with short reads. Brief Bioinform. 2020;21 2:584-94. doi:10.1093/bib/bbz020.

14. Olson ND, Treangen TJ, Hill CM, Cepeda-Espinoza V, Ghurye J, Koren S, et al. Metagenomic assembly through the lens of validation: recent advances in assessing and improving the quality of genomes assembled from metagenomes. Brief Bioinform. 2019;20 4:1140-50. doi:10.1093/bib/bbx098.

15. Kuleshov V, Jiang C, Zhou W, Jahanbani F, Batzoglou S and Snyder M. Synthetic long-read sequencing reveals intraspecies diversity in the human microbiome. Nat Biotechnol. 2016;34 1:64-9. doi:10.1038/nbt.3416.

16. Xie H, Yang C, Sun Y, Igarashi Y, Jin T and Luo F. PacBio Long Reads Improve Metagenomic Assemblies, Gene Catalogs, and Genome Binning. Front Genet. 2020;11:516269. doi:10.3389/fgene.2020.516269.

17. Rhoads A and Au KF. PacBio Sequencing and Its Applications. Genomics Proteomics Bioinformatics. 2015;13 5:278-89. doi:10.1016/j.gpb.2015.08.002.

18. Moss EL, Maghini DG and Bhatt AS. Complete, closed bacterial genomes from microbiomes using nanopore sequencing. Nat Biotechnol. 2020;38 6:701-7. doi:10.1038/s41587-020-0422-6.

19. Bertrand D, Shaw J, Kalathiyappan M, Ng AHQ, Kumar MS, Li C, et al. Hybrid metagenomic assembly enables high-resolution analysis of resistance determinants and mobile elements in human microbiomes. Nat Biotechnol. 2019;37 8:937-44. doi:10.1038/s41587-019-0191-2.

20. Antipov D, Korobeynikov A, McLean JS and Pevzner PA. hybridSPAdes: an algorithm for hybrid assembly of short and long reads. Bioinformatics. 2016;32 7:1009-15. doi:10.1093/bioinformatics/btv688.

21. Damashek J, Edwardson CF, Tolar BB, Gifford SM, Moran MA and Hollibaugh JT. Coastal Ocean Metagenomes and Curated Metagenome-Assembled Genomes from Marsh Landing, Sapelo Island (Georgia, USA). Microbiol Resour Announc. 2019;8 40 doi:10.1128/MRA.00934-19.

22. Wilkinson T, Korir D, Ogugo M, Stewart RD, Watson M, Paxton E, et al. 1200 high-quality metagenome-assembled genomes from the rumen of African cattle and their relevance in the context of sub-optimal feeding. Genome Biol. 2020;21 1:229. doi:10.1186/s13059-020-02144-7.

23. Glendinning L, Stewart RD, Pallen MJ, Watson KA and Watson M. Assembly of hundreds of novel bacterial genomes from the chicken caecum. Genome Biol. 2020;21 1:34. doi:10.1186/s13059-020-1947-1.

24. Marques M, Borges N, Silva SG, da Rocha UN, Lago-Lestón A, Keller-Costa T, et al. Metagenome-Assembled Genome Sequences of Three Uncultured Planktomarina sp. Strains from the Northeast Atlantic Ocean. Microbiol Resour Announc. 2020;9 12 doi:10.1128/MRA.00127-20.

25. Vandervalk BP, Yang C, Xue Z, Raghavan K, Chu J, Mohamadi H, et al. Konnector v2.0: pseudo-long reads from paired-end sequencing data. BMC Med Genomics. 2015;8 Suppl 3:S1. doi:10.1186/1755-8794-8-S3-S1.

26. Hammond SA, Warren RL, Vandervalk BP, Kucuk E, Khan H, Gibb EA, et al. The North American bullfrog draft genome provides insight into hormonal regulation of long noncoding RNA. Nat Commun. 2017;8 1:1433. doi:10.1038/s41467-017-01316-7.

27. Nicholls SM, Aubrey W, De Grave K, Schietgat L, Creevey CJ and Clare A. On the complexity of haplotyping a microbial community. Bioinformatics. 2020;37 10:1360–6. doi:10.1093/bioinformatics/btaa977.

28. Consortium HMP. A framework for human microbiome research. Nature. 2012;486 7402:215-21. doi:10.1038/nature11209.

29. Consortium HMP. Structure, function and diversity of the healthy human microbiome. Nature. 2012;486 7402:207-14. doi:10.1038/nature11234.

30. Langmead B and Salzberg SL. Fast gapped-read alignment with Bowtie 2. Nature methods. 2012;9 4:357.

31. Danecek P, Bonfield JK, Liddle J, Marshall J, Ohan V, Pollard MO, et al. Twelve years of SAMtools and BCFtools. Gigascience. 2021;10 2 doi:10.1093/gigascience/giab008.

32. Zepeda-Mendoza ML and Resendis-Antonio O. Hierarchical Agglomerative Clustering. In: Dubitzky W, Wolkenhauer O, Cho K-H and Yokota H, editors. Encyclopedia of Systems Biology. New York, NY: Springer New York; 2013. p. 886-7.

33. Sim M, Lee J, Lee D, Kwon D and Kim J. TAMA: improved metagenomic sequence classification through meta-analysis. BMC Bioinformatics. 2020;21 1:185. doi:10.1186/s12859-020-3533-7.

34. Mikheenko A, Saveliev V and Gurevich A. MetaQUAST: evaluation of metagenome assemblies. Bioinformatics. 2016;32 7:1088-90. doi:10.1093/bioinformatics/btv697.

35. Li H. Minimap2: pairwise alignment for nucleotide sequences. Bioinformatics. 2018;34 18:3094-100. doi:10.1093/bioinformatics/bty191.

36. Quinlan AR and Hall IM. BEDTools: a flexible suite of utilities for comparing genomic features. Bioinformatics. 2010;26 6:841-2.

37. Warren RL, Yang C, Vandervalk BP, Behsaz B, Lagman A, Jones SJ, et al. LINKS: Scalable, alignment-free scaffolding of draft genomes with long reads. Gigascience. 2015;4:35. doi:10.1186/s13742-015-0076-3.

38. Boetzer M and Pirovano W. SSPACE-LongRead: scaffolding bacterial draft genomes using long read sequence information. BMC Bioinformatics. 2014;15:211. doi:10.1186/1471-2105-15-211.

39. Gu Z, Gu L, Eils R, Schlesner M and Brors B. circlize Implements and enhances circular visualization in R. Bioinformatics. 2014;30 19:2811-2. doi:10.1093/bioinformatics/btu393.

40. Kang DD, Li F, Kirton E, Thomas A, Egan R, An H, et al. MetaBAT 2: an adaptive binning algorithm for robust and efficient genome reconstruction from metagenome assemblies. PeerJ. 2019;7:e7359. doi:10.7717/peerj.7359.

41. Parks DH, Imelfort M, Skennerton CT, Hugenholtz P and Tyson GW. CheckM: assessing the quality of microbial genomes recovered from isolates, single cells, and metagenomes. Genome Res. 2015;25 7:1043-55. doi:10.1101/gr.186072.114.

42. Hyatt D, Chen GL, Locascio PF, Land ML, Larimer FW and Hauser LJ. Prodigal: prokaryotic gene recognition and translation initiation site identification. BMC Bioinformatics. 2010;11:119. doi:10.1186/1471-2105-11-119.

43. Zhou S, Luo R, Gong G, Wang Y, Gesang Z, Wang K, et al. Characterization of Metagenome-Assembled Genomes and Carbohydrate-Degrading Genes in the Gut Microbiota of Tibetan Pig. Front Microbiol. 2020;11:595066. doi:10.3389/fmicb.2020.595066.

44. Chen H, Liu C, Teng Y, Zhang Z, Chen Y and Yang Y. Environmental risk characterization and ecological process determination of bacterial antibiotic resistome in lake sediments. Environ Int. 2020;147:106345. doi:10.1016/j.envint.2020.106345.

45. Youngblut ND, de la Cuesta-Zuluaga J, Reischer GH, Dauser S, Schuster N, Walzer C, et al. Large-

Scale Metagenome Assembly Reveals Novel Animal-Associated Microbial Genomes, Biosynthetic Gene Clusters, and Other Genetic Diversity. mSystems. 2020;5 6 doi:10.1128/mSystems.01045-20.

46. Eid J, Fehr A, Gray J, Luong K, Lyle J, Otto G, et al. Real-time DNA sequencing from single polymerase molecules. Science. 2009;323 5910:133-8. doi:10.1126/science.1162986.

47. Jain M, Koren S, Miga KH, Quick J, Rand AC, Sasani TA, et al. Nanopore sequencing and assembly of a human genome with ultra-long reads. Nat Biotechnol. 2018;36 4:338-45. doi:10.1038/nbt.4060.

48. Nicholls SM, Aubrey W, De Grave K, Schietgat L, Creevey CJ and Clare A. On the complexity of haplotyping a microbial community. Bioinformatics. 2020; doi:10.1093/bioinformatics/btaa977.

49. Wood DE, Lu J and Langmead B. Improved metagenomic analysis with Kraken 2. Genome Biol. 2019;20 1:257. doi:10.1186/s13059-019-1891-0.

50. Lu J, Breitwieser FP, Thielen P and Salzberg SL. Bracken: estimating species abundance in metagenomics data. PeerJ Computer Science. 2017;3:e104.

51. Milanese A, Mende DR, Paoli L, Salazar G, Ruscheweyh HJ, Cuenca M, et al. Microbial abundance, activity and population genomic profiling with mOTUs2. Nat Commun. 2019;10 1:1014. doi:10.1038/s41467-019-08844-4.

52. Corvelo A, Clarke WE, Robine N and Zody MC. taxMaps: comprehensive and highly accurate taxonomic classification of short-read data in reasonable time. Genome Res. 2018;28 5:751-8. doi:10.1101/gr.225276.117.

53. PLR-GEN Package. https://github.com/jkimlab/PLR-GEN. Accessed 1 December 2021.

54. Sim M; Lee J; Wy S; Park N; Lee D; Kwon D; Kim J: Supporting data for "Generation and application of pseudo-long reads for metagenome assembly" GigaScience Database. 2022. http://dx.doi.org/10.5524/102214.

Figure Legends

Figure 1. Workflow of pseudo-long read generation (PLR-GEN). Using next-generation sequencing (NGS) short reads of a metagenomic sample and reference genomes as input, NGS reads are mapped to reference genomes. Mapped reads are pilled-up on aligned reference genome positions (the mapping & piling-up step). Reference genome regions with continuously mapped reads are defined as PLR containers (the PLR container construction step). The PLR graph is constructed using two types of nodes, normal nodes (N1 to N5) and bubble nodes (nodes corresponding to B1 to B4), and directed edges representing the order of nodes in the PLR container (the PLR graph generation step). Vectors of bubble nodes are created and grouped by the hierarchical clustering algorithm to identify various combinations of bubble nodes (the bubble combination identification step). Together with normal nodes flanking bubble nodes, each of different bubble node combinations is converted to a single PLR path (the PLR path identification step). Finally, a PLR sequence is constructed by concatenating nucleotides in normal and bubble nodes in each PLR path (the PLR sequence generation step).

Figure 2. Comparison of two types of PLRs generated from the mock community dataset in the Human Microbiome Project. (a) Two different V-PLRs, V-PLR1 and V-PLR2, generated from the same PLR container but with small sequence variations are mapped to two different reference genomes. (b) After mapping all V-PLRs and N-PLRs to reference genomes, their read depth distributions are plotted.

Figure 3. Comparison of assembly statistics generated by different assemblers for SR and PLR assemblies using the mock community dataset in the Human Microbiome Project. metaSPAdes was used to create (i) the SR assembly only using short reads and (ii) the PLR assembly by further assembling it using PLRs generated by PLR-GEN and IDBA-UD. These assemblies were compared in terms of (a) the number of sequences, (b) NA50 and (c) assembly contiguity.

Figure 4. Comparison of assemblies generated by metaSPAdes using the mock community dataset in the Human Microbiome Project in terms of (a, b) species genome reconstruction and (c) contig binning. SR and PLR assemblies were aligned and visualized using the Circlize R package [39] for (a) *S. mutans* and (b) *R. sphaeroides* genomes. (c) After binning contigs and finding representative bins for ten species, their quality was measured in terms of N50 (bars in the left panel), and gene completeness (bars in the right panel).

Tables

Table 1. Statistics of pseudo-long reads generated from short paired-end reads (2 × 101 bp) using the mock community dataset in the Human Microbiome Project

Tool for PLR generation	PLR-GEN	IDBA-UD
No. of sequence (sequence in length > 50 Kbp)	704,840 (3,332)	15,231 (91)
Total length (sequence in length > 50 Kbp)	1,248,362,272 (501,383,852)	34,829,201 (9667428)
Min	100	201
Max	1,241,545	392,374
N50	32,996	15,775
Total aligned length (% of aligned bases)[1]	1,247,305,552 (99.915 %)	34,757,930 (99.795%)
Genome fraction[2]	52.017 %	41.793%
Error rate[3]	0.422 %	3.664%

[1]Total length of aligned PLR bases to the known reference genomes in the mock community dataset.

[2]Coverage of the known reference genomes in the mock community dataset by aligned PLRs.

[3]The proportion of PLRs assigned as 'misassembled contigs' by MetaQUAST.

Figure1

Click here to access/download;Figure;Figure 1.pdf

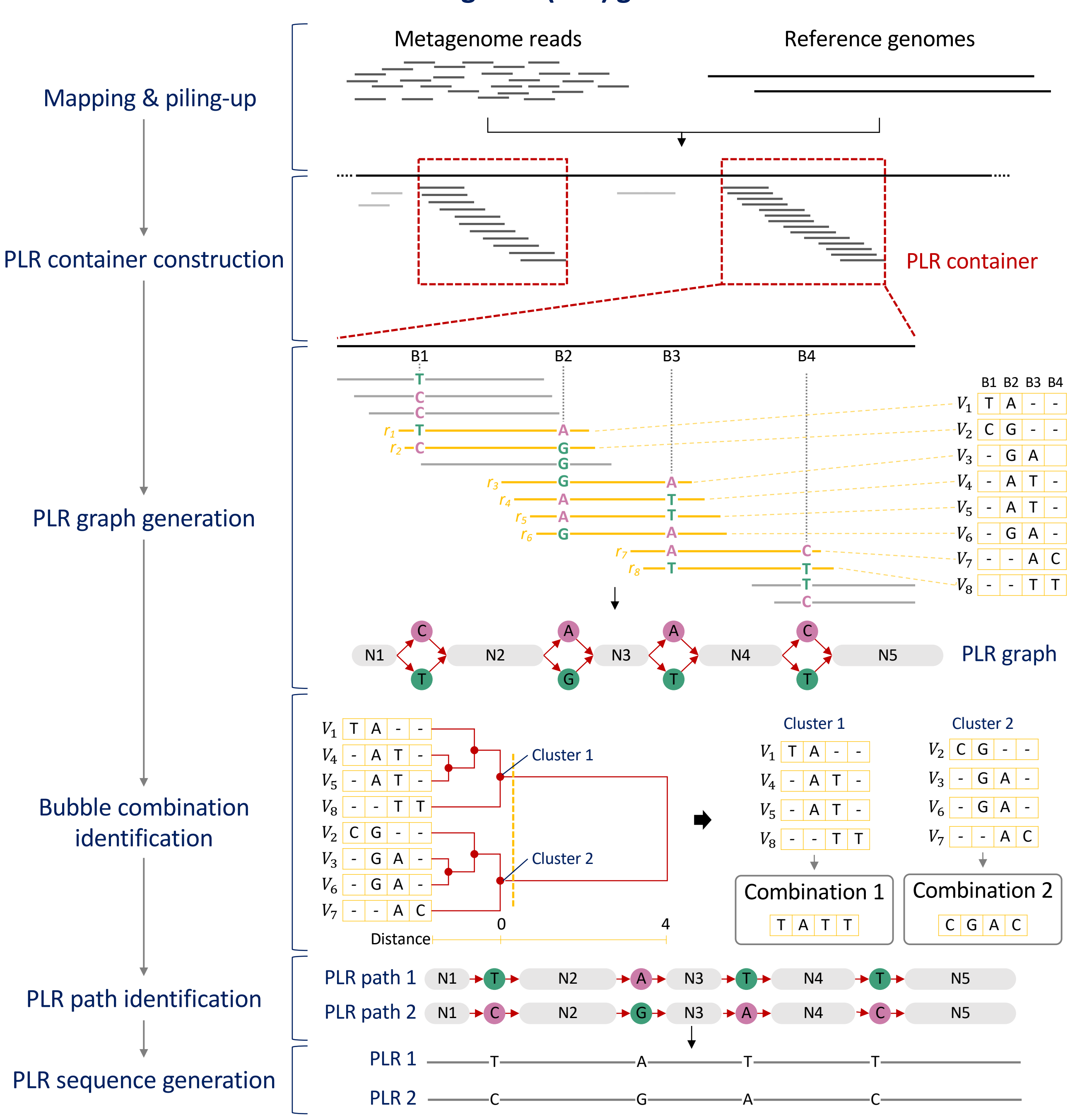

Figure2

Click here to access/download;Figure;Figure 2.pdf

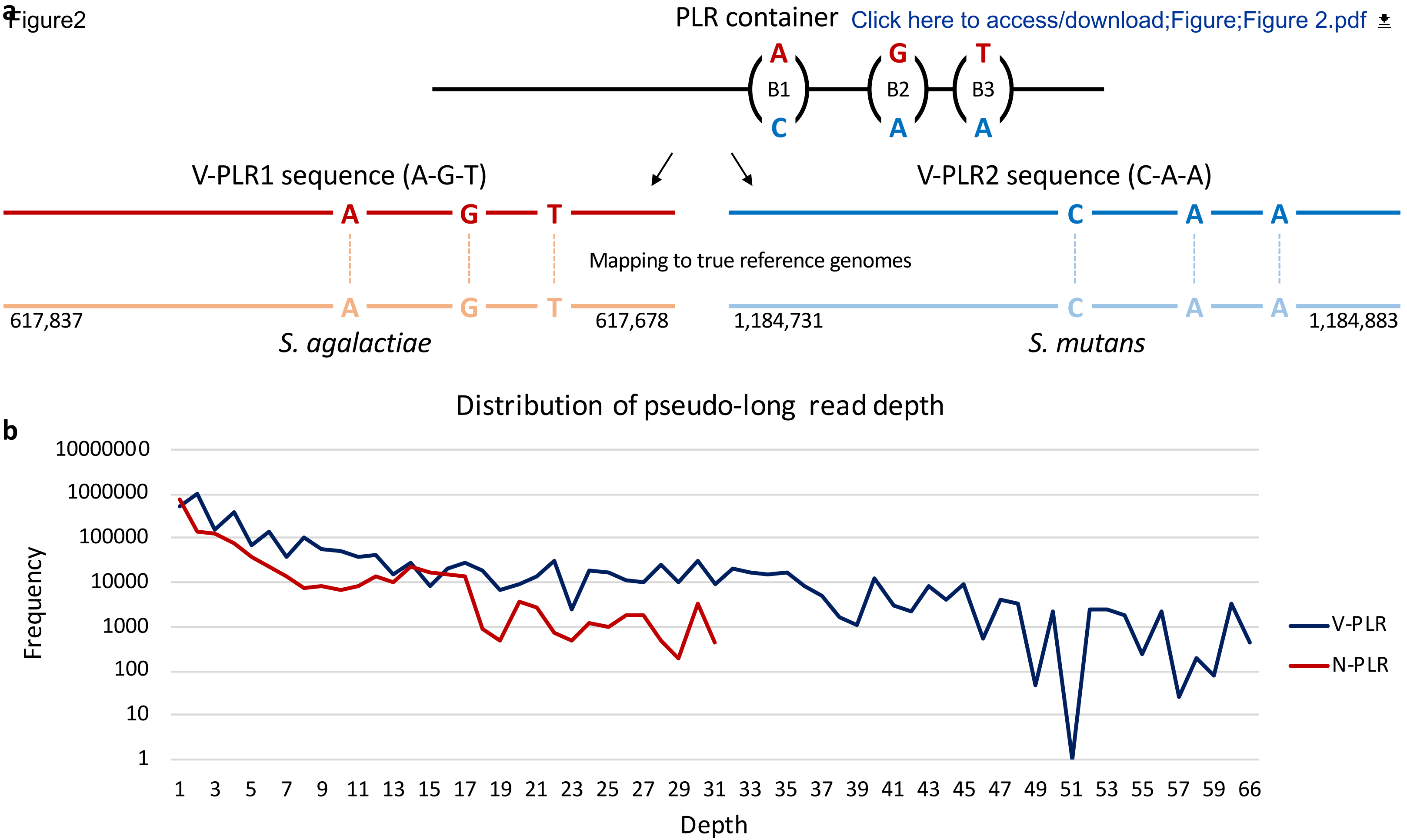

Figure3

Click here to access/download;Figure;Figure 3.pdf

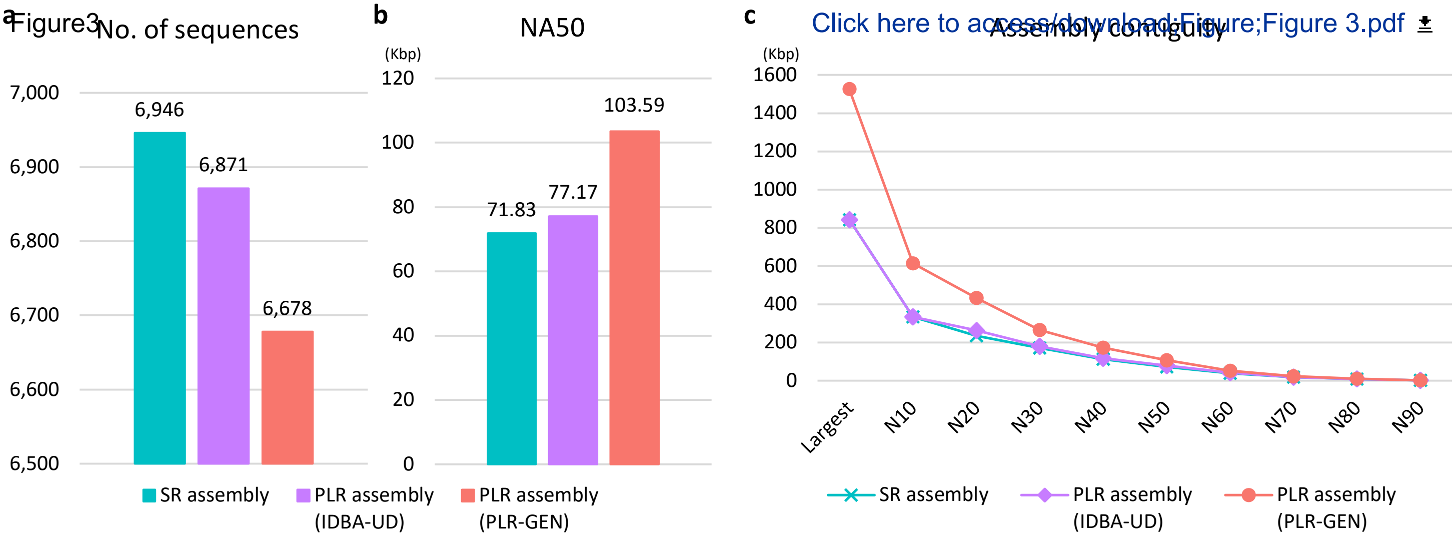

Figure4

Click here to access/download;Figure;Figure 4.pdf

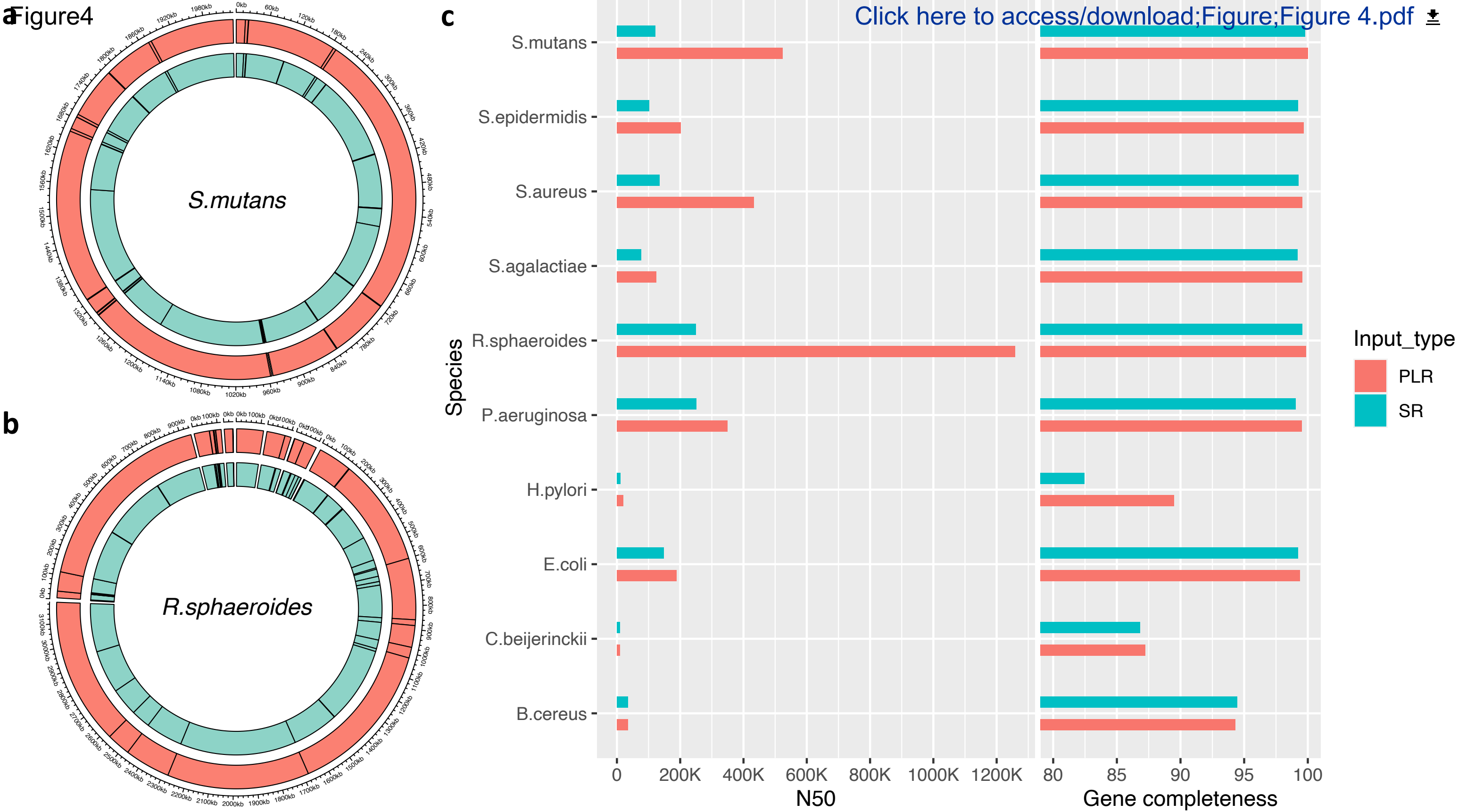

Supplementary data

Click here to access/download
Supplementary Material
Supplementary_data_rvfinal.xlsx

Supplementary figures

Click here to access/download
Supplementary Material
Supplementary_figures_rvfinal.docx
