# Supplementary material for: Generation and application of pseudo–long reads for metagenome assembly
Source: Gigascience. 2022 May 17;11:giac044. doi: 10.1093/gigascience/giac044 (PMC9112764; doi:10.1093/gigascience/giac044)
Supplement: giac044_Supplemental_Files [file giac044_supplemental_files.zip › Supplementary_figures_rvfinal.docx]

Supplementary Fig. S1. metaSPAdes and OPERA-MS were separately used to create (i) the SR assembly only using short reads and (ii) the PLR assembly by further assembly with long read scaffolding tools using PLRs. These two types of assemblies were compared in terms of (a) the number of sequences and (b) NA50.

Supplementary Fig. S2. Circos plots illustrating alignments of initial and PLR assemblies for genomes of all species in the HMP dataset. Inner (green color) and outer (orange color) circles represent initial and PLR assemblies, respectively.
