# Supplementary material for: Generation and application of pseudo–long reads for metagenome assembly
Source: Gigascience. 2022 May 17;11:giac044. doi: 10.1093/gigascience/giac044 (PMC9112764; doi:10.1093/gigascience/giac044)
Supplement: giac044_Response_to_Reviewer_Comments_Original_Submission [file giac044_response_to_reviewer_comments_original_submission.pdf]

Author's Response To Reviewer Comments

Close

Response to Reviewers

Reviewer #1

Authors present a new reference-guided local assembly method to be applied for metagenome analysis and microbial species identification. Although pseudo long reads built from short-reads have been used to the assembly problem of a single genome, no successful application for the meta-genomes.
Overall, the PLR_GEN method outperformed a simple assembly result in terms of the number of assembled sequences, the quality of the resulting assembly, and identification of species and genes.

However, i am not quite convinced why they have to build pseudo long reads to identify species and genes using the reference genomes. If there are reference genomes, they could simply map the short reads to the genomes rather making pseudo long reads.
They have to check whether the approach to use pseudo long reads are better than alternative way to map the short reads in terms of the quality of assembly and idenification of species and genes.

Response:
We highly appreciate the reviewer's comment and apologize for the confusion. The main purpose of generating PLRs is not just for the identification of species and genes. It was one of the experiments to show the usefulness of our PLRs. As you know, the reference genomes collected from NCBI only contain the genomes of known species. On the other hand, most of the species in a metagenomic sample are unknown, and therefore their genomes do not exist in the reference genomes. However, even though the complete genome of an unknown species does not exist in the reference genomes, it is highly probable that some portions of its genome exist in the reference genomes because different species can share highly similar genomic regions and it can happen between the known species in the reference genomes and the unknown species in a metagenomic sample. Therefore, the generated PLRs using the reference genomes can be parts of the genomes of unknown species in a metagenomic sample, and the PLRs can be used very effectively to construct accurate metagenomic assembly for the sample. This is the main motivation of our study.

However, because most of the species in a metagenomic sample are unknown, it is not easy to evaluate the generated PLRs and the assembly sequences constructed by using the PLRs. To address this problem, we chose the mock community dataset in the Human Microbiome Project, which is one of the most widely used dataset with complete species information in it for metagenomic studies, and showed the clear usefulness of our approach by performing various experiments including the identification of species and genes. Therefore, we are sure that our approach can contribute greatly to improve the quality of metagenomic assemblies.

Reviewer #2

General Comments:

The authors have presented a tool for generating pseudo-long-reads (PLR) from short-read data. The method relies on the availability of a sufficient number of relevant reference genomes. The authors demonstrate on a single, well characterised community that their approach produces generalised improvement in assembly quality metrics, which subsequently led to improvements in genome binning quality metrics.

This reviewer found their approach interesting and the algorithm description easy to comprehend. Although not discussed by the authors, this work recalls other analytical methods within metagenomics that attempt to encompass microbial variation.

The writing quality is of an acceptable level for communicating the authors findings, but any efforts to improve it further would benefit the reader.

Specific issues:
- The database they include in the repo with the 52 reference genomes is tailor-made to their test dataset. Granted its not impossible to do a naïve assembly, identify the taxa present, expand the reference genome database, and run this method, but something that integral to the method should be done by the tool, not manually.

Response:
We highly appreciate this comment and agree with the reviewer. In this revision, we added a new option (-tama) in PLR-GEN for automatically preparing reference genomes tailored to user's data by using TAMA, and updated the description in the Github page.

- Error rates between assemblies with and without PLR's, are not provided, only the error rate of the PLR sequences.

Response:
We highly appreciate this comment. We added the error rates for all assemblies with and without PLRs in Supplementary Table S5. In this revision, we added a new comparison with the PLRs generated by IDBA-UD. Therefore, the results of new comparison are also added in Table 1 and Supplementary Table S5.

Here we note that the error rates in Table 1 is more appropriate to compare PLR-GEN and IDBA-UD than the ones in Supplementary Table S5. It is because the error rates in Table 1 were obtained directly comparing the PLRs with true answers. However, the error rates in Supplementary Table S5 were calculated by comparing the assemblies generated by other scaffolding programs. Therefore, those numbers in Supplementary Table S5 can contain errors caused by the scaffolding programs (not completely by the used PLRs).

- See Figure 1 for an example of a paper that compares their approach to existing tools in the field: https://www.nature.com/articles/s41587-020-00777-4 . This paper lacks any external comparisons apart from running assemblers with and without their PLRs. This is useful, but needs to be assessed in context of current approaches, and in the context of error rates. See also this table 1 from another work: https://academic.oup.com/view-large/206266243 . Tools like IDBA-UD already do local assembly which could be considered PLR's. I'd want to see how they compare.

Response:
We highly appreciate this comment and agree with the reviewer. In this revision, we added the PLRs generated by IDBA-UD and compared them with ours in terms of the quality of raw sequences (Table 1) and the quality of assemblies generated by using them (Figure 3 and Supplementary Table S5).

We found that our PLRs were better than the ones created by IDBA-UD in terms of contiguity, coverage, and error rate (Table 1). In the comparison of assemblies generated by the PLRs, the assemblies generated by using our PLRs were better than the ones generated by using PLRs of IDBA-UD in terms of the number of sequences, contiguity, and coverage (Figure 3 and Supplementary Table S5). In addition, in all cases, NA50 of our results, which is the corrected N50 calculated after breaking sequences at misassembled positions, is better than NA50 of the results of IDBA-UD. NA50 is a kind of measure calculated by considering both contiguity and the amount of misassemblies.

We added description for the results of this additional comparison in our manuscript (Lines 212-215, 244, 248, 289-291, and 334-338).

- The authors discuss the high sequencing error rate of long reads, but recently the ONT folks have started to claim their assemblies are not improved by the addition of short reads. So long reads might be more expensive, but the error issue is becoming controversial at worst or null at best.

Response:
We highly appreciate this comment and agree with the reviewer. We know that there is a recent dramatic improvement of the quality of long reads, such as the development of HiFi reads. We mentioned the error rate of long reads because such high-quality long reads are not used widely in metagenomic assembly yet. But we agree that it is a controversial issue. Therefore, we removed the parts related with the error rates (Lines 34, 67-68, and 384).

- The authors claim that their method can capture "subtle sequence variations resulting from individual genomes in the same or difference species". While I don't doubt this to be true, it needs to be demonstrated for this dataset. Moreover, a more fair comparison would be to assemble against a database not derived from the test data.

Response:
We highly appreciate this comment and apologize for not accurately describing the results. We think it is already demonstrated in Figure 2. In Figure 2a, two PLRs with only three sequence variations were generated from the same PLR container, and they were successfully mapped to two different genomes of different species separately. In Figure 2b, we examined all V-PLRs (considering subtle sequence variations) and found that they can be mapped more widely than N-PLRs (not considering subtle sequence variations).

About the fair comparison, we think our original description is not good enough and confused the reviewer. Even though we used the short reads contained in the HMP dataset, the generation of PLRs was done without using any information of the known reference genomes in the HMP dataset. The reference genomes used for the PLR generation were extracted from the genomes in the NCBI database by a program called TAMA. Therefore, we think we can say that it is a fair comparison. We added description to clarify the way of data generation and comparison (Lines 201-203, and 224-225).

- The algorithmic approaches presented here are fascinating, and I would need more time to ingest. It seems very promising, and I love the idea of using hierarchical clustering to parse out taxonomic differences in the present of SNPs, but again I would need to see it in context.

Response:
We highly appreciate this positive comment. We will make our approach contribute more for better understanding metagenomes.

Specific Code/GitHub comments:
- I thank the authors for making their code available
- vendoring many different tools (see https://gist.github.com/datagrok/8577287 why vendoring is bad)

Response:
We highly appreciate this comment and the link of a good post about why vendoring is bad. Although our package includes several specific versions of third-party programs, they are stored as a whole as compressed files and can be downloaded at the time of installation. We also provide a separate code for their installation to help users for using our tool. We think this may be the best approach at this point for making our tool be used easily. But we will definitely figure out a better way when we update our tool.

And there were binary executable files in the “bin” directory in the original Github repository. They were accidentally added even though they are not necessary to be downloaded. In this revision, they were deleted completely.

- I was not able to get their docker container to run; I’m sure its possible, but their executable isn’t used by the CMD or RUN directive, nor is it in the PATH of the container

Response:
We highly appreciate this comment and apologize for this problem. In this revision, we added more explanation for running PLR-GEN with docker in the Github page, and added PATH for all executable codes in our docker image. In addition, our docker image was updated with the new option for preparing reference genomes using TAMA, and the docker repository was changed from mksim/plrgen to jkimlab/plrgen.

- tool isn't installable via conda - A conda recipe would be welcome, if all the authors were to do is document preparing an environment or better publishing an environment definition (yaml) within which all the dependencies are satisfied short of PLR-GEN itself.

Response:
We highly appreciate this comment and agree with the reviewer. In this revision, we created the conda environment definition file (plrgen-env.yml) with all the dependencies for running PLR-GEN, and added the description in the Github page.

- I'm not a Perl expert, but I can usually read enough to tell what code is doing. This code is uncommented, undocumented (apart from the README), and inscrutable to me. Recommend the authors read https://journals.plos.org/ploscompbiol/article?id=10.1371/journal.pcbi.1006561

Response:
We highly appreciate this comment. In this revision, we added more comments within the code.

- I couldn't get the tool to run, but looking at the code and reference database I am concerned with speed. The included bespoke set of reference genomes would likely need to be expanded for real-world use, and that could add significant runtime if not approached carefully.

Response:
We highly appreciate this comment and definitely agree with the reviewer. The number of reference genomes is very important because it affects the quality of the generated PLRs and runtime. And it is not easy to prepare the most appropriate reference genomes tailored to sequencing data that users want to analyze. Therefore, in this revision, we added a new option (-tama) for automatically preparing reference genomes tailored to users' data by using TAMA to address the above problems. In addition, if too many genomes are included in the created reference genome dataset, it is possible that the runtime of PLR-GEN becomes quite long. For handling this situation, we created a new option of PLR-GEN (-sampling), that can be used to randomly sample a given proportion of genomes from the prepared reference genome dataset. By using this option, users can reduce the runtime. Of course, if the amount of reference genomes is reduced, it affects the final quality of PLRs. However, based on our preliminary experiments, the small number of reference genomes does have an effect on improving the quality of assemblies (Supplementary Table S8). These results are also discussed in the Discussion section (Lines 404-407).

Major remarks:

* Line 71: The statement made here – that artificial long-reads are advantageous – has not yet been touched on by the authors, nor supported with external citations. As such, I feel this should be restated in less absolute terms or discussion which successfully makes the case should precede it.

Response:
We highly appreciate this comment and agree with the reviewer. In this revision, we restated the statement (Lines 71-73) as follows.

From:
In this situation, the generation and use of artificial long reads from real short reads can be a good

alternative to take advantage of benefits of both short and long reads without needing extra sequencing cost.

To:
In this situation, there was a recent effort to generate and use artificial long reads from real short reads to take advantage of the benefits of both short and long reads without needing extra sequencing cost.

* During container construction, it would seem that breaking reference genomes only at the point there is zero coverage is quite lenient. Particularly because I do not see a constraint on the mapping of reads beyond quality q=>20, although I must admit to a lack of familiarity with default bowtie options. Are the authors not concerned about partial alignments that will be more prevalent within regions of genomic plasticity.

Response:
We highly appreciate this comment and apologize for not accurately describing our method. When constructing the PLR container, we also used additional filtering option of SAMtools other than q>=20. It was presented as "--ff UNMAP,QCFAIL,DUP,SECONDARY" without any description. The meaning of the option is to discard unmapped and duplicated reads as well as secondary alignments. We believe that the situation mentioned by the reviewer is alleviated because of this additional filtering option. In this revision, we added the description of the option in our manuscript (Lines 107-109).

* How many references are required for PLR-GEN to be considered reliable? This would make it clear to the read if PLR-GEN could be applied to their project.

Response:
We highly appreciate this comment. Choosing the most appropriate number of reference genomes is not an easy task. But to check its effect empirically, we performed additional experiments by generating six more sets of PLRs using different number of reference genomes (10, 50, 100, 150, 300, and 500), which were created by random sampling from the TAMA predicted set of reference genomes, and the quality of generated assemblies by using them were compared (Supplementary Table S8). Here we found that even with only 10 reference genomes, there was an improvement of the generated assembly compared with the original SR assembly. We discussed this result in the Discussion section (Lines 404-407).

However, we know that this experimental result is not enough for making the most appropriate number of reference genomes. As mentioned in other comment above, we added a new option that can prepare the most appropriate reference genomes by using TAMA and genomes in the NCBI database, which can be also reduced by using the new random sampling option in PLR-GEN. We believe that these additional features will help users to address this issue to some degree.

* It would have been interesting to compare the PLR-GEN performance for the limited TAMA predicted reference set and the complete HMP set. I would imagine users will opt for what delivers the greatest performance, so long as it problem remains computationally feasible.

Response:
We highly appreciate this comment. As mentioned in the previous comment, additional experiments with various small numbers of reference genomes were performed, and their results were included in this revision. We hope this can be used as useful information for users. We note that the TAMA predicted reference set was created by using the genomes in the NCBI database, not the HMP set. The HMP set was used as the true answers only in evaluation. Therefore, we thought it is fair not using the HMP set in this experiment.

* Regarding N-PLR vs V-PLR, please add more discussion that helps the reader to understand the contrast between "51x" and "very high depth". At present, it would seem to me that reads aligning to a depth of 51x is not insignificant.

Response:
We highly appreciate this comment. While revising our manuscript, we found that there was an error while summarizing the numbers for Figure 2b and therefore Figure 2b was created incorrectly. In this revision, we replaced the original Figure 2b by a fixed one. Now the maximum depths of V-PLRs and N-PLRs are changed to 66x and 31x respectively (Lines 305-309).

In the original Figure 2b, we thought that the very high depth (more than a few hundreds) is possible because very large number of individuals of the same species can exist in a metagenomic sample. In reality, a recent study reported such species with a very high abundance from the same data used in our study (Supplementary Table 6 in Kuleshov, Volodymyr, et al. "Synthetic long-read sequencing reveals intraspecies diversity in the human microbiome." Nature biotechnology 34.1 (2016): 64-69). But because of this comment, we could find the error. We highly appreciate the reviewer again, and apologize for this error.

* For the section beginning at line 324: it would be clearer to refer to the “short-read only assembly” rather than “initial assembly”. As a simplification, the authors could consider introducing the acronym mapping “short-read -> SR” early on in their manuscript. This could then sit alongside “PLR” in the discussion, such as “SR assembly” vs “PLR assembly”.

Response:
We highly appreciate this comment and agree with the reviewer. We modified the word “initial assembly” to “SR assembly” where possible in our manuscript (Line numbers are not listed because so many lines are affected).

* Starting at line 333: I was quite sure what software was used to infer alignment blocks. Could this be made clearer, either here or in methods.

Response:
We highly appreciate this comment and apologize for the missing information. We added what software was used to make alignment blocks in our manuscript (Lines 255-256).

* Starting at line 341 and figure 4. The comparison genome bin quality between SR-only and PLR assemblies worthwhile, but the means of visualizing this in figure 4c is strange. I would suggest the authors use four separate facets for the various metrics. This would be immediately interpretable and could just become a separate figure.

Response:
We highly appreciate this comment and agree with the reviewer. Figure 4c was complicated to understand because it had too much information. The specific values of statistics for what we wanted to show were already provided in Supplementary Table S6. Therefore, we thought that some of them were better to be shown in Supplementary Table S6. In this revision, we modified Figure 4c to display only two information: N50 and gene completeness. We also changed the description in our manuscript (Lines 359-363).

* Although long-read sequencing adds to the total cost, it is not prohibitively expensive and has become increasingly common within metagenomic studies. The authors could mention that multiple technologies means more time (cost) and a larger quantity of DNA, and that careful handling of DNA samples is required to produce good results (longer reads).

Response:
We highly appreciate this comment. We added more description in our manuscript (Lines 384-385).

* In the discussion, although an explicit subheading “Limitations” is not necessary, using this phrase in-text might be worthwhile. In particular, that the environment needs to be well studied and therefore haracterized with many high-quality reference genomes.

Response:
We highly appreciate this comment. We modified and added the description in the Discussion section (Lines 400-403).

* The authors could mention future directions. Have they considered retrospectively applying PLR-GEN to the many existing metagenomic projects?

Response:
We highly appreciate this comment. We added the description about future direction in the Discussion section (Lines 418-421).

* It would have been interesting if the authors had also chosen to test PLR-GEN in a setting with the number of reference genomes was small.

Response:
We highly appreciate this comment. As described in other comments above, we performed this experiment and included the result in this revision. Please find our response in other comments above.

Minor comments:

* Across manuscript: There are many cases of the noun “metagenome” being used as an adjective, rather than the adjective “metagenomic”. The first case (metagenome assembly) is found in the abstract. If used sparingly, this could slip through as a compound noun, however there are too many examples of “metagenome” modifying other nouns (i.e. assembly, short-reads, sequences, etc) and it becomes distracting. I would encourage the authors to replace “metagenome” with “metagenomic” where possible.

Response:
We highly appreciate this comment. We modified the word “metagenome” to “metagenomic” where possible in our manuscript (Line numbers are not listed because so many lines are affected).

* Across manuscript: Possibly a contentious point, but hyphenated compound nouns (such as "long-read" and "short-read") are easier to parse. I appreciate the authors would then have to decide between "pseudo long-read" and "pseudo-long-read". In fact, the use of hyphens for short- and long-reads is presently not consistent within the manuscript.

Response:
We highly appreciate this comment. For consistency, we decided to use the hyphen only in between “pseudo” and “long” like “pseudo-long read”.

* Line 32: for the sentence containing “… and begun to be used …” modify to “… and [have] begun to be used …”

Response:
Corrected (Lines 32-33).

* Line 55: "sea water" is one word "seawater".

Response:
Corrected (Line 55).

* Line 57: this might read better as "... accurately discover the composition and function of microbes within the environment."

Response:
Corrected (Lines 57-58).

* Line 69: “short read-based” should be changed to “short-read based”, as “based” is not part of a compound noun.

Response:
Corrected (Line 69).

* Line 69: this sentence should be in past tense. "Indeed, short-read based metagenomic assemblies have been used in (or underpinned) many recent studies."

Response:
Corrected (Line 69).

* Line 105: delete the first word “sequences” as it is clear enough already by the phrase “mapped to each reference genome” that we are dealing with sequences. This would also apply to the term “read sequences” which could be referred to as “reads”. There would likely be more examples of throughout the text.

Response:
We highly appreciate this comment. In this revision, we deleted all unnecessary “sequences” in our manuscript (Line numbers are not listed because so many lines are affected).

* Line 134-135: flip with/only order in the sentence to become: "… distinguish genome fragments [with only] a very small number nucleotide differences …"

Response:
Corrected (Lines 136-137).

* Line 142: change to “… to a single sequenced DNA fragment.”

Response:
Corrected (Line 144).

* Line 207: add a comma as follows: " microbial genome sequences[,] that covered …"

Response:
Corrected (Line 210).

* Line 293: The sentence beginning with "If a single N-PLR …" reads as conjecture. If the authors intended to say that the N-PLR will not (or is unlikely to) align to either genome when mismatch=4, then I would recommend rewording slightly.

Response:
We highly appreciate this comment. In this revision, we restated the statement (Lines 303-305) as follows.

From:
If a single N-PLR is generated by placing 'N' at the three positions with variation, it is possible that it cannot be mapped to the above two genomes without lowering the mismatch penalty.

To:
If a single N-PLR is generated by placing 'N' at the three positions with variation, it cannot be mapped to the above two genomes unless at least three mismatches are not allowed.

* Line 301: This heading could be simplified to "PLRs improve the quality of short-read metagenomic assemblies".

Response:
Corrected (Line 311).

* Line 303: change "approaches" to "approach".

Response:
Corrected (Line 313).

* Line 304: change "advantages" to "advantage".

Response:
Corrected (Line 314).

* Line 312: "assembly sequences" could just be called "contigs" throughout the manuscript. Even though there is some end-product variation between assemblers, this is acceptable general parlance.

Response:
We highly appreciate this comment. In this revision, we changed all "assembly sequences" to "contigs" in our manuscript (Lines 322, 327, 649, and 651).

* Line 370: For the sentence beginning "Therefore, short-reads are still being …" insert "used" to become "Therefore, short-reads are still being [used] …"

Response:
Corrected (Line 386).

* Line 376: Insert "the" in the sentence beginning "One of [the] excellent features …" or change it to "An excellent feature of …"

Response:
Corrected (Line 392).

Close
