# Supplementary material for: Generation and application of pseudo–long reads for metagenome assembly
Source: Gigascience. 2022 May 17;11:giac044. doi: 10.1093/gigascience/giac044 (PMC9112764; doi:10.1093/gigascience/giac044)
Supplement: giac044_Reviewer_1_Report_Original_Submission — Jin-Wu Nam -- 12/2/2021 Reviewed [file giac044_reviewer_1_report_original_submission.pdf]

Reviewer Report

Title: Generation and application of pseudo-long reads for metagenome assembly

Version: Original Submission **Date:** 12/2/2021

Reviewer name: Jin-Wu Nam

Reviewer Comments to Author:

Authors present a new reference-guided local assembly method to be applied for metagenome analysis and microbial species identification. Although pseudo long reads built from short-reads have been used to the assembly problem of a single genome, no successful application for the meta-genomes.
Overall, the PLR_GEN method outperformed a simple assembly result in terms of the number of assembled sequences, the quality of the resulting assembly, and identification of species and genes. However, i am not quite convinced why they have to build pseudo long reads to identify species and genes using the reference genomes. If there are reference genomes, they could simply map the short reads to the genomes rather making pseudo long reads. They have to check whether the approach to use pseudo long reads are better than alternative way to map the short reads in terms of the quality of assembly and idenification of species and genes.

Level of Interest

Please indicate how interesting you found the manuscript: Choose an item.

Quality of Written English

Please indicate the quality of language in the manuscript: Choose an item.

Declaration of Competing Interests

Please complete a declaration of competing interests, considering the following questions:

- Have you in the past five years received reimbursements, fees, funding, or salary from an organisation that may in any way gain or lose financially from the publication of this manuscript, either now or in the future?
- Do you hold any stocks or shares in an organisation that may in any way gain or lose financially from the publication of this manuscript, either now or in the future?
- Do you hold or are you currently applying for any patents relating to the content of the manuscript?
- Have you received reimbursements, fees, funding, or salary from an organization that holds or has applied for patents relating to the content of the manuscript?
- Do you have any other financial competing interests?
- Do you have any non-financial competing interests in relation to this paper?

If you can answer no to all of the above, write 'I declare that I have no competing interests' below. If your reply is yes to any, please give details below.

I declare that I have no competing interests

I agree to the open peer review policy of the journal. I understand that my name will be included on my report to the authors and, if the manuscript is accepted for publication, my named report including any attachments I upload will be posted on the website along with the authors' responses. I agree for my report to be made available under an Open Access Creative Commons CC-BY license (http://creativecommons.org/licenses/by/4.0/). I understand that any comments which I do not wish to be included in my named report can be included as confidential comments to the editors, which will not be published.

Choose an item.

To further support our reviewers, we have joined with Publons, where you can gain additional credit to further highlight your hard work (see: https://publons.com/journal/530/gigascience). On publication of this paper, your review will be automatically added to Publons, you can then choose whether or not to claim your Publons credit. I understand this statement.

Yes Choose an item.
