# Supplementary material for: Generation and application of pseudo–long reads for metagenome assembly
Source: Gigascience. 2022 May 17;11:giac044. doi: 10.1093/gigascience/giac044 (PMC9112764; doi:10.1093/gigascience/giac044)
Supplement: giac044_Reviewer_2_Report_Original_Submission — Matthew Zachariah DeMaere, Ph.D -- 12/13/2021 Reviewed [file giac044_reviewer_2_report_original_submission.pdf]

Reviewer Report

Title: Generation and application of pseudo-long reads for metagenome assembly

Version: Original Submission **Date:** 12/13/2021

Reviewer name: Matthew Zachariah DeMaere, Ph.D

Reviewer Comments to Author:

Review
Title: Generation and application of pseudo-long reads for metagenome assembly
Manuscript #: GIGA-D-21-00349
General Comments:
The authors have presented a tool for generating pseudo-long-reads (PLR) from short-read data. The method relies on the availability of a sufficient number of relevant reference genomes. The authors demonstrate on a single, well characterised community that their approach produces generalised improvement in assembly quality metrics, which subsequently led to improvements in genome binning quality metrics.
This reviewer found their approach interesting and the algorithm description easy to comprehend. Although not discussed by the authors, this work recalls other analytical methods within metagenomics that attempt to encompass microbial variation.
The writing quality is of an acceptable level for communicating the authors findings, but any efforts to improve it further would benefit the reader.
Specific issues:
- The database they include in the repo with the 52 reference genomes is tailor-made to their test dataset. Granted its not impossible to do a naÃ¯ve assembly, identify the taxa present, expand the reference genome database, and run this method, but something that integral to the method should be done by the tool, not manually.
- Error rates between assemblies with and without PLR's, are not provided, only the error rate of the PLR sequences.
- See Figure 1 for an example of a paper that compares their approach to existing tools in the field: https://www.nature.com/articles/s41587-020-00777-4 . This paper lacks any external comparisons apart from running assemblers with and without their PLRs. This is useful, but needs to be assessed in context of current approaches, and in the context of error rates. See also this table 1 from another work: https://academic.oup.com/view-large/206266243 . Tools like IDBA-UD already do local assembly which could be considered PLR's. I'd want to see how they compare.
- The authors discuss the high sequencing error rate of long reads, but recently the ONT folks have started to claim their assemblies are not improved by the addition of short reads. So long reads might be more expensive, but the error issue is becoming controversial at worst or null at best.
- The authors claim that their method can capture "subtle sequence variations resulting from individual genomes in the same or difference species". While I don't doubt this to be true, it needs to be demonstrated for this dataset. Moreover, a more fair comparison would be to assemble against a

database not derived from the test data.
- The algorithmic approaches presented here are fascinating, and I would need more time to ingest. It seems very promising, and I love the idea of using hierarchical clustering to parse out taxonomic differences in the present of SNPs, but again I would need to see it in context.
Specific Code/GitHub comments:
- I thank the authors for making their code available
- vendoring many different tools (see https://gist.github.com/datagrok/8577287 why vendoring is bad)
- I was not able to get their docker container to run; I'm sure its possible, but their executable isn't used by the CMD or RUN directive, nor is it in the PATH of the container
- tool isn't installable via conda - A conda recipe would be welcome, if all the authors were to do is document preparing an environment or better publishing an environment definition (yaml) within which all the dependencies are satisfied short of PLR-GEN itself.
- I'm not a Perl expert, but I can usually read enough to tell what code is doing. This code is uncommented, undocumented (apart from the README), and inscrutable to me. Recommend the authors read https://journals.plos.org/ploscompbiol/article?id=10.1371/journal.pcbi.1006561
- I couldn't get the tool to run, but looking at the code and reference database I am concerned with speed. The included bespoke set of reference genomes would likely need to be expanded for real-world use, and that could add significant runtime if not approached carefully.
Major remarks:
* Line 71: The statement made here - that artificial long-reads are advantageous -- has not yet been touched on by the authors, nor supported with external citations. As such, I feel this should be restated in less absolute terms or discussion which successfully makes the case should precede it.
* During container construction, it would seem that breaking reference genomes only at the point there is zero coverage is quite lenient. Particularly because I do not see a constraint on the mapping of reads beyond quality q=>20, although I must admit to a lack of familiarity with default bowtie options. Are the authors not concerned about partial alignments that will be more prevalent within regions of genomic plasticity.
* How many references are required for PLR-GEN to be considered reliable? This would make it clear to the read if PLR-GEN could be applied to their project.
* It would have been interesting to compare the PLR-GEN performance for the limited TAMA predicted reference set and the complete HMP set. I would imagine users will opt for what delivers the greatest performance, so long as it problem remains computationally feasible.
* Regarding N-PLR vs V-PLR, please add more discussion that helps the reader to understand the contrast between "51x" and "very high depth". At present, it would seem to me that reads aligning to a depth of 51x is not insignificant.
* For the section beginning at line 324: it would be clearer to refer to the "short-read only assembly" rather than "initial assembly". As a simplification, the authors could consider introducing the acronym mapping "short-read -> SR" early on in their manuscript. This could then sit alongside "PLR" in the discussion, such as "SR assembly" vs "PLR assembly".
* Starting at line 333: I was quite sure what software was used to infer alignment blocks. Could this be made clearer, either here or in methods.
* Starting at line 341 and figure 4. The comparison genome bin quality between SR-only and PLR

assemblies worthwhile, but the means of visualising this in figure 4c is strange. I would suggest the authors use four separate facets for the various metrics. This would be immediately interpretable and could just become a separate figure.
* Although long-read sequencing adds to the total cost, it is not prohibitively expensive and has become increasingly common within metagenomic studies. The authors could mention that multiple technologies means more time (cost) and a larger quantity of DNA, and that careful handling of DNA samples is required to produce good results (longer reads).
* In the discussion, although an explicit subheading "Limitations" is not necessary, using this phrase in-text might be worthwhile. In particular, that the environment needs to be well studied and therefore characterised with many high-quality reference genomes.
* It would have been interesting if the authors had also chosen to test PLR-GEN in a setting with the number of reference genomes was small.
* The authors could mention future directions. Have they considered retrospectively applying PLR-GEN to the many existing metagenomic projects?
Minor comments:
* Across manuscript: There are many cases of the noun "metagenome" being used as an adjective, rather than the adjective "metagenomic". The first case (metagenome assembly) is found in the abstract. If used sparingly, this could slip through as a compound noun, however there are too many examples of "metagenome" modifying other nouns (i.e. assembly, short-reads, sequences, etc) and it becomes distracting. I would encourage the authors to replace "metagenome" with "metagenomic" where possible.
* Across manuscript: Possibly a contentious point, but hyphenated compound nouns (such as "long-read" and "short-read") are easier to parse. I appreciate the authors would then have to decide between "pseudo long-read" and "pseudo-long-read". In fact, the use of hyphens for short- and long-reads is presently not consistent within the manuscript.
* Line 32: for the sentence containing "… and begun to be used …" modify to "… and [have] begun to be used …"
* Line 55: "sea water" is one word "seawater".
* Line 57: this might read better as "... accurately discover the composition and function of microbes within the environment."
* Line 69: "short read-based" should be changed to "short-read based", as "based" is not part of a compound noun.
* Line 69: this sentence should be in past tense. "Indeed, short-read based metagenomic assemblies have been used in (or underpinned) many recent studies."
* Line 105: delete the first word "sequences" as it is clear enough already by the phrase "mapped to each reference genome" that we are dealing with sequences. This would also apply to the term "read sequences" which could be referred to as "reads". There would likely be more examples of throughout the text.
* Line 134-135: flip with/only order in the sentence to become: "… distinguish genome fragments [with only] a very small number nucleotide differences …"
* Line 142: change to "… to a single sequenced DNA fragment."
* Line 207: add a comma as follows: " microbial genome sequences[,] that covered …"

* Line 293: The sentence beginning with "If a single N-PLR …" reads as conjecture. If the authors intended to say that the N-PLR will not (or is unlikely to) align to either genome when mismatch=4, then I would recommend rewording slightly.
* Line 301: This heading could be simplified to "PLRs improve the quality of short-read metagenomic assemblies".
* Line 303: change "approaches" to "approach".
* Line 304: change "advantages" to "advantage".
* Line 312: "assembly sequences" could just be called "contigs" throughout the manuscript. Even though there is some end-product variation between assemblers, this is acceptable general parlance.
* Line 370: For the sentence beginning "Therefore, short-reads are still being …" insert "used" to become "Therefore, short-reads are still being [used] …"
* Line 376: Insert "the" in the sentence beginning "One of [the] excellent features …" or change it to "An excellent feature of …"

Level of Interest

Please indicate how interesting you found the manuscript: Choose an item.

Quality of Written English

Please indicate the quality of language in the manuscript: Choose an item.

Declaration of Competing Interests

Please complete a declaration of competing interests, considering the following questions:

- Have you in the past five years received reimbursements, fees, funding, or salary from an organisation that may in any way gain or lose financially from the publication of this manuscript, either now or in the future?
- Do you hold any stocks or shares in an organisation that may in any way gain or lose financially from the publication of this manuscript, either now or in the future?
- Do you hold or are you currently applying for any patents relating to the content of the manuscript?
- Have you received reimbursements, fees, funding, or salary from an organization that holds or has applied for patents relating to the content of the manuscript?
- Do you have any other financial competing interests?
- Do you have any non-financial competing interests in relation to this paper?

If you can answer no to all of the above, write 'I declare that I have no competing interests' below. If your reply is yes to any, please give details below.

I declare that I have no competing interests

I agree to the open peer review policy of the journal. I understand that my name will be included on my report to the authors and, if the manuscript is accepted for publication, my named report including any attachments I upload will be posted on the website along with the authors' responses. I agree for my report to be made available under an Open Access Creative Commons CC-BY license (http://creativecommons.org/licenses/by/4.0/). I understand that any comments which I do not wish to be included in my named report can be included as confidential comments to the editors, which will not be published.

Choose an item.

To further support our reviewers, we have joined with Publons, where you can gain additional credit to further highlight your hard work (see: https://publons.com/journal/530/gigascience). On publication of this paper, your review will be automatically added to Publons, you can then choose whether or not to claim your Publons credit. I understand this statement.

Yes Choose an item.
